# ChemChaste: Simulating spatially inhomogeneous biochemical reaction–diffusion systems for modeling cell–environment feedbacks

**DOI:** 10.1093/gigascience/giac051

**Published:** 2022-06-17

**Authors:** Connah G M Johnson, Alexander G Fletcher, Orkun S Soyer

**Affiliations:** Mathematics of Real-World Systems Doctoral Training Centre, University of Warwick, Coventry, CV35 9EF, UK; School of Life Sciences, University of Warwick, Coventry, CV35 9EF, UK; School of Mathematics & Statistics, University of Sheffield, Sheffield, S3 7RH, UK; Bateson Centre, University of Sheffield, Sheffield, S10 2TN, UK; School of Life Sciences, University of Warwick, Coventry, CV35 9EF, UK

**Keywords:** Chaste, biofilms, microbial communities

## Abstract

**Background:**

Spatial organization plays an important role in the function of many biological systems, from cell fate specification in animal development to multistep metabolic conversions in microbial communities. The study of such systems benefits from the use of spatially explicit computational models that combine a discrete description of cells with a continuum description of one or more chemicals diffusing within a surrounding bulk medium. These models allow the *in silico* testing and refinement of mechanistic hypotheses. However, most existing models of this type do not account for concurrent bulk and intracellular biochemical reactions and their possible coupling.

**Conclusions:**

Here, we describe ChemChaste, an extension for the open-source C++ computational biology library Chaste. ChemChaste enables the spatial simulation of both multicellular and bulk biochemistry by expanding on Chaste’s existing capabilities. In particular, ChemChaste enables (i) simulation of an arbitrary number of spatially diffusing chemicals, (ii) spatially heterogeneous chemical diffusion coefficients, and (iii) inclusion of both bulk and intracellular biochemical reactions and their coupling. ChemChaste also introduces a file-based interface that allows users to define the parameters relating to these functional features without the need to interact directly with Chaste’s core C++ code. We describe ChemChaste and demonstrate its functionality using a selection of chemical and biochemical exemplars, with a focus on demonstrating increased ability in modeling bulk chemical reactions and their coupling with intracellular reactions.

**Availability and implementation:**

ChemChaste version 1.0 is a free, open-source C++ library, available via GitHub at https://github.com/OSS-Lab/ChemChaste under the BSD license, on the Zenodo archive at zendodo doi, as well as on BioTools (biotools:chemchaste) and SciCrunch (RRID:SCR022208) databases.

Key Points

- Modeling an arbitrary number of spatially diffusing chemicals in a spatial field of cells.
- Ability to account for spatially heterogeneous chemical diffusion coefficients in a spatial field of cells.
- Modeling of both bulk and intracellular biochemical reactions and their coupling in a spatial field of cells.

## Introduction

Understanding the emergent dynamics of spatially heterogeneous cell populations is highly relevant to both eukaryotic and microbial biology. Spatially self-organized biological systems often display nonlinear dynamics [1–3], which may be difficult to mechanistically explain through observation alone, necessitating the use of computational modeling approaches to help guide and explain experimental studies. Several outstanding challenges must be addressed to fully leverage models of spatially organized biological systems [4], not least the development of robust and extensive computational frameworks that allow users to define, explore, and share models in a straightforward manner.

Many computational frameworks already exist for studying the dynamics of spatially organized cell populations. Some of these, such as iDynoMiCs [5], use a bottom-up (discrete, agent-based) approach to modeling individual cell behaviors [6], combined with a top-down (continuum, partial differential equation [PDE]–based) approach to modeling the diffusive transport of nutrients and other chemicals. In this approach, some aspects of cell physiology are “hard-coded,” along with specific “rules” governing their dynamics. In other computational frameworks, the physical forces acting on individual cells are modeled explicitly, but cell physiology is not. In these approaches, cells are treated as extended shapes in space, with cell proliferation and migration implemented through neighborhood update rules, for example, an implementation of the so-called cellular Potts model (e.g., as done in CompuCell3D  [7] and as used in Morpheus  [8]). It is also possible to combine these 2 approaches, into what we call a “hybrid continuum-discrete approach,” where cells are represented by particles, with some aspects of their physiology encoded by rules (e.g., cell division) and others governed by spatially explicit energy or force equations (e.g., cell migration). Such hybrid approaches have been developed by either creating dedicated, new computational frameworks (e.g., HAL [9], PhysiCell [10], Chaste [11]) or adapting existing agent-based [12] or molecular dynamics [13] tools.

Using hybrid modeling tools, cell physiology can theoretically be coupled to the dynamics of chemicals in the bulk medium. This functionality, however, is implemented in a limited fashion in existing platforms. For example, in Chaste, PhysiCell, and CompuCell3D, either only a limited number of bulk chemicals can be dynamically modeled, and/or diffusion coefficients are assumed to be homogeneous. Additionally, the linking of these bulk chemicals to intracellular reactions is limited in terms of number of reactions and couplings that can be encoded in each cell and at the cell–bulk interface. This limits the range of biological phenomena that can be studied within existing computational frameworks.

The coupling between cells and their microenvironment is increasingly being recognized as playing a fundamental role in cell dynamics in the context of both microbial and eukaryotic populations, for example, metabolic environmental feedbacks in the tumor microenvironment [14] and microbial community stability [15]. Additional feedbacks can emerge from cell-excreted enzymes, which introduce reactions in the bulk, and from cell-excreted metabolites or proteins that can affect chemical diffusion coefficients in the bulk or near cells. Such effects arising from the bulk–cell interaction can create their own nonlinear dynamics [16–19] or exert a feedback onto cellular physiology [20–22]. Thus, modeling of metabolic and other feedbacks between bulk environment and cellular behaviors would benefit from the further development of computational frameworks centered on the role of chemical coupling.

To this end, we introduce ChemChaste, a computational framework that allows the simulation of any number of chemical reaction–diffusion systems with or without cells, as well as cell-excreted chemicals or enzymes to react in the bulk phase. ChemChaste builds upon Chaste (https://github.com/Chaste/Chaste) and expands its capabilities with the introduction of (i) unlimited number of PDEs for modeling any number of bulk chemical diffusion dynamics; (ii) heterogeneous diffusion rates, allowing for implementation of different “domains” in the bulk pertaining to different diffusion properties; (iii) expansion of the size of the cellular reaction network that can be implemented to describe cellular behaviors; and (iv) a user interface for defining model structure. The user interface allows cell-internal biochemical reaction systems (cell network ordinary differential equations [ODEs]), spatial reactions in the bulk, and heterogeneous diffusion rates for chemicals in the bulk to be encoded in a file-based system. These features allow easier simulations in ChemChaste, without any need for users to change the C++ source code. Below, we demonstrate the ChemChaste implementation and functionality using a set of chemical and biochemical exemplars, including a cell-based example. All of the source code and user manuals for ChemChaste are provided through GitHub (https://github.com/OSS-Lab/ChemChaste) as an open-source library to accompany Chaste, allowing for its application and further development by the research community.

## Methods

**Figure 1:**
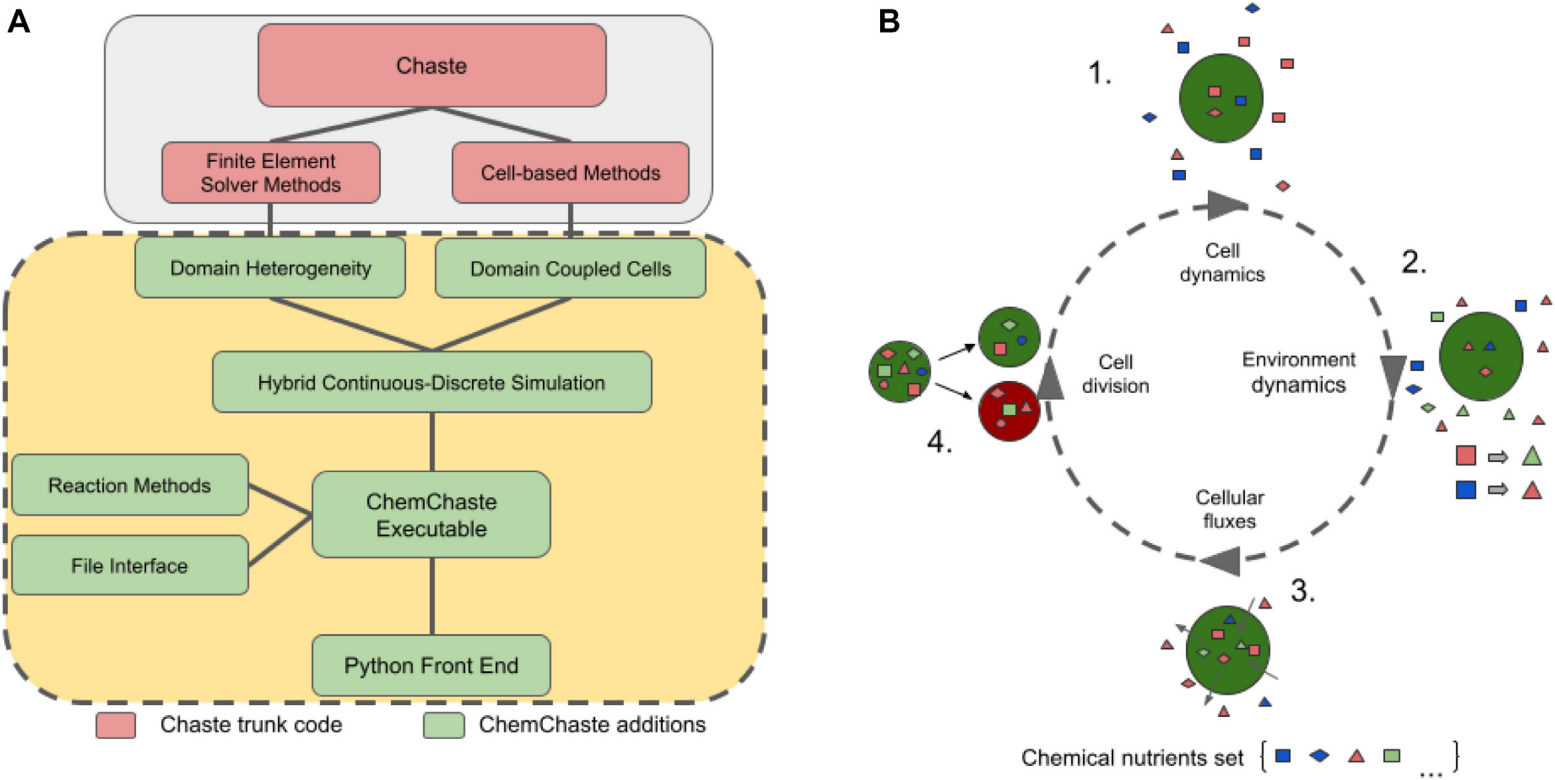
ChemChaste’s simulation framework. (A) ChemChaste classes (in dashed yellow-green) that extend Chaste’s finite element solver capabilities. These build on existing Chaste modules (in solid gray-pink) and allow for heterogeneous spatial domains with varying diffusion rates for chemicals. The cell-based methods are also extended through introducing transport properties linking cell interior and exterior state variables. These extensions are coupled with a file-based user interface allowing higher-level model specification. (B) Four processes that occur over discrete time steps and allow the simulation of cells coupled to the bulk. The cells perform their own system of rules or reactions (cell cycle progression, cell properties, and cellular reaction networks) (1 & 2) before the environmental reaction–diffusion systems are solved (2). The state variables are then coupled through cellular flux through the transport processes and membrane reactions (3), before any implemented cell-based rules (e.g., relating to cell division and/or death) are performed (4).

ChemChaste builds from Chaste, inheriting its adaptable and modular C++ structure [11, 23] and expanding its capabilities with a comprehensive set of C++ classes (Fig. 1). Chaste exhibits many capabilities ideal for the foundation of a hybrid modeling framework, including (i) implementation of a range of on-lattice and off-lattice multicellular modeling approaches in a consistent computational framework [24]; (ii) center-based cell modeling, which treats cells as point particles with radii of interactions [25]; (iii) accounting for cell physiology through empirical rules or a limited intracellular reaction network implemented as a set of ODEs; (iv) modeling of cell physics, including movement and attachment; and (v) modeling of bulk chemical dynamics using PDEs solved numerically using the finite element (FE) method [24]. For specific biological modeling applications, Chaste requires the PDEs and ODEs to be explicitly written by the user as C++ classes, limiting Chaste’s usability to those familiar with C++  [26–28].

Expanding from Chaste, ChemChaste considers parabolic reaction–diffusion systems, where chemicals diffusing and reacting in the bulk are also coupled with cells present in the same bulk, through cellular excretion and uptake. For simulating such cell–bulk coupling, ChemChaste is developed to handle different chemical species confined to the bulk, to cell populations, or present in both phases. ChemChaste also allows for spatially varying chemical diffusion coefficients.

Each ChemChaste simulation features 4 distinct dynamical components that run at each discrete time step of the simulation (Fig. 1B). These involve updating of bulk and cellular chemical systems, their couplings, cell behaviors, and cell positions. The bulk and cellular chemical reaction systems are considered separately: the former is updated by solving reaction–diffusion equations, taking into account any reactions implemented in the bulk, while the latter may in general differ from the bulk chemical system and may involve further chemical species. These 2 systems are coupled through transport of chemicals across the cell membrane. Thus, bulk chemical concentrations are updated according to these couplings. After all chemical concentrations have been updated, any “rules” implemented regarding cell behavior (e.g., division) are checked and subsequent cellular events (e.g., cell death, division) are implemented. Division introduces a daughter cell into the simulation. In this case, the cellular chemicals of the parent cell are redistributed between both cells, based on a user-defined parameter (allowing for symmetric or asymmetric inheritance of cellular chemicals). The location of each cell is updated by numerically integrating its equation of motion. These 2 steps, division and movement, are inherited from Chaste [11]. The user may tailor the simulation details through a file interface system. Further details of the ChemChaste platform are explained below and in the Supplementary Information (SI).

### Expanding the reaction–diffusion system simulations: The Domain Field Class

The core of Chaste is composed of FE solvers and associated spatial meshing routines (see SI, section S1 for details of the FE method as implemented in Chaste). In brief, the FE methods model the bulk domain as a discrete mesh of nodes and approximate the concentration of each chemical across this mesh, subject to a user-defined combination of boundary conditions (BCs): Neumann, Dirichlet, or periodic conditions at the edge of the bulk domain. Over the mesh, Chaste uses a range of ODE solvers, chosen by the user, to determine the ODE solutions at the discrete mesh nodes. Using a set of linear basis functions, these nodal ODE solutions are then interpolated onto a finer grid of points, known as Gauss points, where point-based source terms and diffusive terms are added. Chaste’s FE method then uses the chemical values at the Gauss points to compute the PDE system solutions at the next time step. This implementation has been limited in Chaste to solving the same given ODE for all nodes in the mesh.

Expanding from this implementation, ChemChaste introduces a Domain Field class, which allows us to compute the solution of nodal ODEs generally varying at each mesh node. With the addition of the chemical and reaction classes (see SI, sections S1.3– S1.4), ChemChaste forms a chemical Domain Field wherein the concrete reaction systems are mapped to the FE mesh. This expansion allows for (i) multiple, diffusing bulk chemicals; (ii) reactions among chemicals in the bulk; and (iii) spatially varying diffusion rates for chemicals. With this introduction, the simulation domain may now be broken into subdomains, each containing their own diffusion parameters, ODE systems, and node-based source terms. This allows chemical reaction systems to be confined to subdomains of the simulation for modeling spatial subcompartments with their own diffusion parameters (e.g., a biofilm or tissue surrounded by a bulk). The Domain Field class uses a 2-dimensional (2D) matrix to contain the nodal values that acts as a look-up reference for spatial aspects of the simulation. While this currently limits the ChemChaste simulation to a 2D domain, an extension to 3-dimensional (3D) simulations would be straightforward for a C++-proficient user by editing the source code.

### Coupling the cell physiology and reaction–diffusion system simulations

The core spatial mesh routines of Chaste also form the basis of simulating dynamic cell populations. ChemChaste uses the “node-based” or cell-center modeling approach offered in Chaste [25]. In this approach, a cellular mesh (CM) is defined wherein each mesh node acts as the center of a cell. Each cell is simulated as a particle, and the CM vertices are used to encode any rules (e.g., physical forces) governing physical cell interactions [24, 26]. The CM is also mutable, allowing simulation of cell motility—by defining forces to shift CM nodes—or cell division and death—by performing vertex additions or deletions on the CM [23]. In ChemChaste, cell motility is provided by the passive shunting when new cells are introduced through cell division. Active motility laws are implemented in the Chaste package and can be used by modifying the ChemChaste source code. However, this would bypass the file-based user interface and would not benefit from the ChemChaste features.

ChemChaste expands upon this node-based cell population simulation to introduce the coupling between cellular and bulk chemicals. As explained above, an interpolated Gauss point is produced during the FE simulations. In ChemChaste, this point may also be the location of a cell in the CM where the “volume” of the point-like cell matches the FE mesh point volume share of the environment. When this is the case, membrane and transport reactions are performed on the selected cell, and their outcomes are coupled to the relevant cellular and bulk chemicals. In this way, the cell’s “contribution” to the source term of the related, bulk chemicals’ reaction–diffusion PDE is accounted for. At the same time, the selected cell’s internal chemical concentrations are updated through exchanged chemicals (see SI, section S1.2).

### Specifying chemical reactions and chemicals diffusion properties

ChemChaste allows modeling of 3 different reaction processes based on where they occur: bulk, membrane, and transport reaction. Bulk reactions offer the means to model reactions in the bulk and acting on spatially diffusing chemical species. As explained above, the FE simulations implement on each node of the mesh a reaction rule, which is used to update species’ concentrations accordingly. Bulk reactions occur on these mesh nodes and act as a source/sink term for the PDEs defining the reaction–diffusion system. Membrane and transport reactions involve cellular and bulk chemical species and therefore require knowledge of the concentrations of a given chemical both within the cell object and in the bulk. In the case of membrane reactions, reaction rates depend on both bulk and intracellular chemical concentrations, but there is no chemical species exchange through the membrane. This class of reactions is thus ideal for implementing processes such as membrane-bound enzymatic reactions. Transport reactions implement a chemical flux through the membrane, and internal species may react or exchange with external species.

The 3 reaction types are modeled with user-defined kinetic rate laws, such as mass action or enzymatic kinetics. In ChemChaste, both the stoichiometry and kinetic rates of these reactions are defined through a file-based user interface (see next section and SI, section S2.2.2). Furthermore, bulk reactions can be assigned to a specific subdomain (of the Domain Field) of the mesh. To assist with the assignment of kinetic laws to reactions, ChemChaste implements specific classes describing different kinetic laws. In ChemChaste, chemical species may be provided with a set of properties: name, diffusivity, mass, valence, and Gibbs formation free energy. These properties can be linked to affect the rate of diffusion or rate of a given reaction within which the species participate. Furthermore, when the Domain Field contains subdomains, the domain varying chemicals’ properties may be stored in upstream inheritance classes. This allows simulating changes in diffusivity due to spatial heterogeneities (e.g., bulk media vs. biofilm or tissue). Within the ChemChaste code, these chemical associated parameters can be called by the PDE diffusion functions or reaction systems for the correct subdomain.

### File-based user interface

ChemChaste introduces a file-based interface to enable its use by a wider audience. In particular, ChemChaste has 2 main user-interface systems, one to provide the Domain Field and diffusion properties and one for defining the Reaction System, which together characterize a heterogeneous reaction–diffusion model. The Domain Field files contain the information required to produce the FE mesh and define the labeled subdomains. This file also defines any varying BCs and/or diffusion rates for bulk chemicals. The user supplies a comma separated values (CSV) file of labels denoting the subdomains and a text file of the associated label keys (see SI, section S2.2 for an exemplar Domain Field file). Further CSV files of initial species values, boundary conditions, and diffusion rates on a subdomain basis may also be specified. These files fully characterize the conditions of the simulation space, while the reaction dynamics are detailed in a separate reaction file.

The Reaction System file encodes the bulk, cellular, and coupling (i.e., membrane and transport) reactions as described above. For the bulk reactions, each subdomain can have an associated, separate reaction system file. Another file is used to define the cellular reaction system. Within this cell file, coupling reactions are defined with at most one membrane reaction file and one transport reaction file, each containing a set of reactions of the respective type. All reaction files follow a set format: name of reaction kinetics, chemical equation involving the species, and then the kinetic parameters used by the rate laws (see SI, section S2.2.2). Further rate laws may be implemented by the user, which will then be used in the same way as the supplied rate laws (see SI, sections S4–S6 for details). Overall, the information stored within these files is sufficient to select the desired reaction class, formulate reaction terms, and implement concentration changes when solved within the simulation.

## Results

ChemChaste presents a hybrid continuum-discrete modeling framework for the simulation of individual cells within a chemically active environment. As shown in Fig. 1 and discussed in the Methods section, the framework is composed of an array of different modules building upon each other to fulfill the simulation needs. Here, we verify and demonstrate the functionality of ChemChaste by considering each of these key modules in turn. The accuracy of the PDE solvers was tested through solving the Fisher–Kolmogorov–Petrovsky–Piskunov (Fisher-KPP) equation showing a strong agreement with an analytic series expansion. The simulation of multiple PDEs using the ChemChaste reaction system and file interface system was demonstrated through producing diffusion-driven spatial patterning and temporal oscillations of the Schnakenberg reaction system (Section Modeling multiple, diffusing, and reacting chemicals in ChemChaste). Finally, an exemplar coupled cell simulation was implemented involving a cooperator-cheater system based on enzyme excretion (Section Coupled cell-chemical environment simulations in ChemChaste ).

### Spatial simulation accuracy in ChemChaste: Fisher-KPP equation

To verify and demonstrate the PDE solving capabilities in ChemChaste, a single PDE with a known analytical solution was implemented. The chosen system was the Fisher-KPP equation, which has been used to model the propagation of an invasive species through a population [29, 30] and admits traveling wave solutions with an analytically resolved minimum wave velocity [31]. The corresponding reaction–diffusion equation includes a logistic growth source term,

(1) \begin{eqnarray*}
\frac{\partial U}{\partial t} - D \nabla ^{2} U = rU \left(1-\frac{ U}{\kappa } \right), \end{eqnarray*}

where *U*(**x**, *t*) ≥ 0 is the size of the invasive species population at position **x** = (*x, y*) and time *t*, and the positive parameters *D, r*, and κ denote the diffusion coefficient, linear growth rate, and carrying capacity of the invasive species, respectively. For suitable initial conditions, it is known that this system exhibits pulled traveling wave solutions of the form *U*(*z*), where *z* = *x* − *ct* and *c* ≥ 0 is the wave velocity. It can be shown analytically that the front of these waves travels with a minimum velocity defined by

(2) \begin{eqnarray*}
c_{min} = 2\sqrt{rD}, \end{eqnarray*}

while the observed velocity, *c* ≥ *c_min_*, is dependent on the initial conditions [30, 31].

We implemented the Fisher-KPP equation in a ChemChaste simulation using equation (1) and setting the parameters to unity {*D, r*, κ} = 1. We considered a rectangular bounded domain Ω ∈ [0, 10] × [0, 100] and imposed zero-flux BCs and recorded a 1-dimensional slice across the domain. The simulations were initialized with a strip of invasive species bordering the left boundary of the domain, 0 < *x* < 1:

(3) \begin{eqnarray*}
U(x,y,0) = U_{0} \;\; \text{for} \;\; 0\;\lt\; x\;\lt\; 1, 0\; \lt\; y\; \lt\; 100. \end{eqnarray*}

For equation (1), the minimum wave speed with the selected parameter set is given by *c_min_* = 2.

The FE methods within ChemChaste were used to solve equation (1) subject to the boundary and initial conditions. A traveling wave solution was identified across the 1-dimensional domain slice and compared to the analytical solution of the 1-dimensional Fisher-KPP equation [32], given by

(4) \begin{eqnarray*}
{\rm U(x,y,t)} &=& \frac{1}{1 + \exp (z/c)} \nonumber \\ && + \frac{c^{-2} \exp (z/c)}{(1 + \exp (z/c))^2} \ln \left(\frac{4 \exp (z/c)}{(1 + \exp (z/c))^2} \right) \nonumber \\ && + {\rm O} \left(\frac{1}{c^4} \right) \end{eqnarray*}

where *z* = *x* − *ct* denotes the traveling wave coordinate.

**Figure 2:**
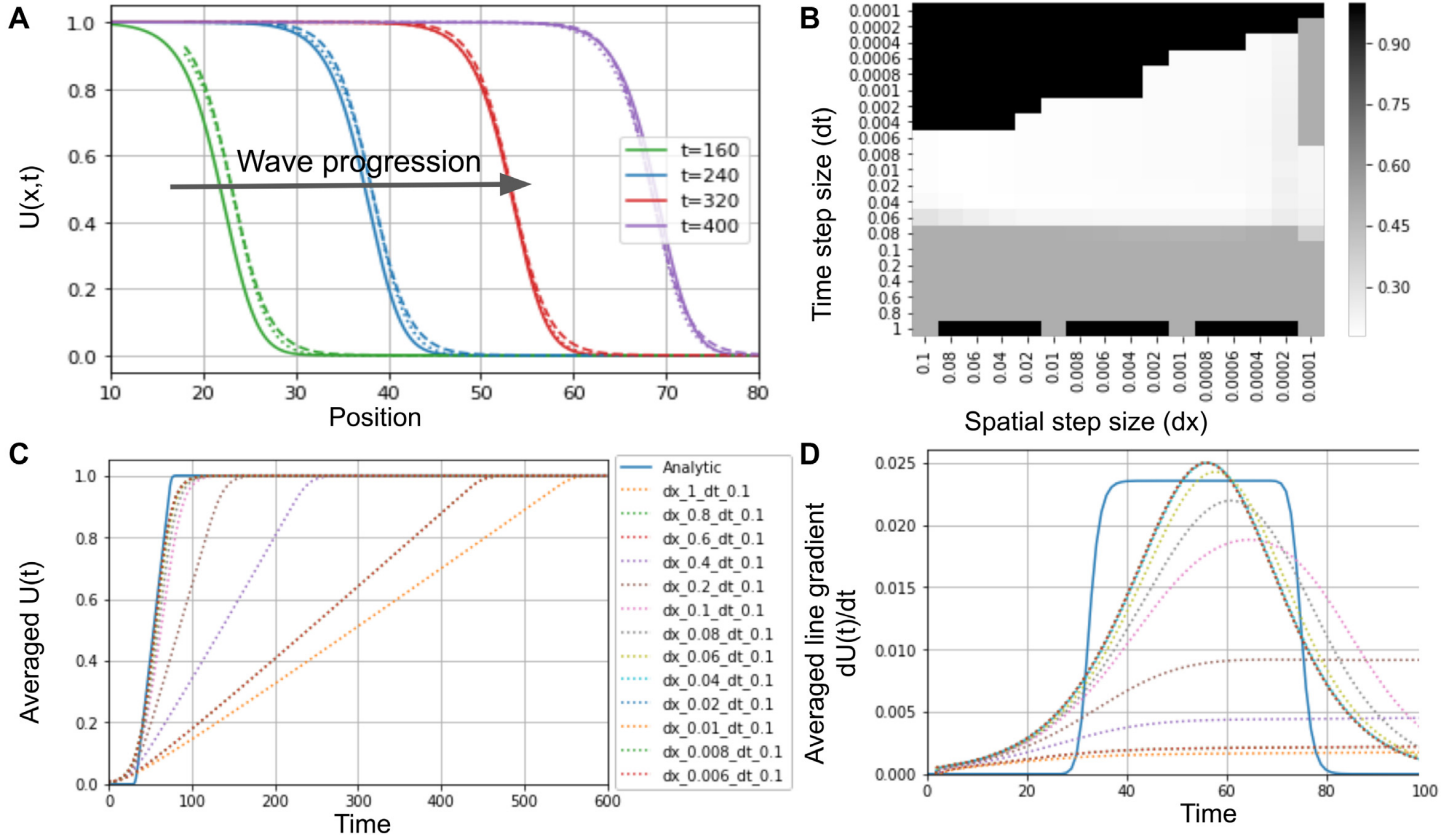
ChemChaste simulations of the Fisher–Kolmogorov–Petrovsky–Piskunov equation. (A) Plot showing the progression of an expanding wavefront through the domain (solid line). The simulation results are accompanied by the analytic solution for the zeroth (dashed line) and first-order (dotted line) expansion in terms of 1/*c*^2^ in equation (4). The wave speed in simulation is initially faster than the analytical minimum wave speed *c_min_* = 2, calculated with equation (2), but with agreement at later times implying the correct asymptotic wave velocity has been reached. (B) Heatmap of *L*^2^ convergence scores for simulations using a range of spatial and temporal step sizes. The simulations for given step sizes are compared to the analytically determined value, with the lower scores suggesting closer values. A threshold was used reducing higher scores to 0.5 (gray pixels). This includes simulations whose numerics diverged. A second source of ill convergence occurs when the linear algebra routines fail to complete within in-built tolerance ranges. These areas are represented by an elevated score or 1.0 (black pixels). (C) Traces for the solutions *U*(*t*) averaged across the domain for different spatial and temporal step sizes. The traces converge to the analytical solution with decreasing step size. (D) The gradients of the slopes in plot (C) sharing the same legend. The gradients are suggestive of the velocity of the wave passing through the domain.

The results were visualized using ParaView [33]. Two tests were considered: comparing the traveling wave front solution produced by the ChemChaste simulation versus the analytic form given by equation (4), and comparing the simulations’ convergence stability under decreasing temporal and spatial step size. Results for both tests are given in Fig. 2 and show a good agreement between the ChemChaste simulation output and expected results determined through analytic solutions. Additionally, the convergence with decreasing temporal and spatial step sizes suggests stable numerics albeit with the waves showing longer accelerating phases than the expected analytic top-hat gradient. Therefore, the ChemChaste implementation was able to correctly simulate dynamics (in this case, the traveling wave phenomenon) in simple PDE with stable and accurate numerics.

### Modeling multiple, diffusing, and reacting chemicals in ChemChaste: Schnakenberg reaction–diffusion system

ChemChaste builds upon Chaste’s PDE solvers to enable the simulation of multiple PDEs over the domain. While Chaste is restricted to solving 3 PDEs, ChemChaste’s limiting factor is solely the available computational resources. To test the multidimensional PDE simulation, and to verify the file interface system, we implemented the well-studied 2-species reaction system commonly known as the Schnakenberg system [34, 35] and shown in equations (5) to (7). When these reactions are modeled with mass action kinetics, they are shown to display temporal oscillations and diffusion-driven spatial patterning for distinct, defined parameter regimes  [36, 37]. These phenomena were reproduced here using ChemChaste.

The Schnakenberg reaction system involves 2 chemical species *U, V* that are produced, interconverted, and removed via the reactions

(5) \begin{eqnarray*}
\emptyset\; {\mathop{\rightleftharpoons}^{{{k}_{1}}}_{{{k}_{-1}}}}\; U, \end{eqnarray*}

 

(6) \begin{eqnarray*}
\emptyset\; {\mathop{-\!\!\!-\!\!\!-\!\!\!\longrightarrow}^{k_{2}}}\; V, \end{eqnarray*}

 

(7) \begin{eqnarray*}
2 U + V\; {\mathop{-\!\!\!-\!\!\!-\!\!\!\longrightarrow}^{k_{3}}}\; 3 U , \end{eqnarray*}

where the reaction rate constants are denoted by *k*_1_, *k*_−1_, *k*_2_, and *k*_3_. Applying mass action kinetics to these reactions yields the reaction ODEs

(8) \begin{eqnarray*}
\frac{dU}{dt} & = R_{U}(U,V) = k_{1} - k_{-1} U + k_{3} VU^2, \end{eqnarray*}

 

(9) \begin{eqnarray*}
\frac{dV}{dt} & = R_{V}(U,V) = k_{2} - k_{3}VU^2, \end{eqnarray*}

where the reaction rates *R_U_*, *R_V_* describe the change of each species’ concentration in a given time step and also provide the source terms to the reaction–diffusion PDEs. The PDEs are satisfied across the whole 2D domain space, Ω, and are given by

(10) \begin{eqnarray*}
\frac{\partial U}{\partial t} - D_{U} \nabla ^{2} U = R_{U}(U,V), \end{eqnarray*}

 

(11) \begin{eqnarray*}
\frac{\partial V}{\partial t} - D_{V} \nabla ^{2} V = R_{V}(U,V), \end{eqnarray*}

where *D_U_*, *D_V_* are the spatially homogeneous isotropic diffusion coefficients. Here, we consider a square bounded domain Ω ∈ [0, 100] × [0, 100], which are subject to zero-flux Neumann BCs

(12) \begin{eqnarray*}
\mathbf {n} \cdot \nabla U = \mathbf {n} \cdot \nabla V = 0 \quad \text{on} \quad \partial \Omega . \end{eqnarray*}

Each simulation begins with the randomly perturbed initial conditions defined on each node of the FE mesh,

(13) \begin{eqnarray*}
U(x,y,0) & = U_{0} + \xi , \end{eqnarray*}

 

(14) \begin{eqnarray*}
V(x,y,0) & = V_{0} + \zeta , \end{eqnarray*}

where ξ, ζ ∼ *Uniform*( − 1, 1) are uniformly distributed random fields bounded by the interval [ − 1, 1].

Two parameter sets were considered: one for temporal oscillations and one for diffusion-driven patterning [36]. Temporal oscillations are present when the homogeneous system, equations (8) to (9), display limit cycle behavior. Spatial patterning across the domain occurs when the spatially uniform steady-state solution to equations (10) to (11) is linearly stable in the absence of diffusion (*D_U_* = *D_V_* = 0), but linearly unstable in the presence of diffusion. The resultant spatial patterning in the 2D concentration maps is referred to as displaying diffusion-driven instabilities or Turing instabilities [37–40]. These dynamical cases were found to occur for specific parameter sets, as listed in Table 1.

**Table 1:** Parameters used in the Schnakenberg reaction simulation, with the values selected based on analytical solutions of this system and to demonstrate the possible oscillatory and patterning dynamics

Case	*k*_1_	*k*_−1_	*k*_2_	*k*_3_	*D_U_*	*D_V_*	*U*_0_	*V*_0_
Fig. 3A	0.5	2.2	1.5	1.0	0.5	0.5	0.91	1.67
Fig. 3B	0.1	1.0	0.9	1.0	1	40	1.0	1.0

These parameters were determined through considering small linear perturbations for conditions that provided the expected phenomena in the 2 cases, equations (8) to (9) and (10) to (11), and selecting parameter sets that satisfy the algebraic equations [37] (see SI, section S3 for details). The values *U*_0_, *V*_0_ were used as the initial conditions for the 2 cases.

**Figure 3:**
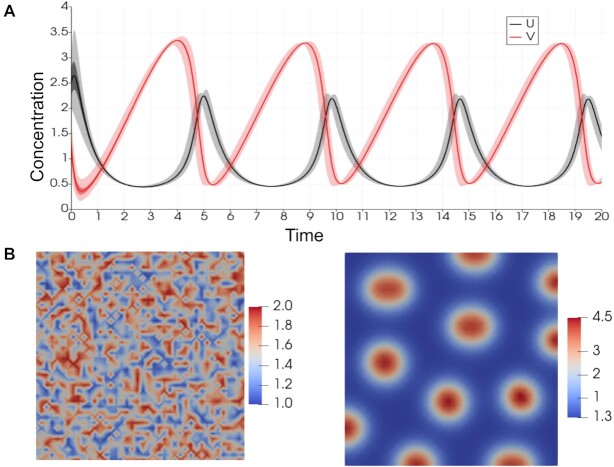
The Schnakenberg reaction system showing the oscillatory and patterning dynamics. (A) The 2 curves show the concentration of *U* and *V* averaged over the nodes in the domain for each time step from the simulations run using oscillatory regime parameters. The concentration traces show the range (light), quartiles (darker), and the spatially averaged concentration (dark) for both chemicals. (B) Domain maps of the initial and final (i.e., steady-state) distribution of *U* and *V* in simulations using parameters for the patterning dynamics (see Table 1). The initial distribution is formed by the addition of uniform random noise at each node point (see equations (13) and (14)).

We have verified, using ChemChaste, that this model exhibits the expected spatiotemporal dynamics for the tested parameter regimes (see Fig. 3). These results are as expected for the parameters used, based on analysis of equations (8) to (9) and (10) to (11). Therefore, these tests verify that ChemChaste was able to both correctly parse the chemical reaction files and simulate multichemical reaction–diffusion systems capable of complex dynamics and patterning.

### Coupled cell-chemical environment simulations in ChemChaste

A main motivation behind developing ChemChaste was to simulate a hybrid continuum-discrete model of cells within a chemically reactive environment, where bulk and cell-secreted chemicals and other entities such as proteins can diffuse as well as react. This is a common biological scenario, as seen, for example, in the case of microbial utilization of cellulose or other complex resources, which must be treated by enzymes before a cell can metabolize or uptake them [41]. The core aspects of this scenario (i.e., a cell-secreted enzyme mediating a reaction in the bulk) are also found in cases outside of substrate uptake, for example, in detoxification of the environment [42]. In ChemChaste, this scenario is readily modeled through implementation of bulk reactions and coupling of cellular metabolic reactions and environmental PDEs.

**Figure 4:**
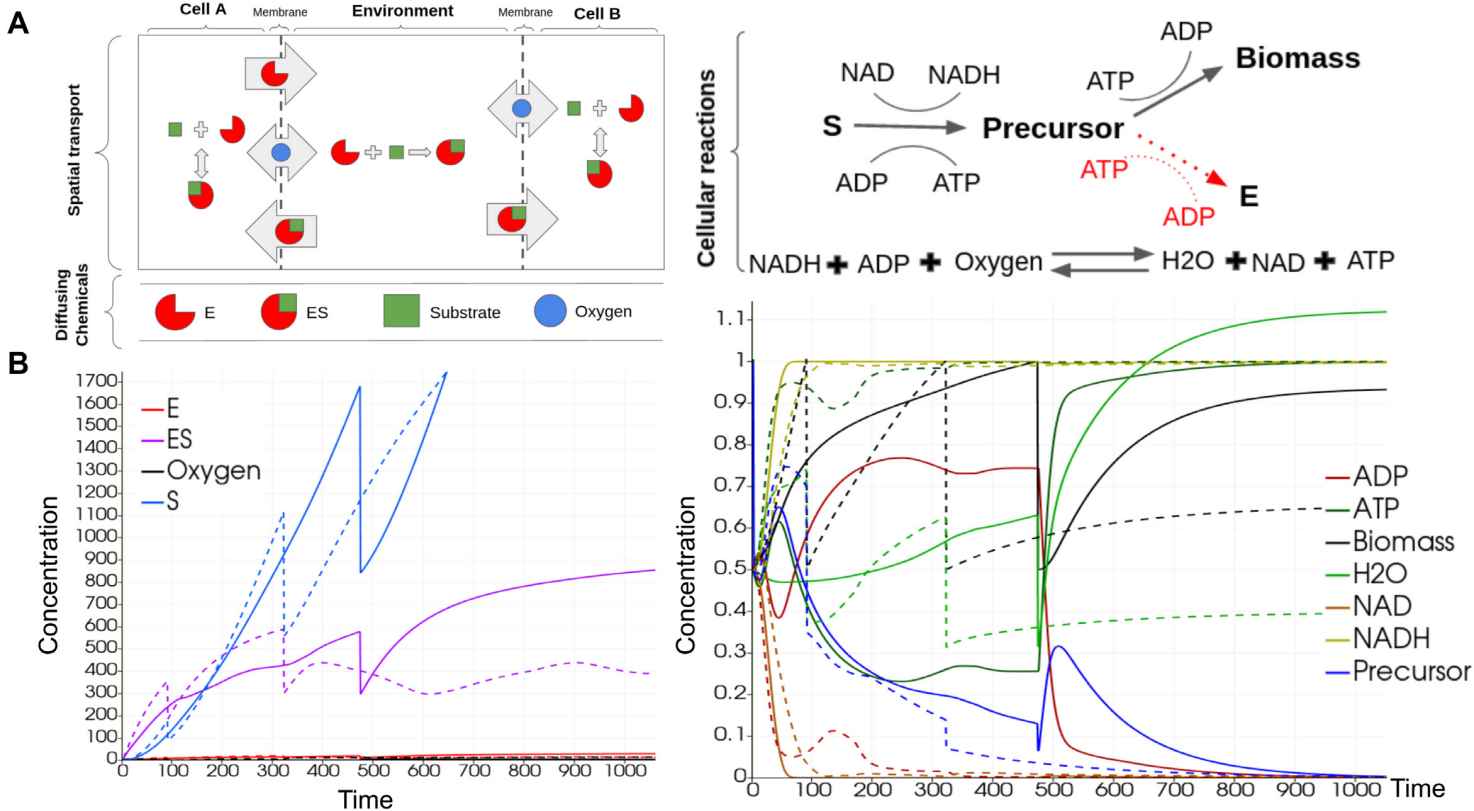
The simulation schematic and results for the exemplar cellular model with cell–environment coupling. (A) Cartoon showing the 2 cell types and cellular reaction system implemented in the simulations. One cell type, the “cooperator,” excretes an enzyme that can bind an environmental substrate, while the other—the “cheater”—does not produce the enzyme (left). Both cell types can take up the enzyme–substrate complex and process it through a series of internal reactions (right). Note that the enzyme-producing pathway is only active in the cooperator cells, which has to invest substrate between this pathway and the biomass-producing pathway. (B) The concentrations for each chemical within the cell are displayed over time for a cell of both types: cooperator (solid) and cheater (dashed) lines. The main plot (left) shows the concentrations of ES and S (chemicals harvested from the environment). The inset (right) shows the concentrations of the cell-internal chemicals. Sharp changes in cellular concentrations are due to cell division and sharing of chemicals between the parent and offspring.

Here, we provide a simplistic toy example for illustrative purposes and for testing ChemChaste implementation of cellular reactions and cell–environment coupling. More detailed and realistic simulations can be readily constructed by users, through a developed ChemChaste user interface. For the exemplar test case, we modeled a growing cell population harboring 2 cell types, along with a chemical resource (i.e., substrate) that is not readily taken up. One cell type—termed cooperator—excretes an enzyme that can allow the internalization of the substrate, while the other cell type—termed cheater—does not excrete the enzyme but can also internalize the enzyme-bound substrate (Fig. 4A). The cells process the internalized substrate to produce a pseudo-chemical species (called “biomass”), which is used as a proxy for monitoring cell growth. Once the cellular biomass concentration reaches a threshold value, the cell divides into two, the parent and offspring, sharing the internal concentrations equally between both parent and offspring cell. The offspring cell is placed at a random neighboring location around the parent cell and the population undergoes positional updating to accommodate the new cell.

Previous agent-based simulations of growing cell populations harboring cheater and cooperator types have found spatial segregation of cell types within the population [43–45]. This cell sorting is linked to the disparity in growth rates of the 2 species, which may be due to substrate availability and dependency, and is of interest in game-theoretic investigations of mutual interactions in biofilms [46, 47]. The presented simulations are conceptually similar to these previous studies but differ in their mechanistic implementation of substrate scavenging, as a cooperative trait, as well as the inclusion of both substrate and oxygen diffusion in the bulk.

In the presented model, the 2 types of cells were introduced into the simulation domain, which contains 2 chemicals that diffuse in the bulk: oxygen (*O*_2_) and a substrate, *S*. Furthermore, the cells excrete and take up a scavenging enzyme, *E*, the enzyme–substrate complex, *ES*, and *O*_2_, which freely diffuses in the bulk. To capture dynamics of cell growth, a simple metabolic network is implemented in each cell, defined by the following toy reactions that abstract biomass generation and the main respiratory and fermentative metabolic pathways:

\begin{eqnarray*}
&& ES {\mathop{-\!\!\!-\!\!\!\longrightarrow}^{k_{1}}} E + S, \nonumber\\ && S + {\rm NAD^+ + ADP} {\mathop{-\!\!\!-\!\!\!\longrightarrow}^{k_{2}}} {\rm Precursor + NADH + ATP}, \end{eqnarray*}

 

\begin{eqnarray*}
{\rm NADH + ADP + O_2\; {\mathop{\rightleftharpoons}^{{k}_{3}}_{{k}_{-3}}} NAD^+ + H_{2}O + ATP}, \end{eqnarray*}

 

\begin{eqnarray*}
{\rm Precursor + ATP} {\mathop{-\!\!\!-\!\!\!-\!\!\!\longrightarrow}^{k_{4}}} {\rm Biomass + ADP}, \nonumber\\ {\rm Precursor + ATP} {\mathop{-\!\!\!-\!\!\!-\!\!\!\longrightarrow}^{k_{5}}} {\rm E + ADP}, \end{eqnarray*}

where NAD^+^, NADH, ADP, and ATP are the usual energy and electron carrier molecules internal to the cell. This toy reaction set captures substrate uptake (reaction 1), recycling of NAD^+^/NADH and ADP/ATP pairs through fermentative and respiratory pathways (reactions 2 and 3), and biomass and scavenging enzyme production through ATP investment (reactions 4 and 5). For the simulations, these reactions are modeled with mass action kinetics with shown reaction rate constants. All reaction rate constants are set to 1 in both cell types, except for *k*_5_, which is set to zero in the cheater cell type. The overall simulation schematic for this cellular system is shown in Fig. 4.

In addition to the cellular reaction network, we implemented bulk reactions for the enzyme binding to the substrate in the extracellular media, the enzyme being degraded in the bulk, and the diffusion of the substrate (*S*), enzyme (*E*), and the enzyme–substrate (*ES*) complex.

\begin{eqnarray*}
{ES} {\mathop{\rightleftharpoons}^{{k}_{1}}_{{k}_{-1}}{E + S}}, \end{eqnarray*}

 

\begin{eqnarray*}
E\; {\mathop{-\!\!\!-\!\!\!-\!\!\!\longrightarrow}^{k_{2}}} \emptyset. \end{eqnarray*}

**Figure 5:**
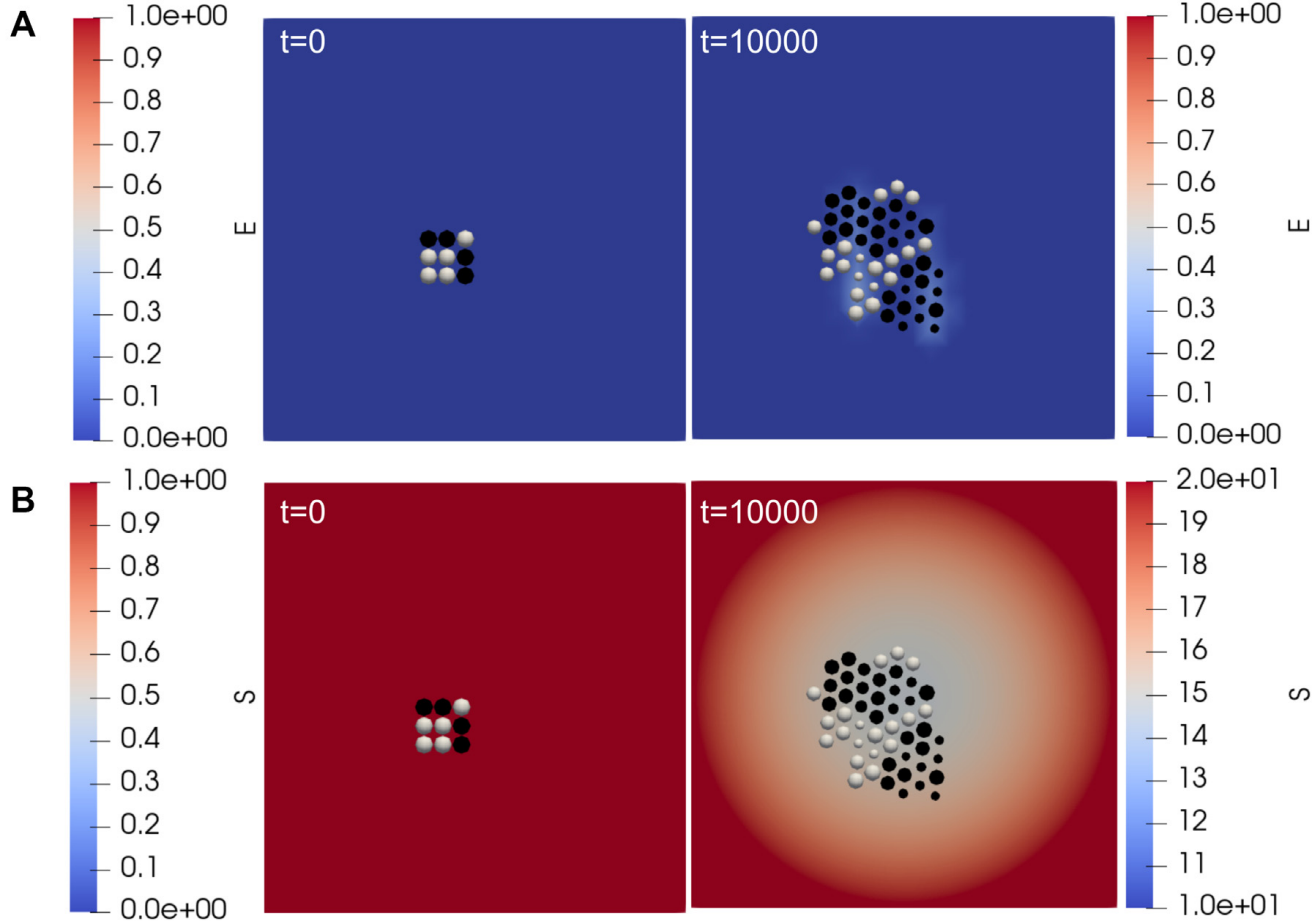
Domain maps showing the cells and the spatial concentration of the enzyme and nutrient species, *E* and *S*, in the bulk at the beginning, *t* = 0, and at the end, *t* = 10,000, of the simulation. The size of the cells demonstrates the accumulation of “Biomass,” with larger cells being closer to dividing. The cooperator and cheater cells are colored white and black, respectively. The upper row (A) shows simulation results with the enzyme (*E*) concentration plotted across the domain. The lower row (B) shows simulation results for the substrate (*S*) across the domain with the replenishment of the substrate at the boundary.

The parameters for these reactions were scaled for computational efficiency and are given in SI, section S2.3. We performed simulations through the hybrid continuum-discrete solvers introduced in ChemChaste. A reaction–diffusion PDE was solved over the domain for the diffusing species {*E, S, ES, O*_2_} with Neumann BCs at the domain boundary. The Neumann boundary conditions allow continual replenishment of substrate to drive the system. The cells were placed in the center of this domain with a single cell of each type and allowed to grow over the simulation course, as shown in Fig. 5. The chemical concentrations in each cell and the bulk were recorded over the simulation. Note that initial substrate levels at the beginning of the simulation are low but will linearly increase due to the implementation of the Neumann boundary conditions. Additional boundary conditions, like Dirichlet type, can be defined per the user files.

We show the dynamics of cellular and bulk chemicals in Figs. 4 and 5. While Fig. 4 is focused on the cell concentrations, Fig. 5 demonstrates the impact that the cells have on local chemical concentrations. In Fig. 5A, we see higher enzyme concentrations in the vicinity of cooperator cells. This is as expected, since these are the cells excreting the enzyme. We expect that such higher local concentrations of enzyme will be enhanced with lower enzyme diffusion rates and enzyme degradation rate in the bulk. In Fig. 5B, we see the substrate concentration, with higher values at the domain edge (due to influx of substrate) and lower values near the cell population (due to cellular uptake). Evaluating Figs. 4 and 5, together, we see a greater uptake of the substrate by the cooperator cells and a greater rate of cell biomass increase, compared to the cheater cells. Thus, the localized pockets of high enzyme concentrations around cooperator cells can lead to their growth rate surpassing that of cheaters and subsequently lead to a spatial segregation of the 2 cell types. While further simulations with different parameter sets are needed to fully confirm these dynamics, the presented results provide an exemplar implementation of cellular simulations in ChemChaste and confirm expected cooperator–cheater dynamics.

We conclude that the presented toy model and exemplar implementation of a cellular simulation demonstrate ChemChaste’s flexibility and capabilities in developing models featuring cell–environment coupling along with environmental reaction–diffusion.

## Conclusion

We have presented ChemChaste, a computational framework for hybrid continuum-discrete modeling of multicellular populations coupled to chemical reaction–diffusion systems. In contrast to existing computational frameworks, ChemChaste facilitates chemical couplings between bulk and cellular metabolic processes through an arbitrary number of diffusing chemicals that can undergo chemical reactions in the bulk and that can have spatially heterogeneous diffusion coefficients. ChemChaste simulations are implemented using a simple file-based interface and can be used to implement different biological and chemical scenarios for modeling complex cell–environment chemical coupling and resulting emergent phenomena.

We have presented several exemplar simulations in ChemChaste, which produce the expected dynamical behaviors in given parameter regimes. These exemplars were specifically chosen to demonstrate ChemChaste’s functionality and flexibility, instead of presenting an exhaustive list of the possible phenomena that may be investigated using this tool. Applications of immediate interest can include different observed cases involving coupling between cellular physiology, cell excretions, and environmentally diffusing reactions such as metabolic switching of cell types coupled to a reactive environment [15, 48]; coupled chemical reactions in the bulk and within cells [38]; coupling between cell secreted enzymes, signaling, and motility [49]; and cell-chemical systems presenting spatially varying diffusion coefficients (e.g., within and outside of a tissue) [20]. In the current release, cells are represented by point-like agents that are effective models for disperse microbial systems where the size of a cell is in the micro- to submicron range. Therefore, juxtacrine transport is not implemented, and the change in the concentration gradient over the cell is negligible. Future versions of ChemChaste may look to relaxing the cell size constraint to provide a more appropriate model for larger or filamentous cells seen in mammalian or fungi systems.

Some of these investigations may require further expansion of ChemChaste. In particular, while the underlying Chaste code is already capable of implementing 3D simulations, some modifications to the model input system and parsing routines would be required to enable ChemChaste to be used for such simulations. However, for users proficient in C++, the addition of new classes is straightforward through the addition of new user-defined classes to the ChemChaste C++ class hierarchy using the modular structure of the framework. In this way, we hope ChemChaste will prove a useful tool for investigating the chemical mechanisms behind a range of phenomena in spatially organized biological systems.

## Availability of Source Code and Requirements

Project name: ChemChaste

Project home page: https://github.com/OSS-Lab/ChemChaste [50]

Operating system(s): Platform independent

Programming language: C++, Python

Other requirements: Docker

License: BSD 3-Clause License

Any restrictions to use by nonacademics: license needed

RRID: SCR022208

## Availability of Supporting Data

Snapshots of our code and other data further supporting this work are openly available in the GigaScience repository GigaDB [51].

### Additional Files

**Supplementary Fig. S1**. Cartoon showing the interpolation procedure from finite triangle elements to Gauss point and coupling to cell agents. (A) The nodes (blue) of the triangle are located at positions $\tilde{n}_i$ and each node contributes to the particular Gauss point (red square) through the node’s basis function ϕ_*i*_ for node *i* ∈ {*x, y, z*}. The node locations and other interpolated quantities are mapped from the local reference triangle to the global mesh, that is, by applying a mapping function to the node position $M(\tilde{n}_i) \rightarrow n_i$, which may, in general, rotate and stretch the reference triangle. (B) Cells have a point location that shares the location of a Gauss point. Each point in Ω is associated with a spatially dependent reaction system, $R(\mathbf {x}, U, t)$, and may also be associated with a cell. These cells (green) contain their own reaction system, $R_{Cell,p}(\mathbf {u}_p,\mathbf {x}_p,t;\mathbf {k}_p)$, and are coupled to the domain through a transport law $T_p(U,t;\mathbf {k}_p)$.

**Supplementary Fig. S2**. The inheritance structure for the bulk domain reaction files currently implemented in ChemChaste. These reactions are shown in Supplementary Table S1. The structure builds from the base reactions, ZerothOrderReaction, to add more complex reaction rate laws.

**Supplementary Fig. S3**. The inheritance structure for the transport (A) and membrane (B) reaction types currently implemented in ChemChaste. These reactions are shown in Supplementary Table S2 and Supplementary Table S3, respectively. (A) The transport reactions build upon ZerothOrderTransportIntoCell as a base while in (B), the membrane reactions build upon ZerothOrderCoupledMembrane. These laws may be built upon to add more complex transport and membrane reaction rates. The user may write their own transport processes and membrane rate laws by following the file structure given in sections S4–S5.

**Supplementary Fig. S4**. Example “run-script” file for defining features of ChemChaste simulations. The structure of this “run-script” file is such that it is divided into sections, as shown with a letter-based labeling on the figure and as explained next. (A) This section defines the import files used for running parallel simulations and controlling the simulation compilation and implementation. (B) This section defines the configuration files for each user simulation that are contained within the *DataInput* directory of ChemChaste. (C) This section defines the global simulation parameters, in particular those that are used for parameter sweeping. The simulation ID created in this section is shared across a set of simulations to be run in parallel. For these simulations, a different parameter value is to be used—in this example, the “time step size” parameter. (D) This section defines the command to be used in the command-line initiation of a simulation. The command is created in a series of steps, by appending different aspects of the simulation command together. First the simulation executable, in this case the cross-feeding simulation, is created and then appended the simulation type. Then, the desired simulation parameters are amended to the command. In this case, note that the simulation time step is set by grabbing its value from the provided parameter list and by making use of a for loop structure. Finally, the destination for the data file is appended to the command using the parameter sampling_rate and a value of 1*e*^−1^. (E) This section is a repeat of section (D) but using a reaction-only simulation example. The Schnackenberg simulation is used with the same parameters as in (D) but where simulation_type is set to domain_only. (F) This section defines final aspects of simulations, such as number of processor cores. The parallel simulations are mapped to count number of processor cores.

**Supplementary Fig. S5**. The configuration file (A) and overall directory structure (B) for simulating a reaction–diffusion system. (A) The configuration file containing the basic simulation parameters: output directory, simulation end time, number of chemical PDEs to simulate, the domain, and FE element dimensions. The configuration file also contains the directory paths and names of the different files used in the simulation. The file structure used during the domain-only simulation (B). The file paths are defined within the configuration file and follow CSV file types for parameters and defining the domain while TXT files are used for writing the reactions.

**Supplementary Fig. S6**. Files defining the domain topology and providing simulation parameters. (A) CSV file defining the domain topology. The nodes on the domain are labeled according to this matrix where subdomains are specified by using different labels. The size of the matrix specified in this file is directly proportional to the size of the simulation mesh after scaling. Therefore, a rectangular domain specified in this file will produce a rectangular domain mesh. (B) Boundary conditions are implemented on the outside nodes of the FE mesh. The conditions are specified as “state variable,” “BC type,” and “BC value.” (C) Domain key file, which connects the “simple” labels used in the domain topology file with the domain names. (D) Diffusion in the simulations is modeled as isotropic diffusion where the coefficient value is the same in all directions. These values for each state variable are provided for all subdomains names in (C). (E) The initial conditions in the domain are defined for each state variable (chemical) on each subdomain. The value can differ for each subdomain, and the user can specify whether to perturb the value by a uniform random value.

**Supplementary Fig. S7**. The implementation of bulk reactions in user-defined files. (A) A CSV file defining the numeric ID of reaction files, which describe a series of chemical reactions. The reactions defined in a file associated with a node will determine those reactions to be active on that node. This allows for the creation of reaction subdomains, which are not necessarily the same as the diffusion subdomains. (B) A reaction key file that connects the numeric IDs used in part (A) to actual reaction file names. These names refer to TXT files containing the reaction system that are to occur on the associated nodes (creating the reaction subdomain). (C) An example reaction file, SchnackenbergReactionFile.txt. Each line denotes a separate reaction. Reactions are defined using a standard form composed of “rate law,” “:” rate delimiter, reaction equation, “;” reaction delimiter, and the rate law parameters.

**Supplementary Fig. S8**. The configuration file for the cell-coupled simulations. This configuration file defines the directory paths to associated files and includes some parameters specific to cell-coupled simulation. The files, accessible via the defined directory paths, define the structure and subpopulations of cells; the information is stored in the files cell_file_root, cell_file, cell_key_file. See example given in Supplementary Fig. S10. Simulation parameters used to couple the cell and domain mesh are also provided. ''cell_mesh_origin'' denotes the origin of the cell population structure with respect to the domain mesh. ''linear_force_cutoff'' is used as an interaction strength parameter for the Hookean linear spring force that connects the cells in the simulation.

**Supplementary Fig. S9**. Description of directories and cell properties files for the cell-coupled simulations. (A) The directory structure for a cell-coupled simulation. Files relating to the domain structure, as detailed in Supplementary Fig. S9, are contained within theDomainField directory. The cell files are provided within the Cells directory. Each cell type is provided its own subdirectory, with the same name as the label name in the CellLayerKey.csv, Supplementary Fig. S10D. For each cell type, we define an initial concentrations file (B) and a species threshold file (C). (B) The initial conditions are provided for each cellular state variable (chemical) using the following form: name, value, and whether to perturb the initial value on a nodal basis. (C) The threshold values for each cellular state variable (chemical) are provided in the following order: name, maximum value, and minimum value.

**Supplementary Fig. S10**. The files associated needed to form the cell mesh and populate the cells with chemical reactions. These files are to be placed in the cell subdirectory of the ChemChaste main directory (see Supplementary Fig. S9A). (A) The ''CellLayerTopology.csv'' file provides the information for the initial cell mesh topology. It is written in the same format as the domain layer, Supplementary Fig. S6a. In this example, a rectangular mesh of 2 cells is defined where the first cell is labeled ''1'' and the second labeled ''2''. This mesh is aligned with the domain mesh through translating the origin of the cell mesh as specified by the cell_mesh_origin parameter in the configuration file (see Supplementary Fig. S8). (B) The ''CellLayerKey.csv'' file contains the key mappings from the numeric ID label used in ''CellLayerTopoogy.csv'' to the cell type used for the directory names. In this example, 2 cell types are used: *CellA, CellB*. The cellular reactions for *CellA* are provided in 3 TXT files: (C) the internal reactions, (D) the transport reactions, and (E) the membrane reactions. (C) The ''Srn.txt'' file contains the cellular reaction system. Each line contains one reaction. The reactions are written in the standard form: reaction kinetic law, “:” reaction law delimiter, reaction chemical equation, “;” parameter delimiter, and kinetic law parameters. (D) The file ''TransportReactions.txt'' lists the reactions/processes that couple the cells to the external domain. The reactions in this file are written in such a way that the chemicals on the left-hand side represent the species in the bulk domain, while those on the right-hand side represent the species inside the cell. Otherwise, they follow the form in (C) but using an appropriate set of reaction rate laws. (E) The file ''MembraneReactions.txt'' lists the reactions that are coupled at the cell membrane and with reaction occurring on the outside of the cell and one on the inside of the cell. These reactions are separated by the membrane delimiter, “|,” when written and the reaction rate law belongs to the membrane reaction set.

**Supplementary Fig. S11**. (A) The bifurcation diagram for the Schnakenberg reaction system where reaction rate *k*_1_ is used as the bifurcation parameter and displaying the fixed points for the variable *U*. The diagram displays a stable steady state where spatial patterning may be found ending in a Hopf bifurcation point (*k*_1_ = 0.61, *U* = 0.96) with oscillations beyond this. (B) The period of the oscillations for varying *k*_1_ starting at the Hopf bifurcation point. The marked point indicates the *k*_1_ value used to produce the oscillations in Fig. 3A of the main manuscript. The subsequent period of oscillations corroborates the ChemChaste output.

**Supplementary Table S1**. Reaction rates currently implemented in ChemChaste. The rates include constants *k_f_*, *k_r_*, *k_cat_*, *K_M_* and labels for spectator chemicals. For the reversible reactions, the rates may be negative $R(\mathbf {u}) \in \mathbb {R}$, implying the reaction occurs in the reverse direction, while in the irreversible reaction, the rate must be positive $R(\mathbf {u}) \in \mathbb {R}_{\ge 0}$ The user may implement their own reactions rates, building upon these forms by adding a new reaction file to the inheritance structure of ChemChaste (section S3).

**Supplementary Table S2**.The foundational transport process types implemented at present, including whether a process is reversible and the functional rate law used. The user may implement their own laws by adding a new transport reaction file to the ChemChaste system (section S4).

**Supplementary Table S3**. The membrane reaction types implemented at present, including whether a reaction is reversible and the functional rate law used. The user may implement their own membrane reaction laws by adding a new reaction file to the ChemChaste system (section S5).

**Supplementary Table S4**. ChemChaste simulation parameters that can be set via the “run-script” file. These parameters control the type of simulation to run, the solver properties such as end time and solver time step, and finite element properties such as spatial domain dimensions and element dimensions for the FE implementation. Each parameter is set by name in the simulation configuration file; an example is provided in Supplementary Fig. S5a.

## Conflict of Interest

None declared.

## Funding

This work was supported by the UK’s Biotechnology and Biological Sciences Research Council [BB/T010150/1 to O.S.S., BB/R016925/1 to A.G.F.] and the UK’s Engineering and Physical Sciences Research Council and Medical Research Council [EP/L015374/1 to University of Warwick’s Mathematics of Real World Systems Centre for Doctoral Training]. O.S.S. acknowledges additional funding from the Gordon and Betty Moore Foundation (Grant GBMF9200, https://doi.org/10.37807/GBMF9200).

## Abbreviations

PDE: Partial differential equation, ODE: Ordinary differential equation, SI: Supplementary information. FE: Finite element, CM: Cellular mesh, BC: Boundary condition, CSV: Comma separated values, KPP: Kolmogorov–Petrovsky–Piskunov, ES: Enzyme-substrate

## Supplementary Material

giac051_GIGA-D-21-00383_Original_Submission

giac051_GIGA-D-21-00383_Revision_1

giac051_GIGA-D-21-00383_Revision_2

giac051_Response_to_Reviewer_Comments_Original_Submission

giac051_Response_to_Reviewer_Comments_Revision_1

giac051_Reviewer_1_Report_Original_Submission Cheryl Sershen -- 1/14/2022 Reviewed

giac051_Reviewer_2_Report_Original_Submission Lutz Brusch -- 1/20/2022 Reviewed

giac051_Reviewer_2_Report_Revision_1 Lutz Brusch -- 4/7/2022 Reviewed

giac051_Supplemental_File
