# Supplementary material for: ChemChaste: Simulating spatially inhomogeneous biochemical reaction–diffusion systems for modeling cell–environment feedbacks
Source: Gigascience. 2022 Jun 17;11:giac051. doi: 10.1093/gigascience/giac051 (PMC9205757; doi:10.1093/gigascience/giac051)
Supplement: giac051_GIGA-D-21-00383_Revision_2 [file giac051_giga-d-21-00383_revision_2.pdf]

GigaScience

ChemChaste: Simulating spatially inhomogenous biochemical reaction-diffusion systems for modelling cell-environment feedbacks

--Manuscript Draft--

**Manuscript Number:**	GIGA-D-21-00383R2
**Full Title:**	ChemChaste: Simulating spatially inhomogenous biochemical reaction-diffusion systems for modelling cell-environment feedbacks
**Article Type:**	Research
**Funding Information:**	Biotechnology and Biological Sciences Research Council (BB/T010150/1) — Dr. Orkun Soyer<br>Biotechnology and Biological Sciences Research Council (BB/R016925/1) — Not applicable<br>Engineering and Physical Sciences Research Council (EP/L015374/1) — Not applicable<br>Gordon and Betty Moore Foundation (GBMF9200) — Dr. Orkun Soyer
**Abstract:**	Background: Spatial organisation plays an important role in the function of many biological systems, from cell fate specification in animal development to multi-step metabolic conversions in microbial communities. The study of such systems benefits from the use of spatially explicit computational models that combine a discrete description of cells with a continuum description of one or more chemicals diffusing within a surrounding bulk medium. These models allow the in silico testing and refinement of mechanistic hypotheses. However, most existing models of this type do not account for concurrent bulk and intracellular biochemical reactions and their possible coupling.<br>Conclusions: Here, we describe ChemChaste, an extension for the open-source C++ computational biology library Chaste. ChemChaste enables the spatial simulation of both multicellular and bulk biochemistry by expanding on Chaste's existing capabilities. In particular, ChemChaste enables: (i) simulation of an arbitrary number of spatially diffusing chemicals; (ii) spatially heterogeneous chemical diffusion coefficients; and (iii) inclusion of both bulk and intracellular biochemical reactions and their coupling. ChemChaste also introduces a file-based interface that allows users to define the parameters relating to these functional features without the need to interact directly with Chaste's core C++ code. We describe ChemChaste and demonstrate its functionality using a selection of chemical and biochemical exemplars, with a focus on demonstrating increased ability in modelling bulk chemical reactions and their coupling with intracellular reactions.<br>Availability and implementation: ChemChaste version 1.0 is a free, open-source C++ library, available via GitHub at https://github.com/OSS-Lab/ChemChaste undertheBSDlicenseandmaybefoundintheZenodoarchiveat zendodo doi .
**Corresponding Author:**	Orkun Soyer<br>University of Warwick<br>coventry, UNITED KINGDOM
**Corresponding Author Secondary Information:**	
**Corresponding Author's Institution:**	University of Warwick
**Corresponding Author's Secondary Institution:**	
**First Author:**	Orkun Soyer
**First Author Secondary Information:**	
**Order of Authors:**	Orkun Soyer<br>Connah Johnson

Powered by Editorial Manager® and ProduXion Manager® from Aries Systems Corporation

	Alexander Fletcher
**Order of Authors Secondary Information:**	
**Response to Reviewers:**	Dear Editorial office. We have now made the final edits requested on the manuscript. We hope that it is now ready for publication.
**Additional Information:**	
**Question**	**Response**
Are you submitting this manuscript to a special series or article collection?	No
**Experimental design and statistics**<br><br>Full details of the experimental design and statistical methods used should be given in the Methods section, as detailed in our Minimum Standards Reporting Checklist. Information essential to interpreting the data presented should be made available in the figure legends.<br><br>Have you included all the information requested in your manuscript?	No
If not, please give reasons for any omissions below.<br><br>as follow-up to "**Experimental design and statistics**<br><br>Full details of the experimental design and statistical methods used should be given in the Methods section, as detailed in our Minimum Standards Reporting Checklist. Information essential to interpreting the data presented should be made available in the figure legends.<br><br>Have you included all the information requested in your manuscript?<br><br>"	N/A
**Resources**<br><br>A description of all resources used, including antibodies, cell lines, animals	No

Powered by Editorial Manager® and ProduXion Manager® from Aries Systems Corporation

and software tools, with enough information to allow them to be uniquely identified, should be included in the Methods section. Authors are strongly encouraged to cite Research Resource Identifiers (RRIDs) for antibodies, model organisms and tools, where possible.<br><br>Have you included the information requested as detailed in our Minimum Standards Reporting Checklist?	
If not, please give reasons for any omissions below.<br><br>as follow-up to "**Resources**<br><br>A description of all resources used, including antibodies, cell lines, animals and software tools, with enough information to allow them to be uniquely identified, should be included in the Methods section. Authors are strongly encouraged to cite Research Resource Identifiers (RRIDs) for antibodies, model organisms and tools, where possible.<br><br>Have you included the information requested as detailed in our Minimum Standards Reporting Checklist?<br><br>"	N/A
**Availability of data and materials**<br><br>All datasets and code on which the conclusions of the paper rely must be either included in your submission or deposited in publicly available repositories (where available and ethically appropriate), referencing such data using a unique identifier in the references and in the "Availability of Data and Materials" section of your manuscript.<br><br>Have you have met the above	Yes

requirement as detailed in our Minimum Standards Reporting Checklist?	

Powered by Editorial Manager® and ProduXion Manager® from Aries Systems Corporation

Manuscript

Click here to access/download;Manuscript;ChemChaste_GigaScience_Revi

OXFORD (GIGA)^n SCIENCE

GigaScience, 2017, 1–12

doi: xx.xxxx/xxxx
Manuscript in Preparation
Paper

PAPER

ChemChaste: Simulating spatially inhomogenous biochemical reaction-diffusion systems for modelling cell-environment feedbacks

Connah G. M Johnson[1,2], Alexander G. Fletcher[3,4,*] and Orkun S. Soyer[2,*]

[1]Mathematics of Real-World Systems Doctoral Training Centre, University of Warwick, Coventry, UK and [2]School of Life Sciences, University of Warwick, Coventry, UK and [3]School of Mathematics & Statistics, University of Sheffield, Sheffield, UK and [4]Bateson Centre, University of Sheffield, Sheffield, UK

[*]O.Soyer@warwick.ac.uk; a.g.fletcher@sheffield.ac.uk

Abstract

Background: Spatial organisation plays an important role in the function of many biological systems, from cell fate specification in animal development to multi-step metabolic conversions in microbial communities. The study of such systems benefits from the use of spatially explicit computational models that combine a discrete description of cells with a continuum description of one or more chemicals diffusing within a surrounding bulk medium. These models allow the *in silico* testing and refinement of mechanistic hypotheses. However, most existing models of this type do not account for concurrent bulk and intracellular biochemical reactions and their possible coupling.
Conclusions: Here, we describe ChemChaste, an extension for the open-source C++ computational biology library Chaste. ChemChaste enables the spatial simulation of both multicellular and bulk biochemistry by expanding on Chaste's existing capabilities. In particular, ChemChaste enables: (i) simulation of an arbitrary number of spatially diffusing chemicals; (ii) spatially heterogeneous chemical diffusion coefficients; and (iii) inclusion of both bulk and intracellular biochemical reactions and their coupling. ChemChaste also introduces a file-based interface that allows users to define the parameters relating to these functional features without the need to interact directly with Chaste's core C++ code. We describe ChemChaste and demonstrate its functionality using a selection of chemical and biochemical exemplars, with a focus on demonstrating increased ability in modelling bulk chemical reactions and their coupling with intracellular reactions.
Availability and implementation: ChemChaste version 1.0 is a free, open-source C++ library, available via GitHub at `https://github.com/OSS-Lab/ChemChaste` under the BSD license, on the Zenodo archive at `zendodo doi`, and on BioTools (biotools:chemchaste) and on SciCrunch (RRID:SCR022208) databases.

Key words: Chaste; Biofilms; Microbial Communities

Introduction

Understanding the emergent dynamics of spatially heterogeneous cell populations is highly relevant to both eukaryotic and microbial biology. Spatially self-organised biological systems often display nonlinear dynamics [1, 2, 3], which may be difficult to mechanistically explain through observation alone, necessitating the use of computational modelling approaches to help guide and explain experimental studies. Several outstanding challenges must be addressed to fully leverage models of spatially organised biological systems [4], not least the development of robust and extensive computational frameworks that allow users to define, explore, and share models in a straightforward manner.

Many computational frameworks already exist for studying the

Compiled on: April 29, 2022.
Draft manuscript prepared by the author.

Key Points

- Modelling an arbitrary number of spatially diffusing chemicals in a spatial field of cells.
- Ability to account for spatially heterogeneous chemical diffusion coefficients in a spatial field of cells.
- Modelling of both bulk and intracellular biochemical reactions and their coupling in a spatial field of cells.

dynamics of spatially organised cell populations. Some of these, such as iDynoMiCs [5], use a bottom-up (discrete, agent-based) approach to modelling individual cell behaviours [6], combined with a top-down (continuum, partial differential equation (PDE) based) approach to modelling the diffusive transport of nutrients and other chemicals. In this approach, some aspects of cell physiology are 'hard-coded', along with specific 'rules' governing their dynamics. In other computational frameworks, the physical forces acting on individual cells are modelled explicitly, but cell physiology is not. In these approaches, cells are treated as extended shapes in space, with cell proliferation and migration implemented through neighbourhood update rules, e.g. an implementation of the so-called cellular Potts model (e.g. as done in CompuCell3D [7] and as used in Morpheus [8]). It is also possible to combine these two approaches, into what we call a 'hybrid continuum-discrete approach', where cells are represented by particles, with some aspects of their physiology encoded by rules (e.g. cell division) and others governed by spatially explicit energy or force equations (e.g. cell migration). Such hybrid approaches have been developed by either creating dedicated, new computational frameworks (e.g. HAL [9], PhysiCell [10], Chaste [11]), or by adapting existing agent-based [12] or molecular dynamics [13] tools.

Using hybrid modelling tools, cell physiology can theoretically be coupled to the dynamics of chemicals in the bulk medium. This functionality, however, is implemented in a limited fashion in existing platforms. For example, in Chaste, PhysiCell and CompuCell3D, either only a limited number of bulk chemicals can be dynamically modelled, and/or diffusion coefficients are assumed to be homogeneous. Additionally, the linking of these bulk chemicals to intracellular reactions is limited in terms of number of reactions and couplings that can be encoded in each cell and at the cell-bulk interface. This limits the range of biological phenomena that can be studied within existing computational frameworks.

The coupling between cells and their microenvironment is increasingly being recognised as playing a fundamental role in cell dynamics in the context of both microbial and eukaryotic populations, e.g. metabolic environmental feedbacks in the tumour microenvironment [14] and microbial community stability [15]. Additional feedbacks can emerge from cell-excreted enzymes, which introduce reactions in the bulk, and from cell-excreted metabolites or proteins that can affect chemical diffusion coefficients in the bulk or near cells. Such effects arising from bulk-cell interaction can create their own nonlinear dynamics [16, 17, 18, 19] or exert a feedback onto cellular physiology [20, 21, 22]. Thus, modelling of metabolic and other feedbacks between bulk environment and cellular behaviours would benefit from the further development of computational frameworks centred on the role of chemical coupling.

To this end, we introduce ChemChaste, a computational framework that allows the simulation of any number of chemical reaction-diffusion systems with or without cells, and allows cell-excreted chemicals or enzymes to react in the bulk phase. ChemChaste builds upon Chaste (`https://github.com/Chaste/Chaste`) and expands its capabilities with the introduction of: (i) unlimited number of PDEs for modelling any number of bulk chemicals diffusion dynamics; (ii) heterogeneous diffusion rates, allowing for implementation of different 'domains' in the bulk pertaining different diffusion properties; (iii) expansion of the size of the cellular reaction network that can be implemented to describe cellular behaviours; and (iv) a user-interface for defining model structure. The user-interface allows cell-internal biochemical reaction systems (cell network ODEs), spatial reactions in the bulk, and heterogeneous diffusion rates for chemicals in the bulk to be encoded in a file-based system. These features allow easier simulations in ChemChaste, without any need for users to change the C++ source code. Below, we demonstrate the ChemChaste implementation and functionality using a set of chemical and biochemical exemplars, including a cell-based example. All of the source code and user manuals for ChemChaste are provided through GitHub (`https://github.com/OSS-Lab/ChemChaste`) as an open-source library to accompany Chaste, allowing for its application and further development by the research community.

Methods

ChemChaste builds from Chaste, inheriting its adaptable and modular C++ structure [23, 11], and expanding its capabilities with a comprehensive set of C++ classes (Figure 1). Chaste exhibits many capabilities ideal for the foundation of a hybrid modelling framework, including: (i) implementation of a range of on-lattice and off-lattice multicellular modelling approaches in a consistent computational framework [24]; (ii) centre-based cell modelling, which treats cells as point particles with radii of interactions [25]; (iii) accounting for cell physiology through empirical rules or a limited intracellular reaction network implemented as a set of ordinary differential equations (ODEs); (iv) modelling of cell physics, including movement and attachment;and (v) modelling of bulk chemicals dynamics using PDEs solved numerically using the finite element (FE) method [24]. For specific biological modelling applications, Chaste requires the PDEs and ODEs to be explicitly written by the user as C++ classes, limiting Chaste's usability to those familiar with C++ [26, 27, 28]

Expanding from Chaste, ChemChaste considers parabolic reaction-diffusion systems, where chemicals diffusing and reacting in the bulk are also coupled with cells present in the same bulk, through cellular excretion and uptake. For simulating such cell-bulk coupling, ChemChaste is developed to handle different chemical species confined to the bulk, to cell populations, or present in both phases. ChemChaste also allows for spatially varying chemical diffusion coefficients.

Each ChemChaste simulation features four distinct dynamical components that run at each discrete time step of the simulation (Figure 1-b). These involve updating of bulk and cellular chemical systems, their couplings, cell behaviours, and cell positions. The bulk and cellular chemical reaction systems are considered separately: the former is updated by solving reaction-diffusion equations, taking into account any reactions implemented in the bulk; while the latter may in general differ from the bulk chemical system and may involve further chemical species. These two systems are coupled through transport of chemicals across the cell membrane. Thus, bulk chemical concentrations are updated according to these couplings. After all chemical concentrations have been updated, any 'rules' implemented regarding cell behaviour (e.g. division) are checked and subsequent cellular events (e.g. cell

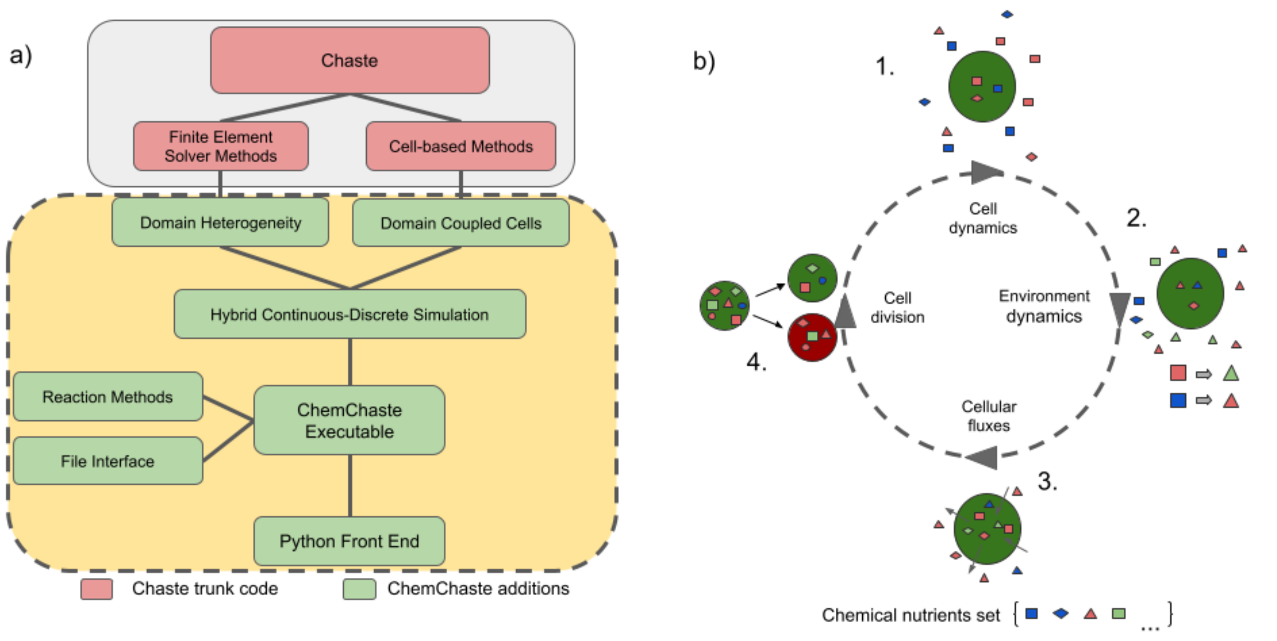

Figure 1. ChemChaste's simulation framework. a) ChemChaste classes (in dashed yellow-green) that extend Chaste's FE solver capabilities. These build on existing Chaste modules (in solid gray-pink) and allow for heterogeneous spatial domains with varying diffusion rates for chemicals. The cell-based methods are also extended through introducing transport properties linking cell interior and exterior state variables. These extensions are coupled with a file-based user interface allowing higher-level model specification. b) Four processes that occur over discrete time steps and allow the simulation of cells coupled to the bulk. The cells perform their own system of rules or reactions (cell cycle progression, cell properties, and cellular reaction networks) (1 & 2) before the environmental reaction-diffusion systems are solved (2). The state variables are then coupled through cellular flux through the transport processes and membrane reactions (3), before any implemented cell-based rules (e.g. relating to cell division and/or death) are performed (4).

death, division) are implemented. Division introduces a daughter cell into the simulation. In this case, the cellular chemicals of the parent cell are re-distributed between both cells, based on a user-defined parameter (allowing for symmetric or asymmetric inheritance of cellular chemicals). The location of each cell is updated by numerically integrating its equation of motion. These two steps, division and movement, are inherited from Chaste [11]. The user may tailor the simulation details through a file interface system. Further details of the ChemChaste platform are explained below and in the Supplementary Information (SI).

Expanding the reaction-diffusion system simulations: The `Domain Field` Class

The core of Chaste is composed of finite element (FE) solvers and associated spatial meshing routines (see SI section S1 for details of the FE method as implemented in Chaste). In brief, the FE methods model the bulk domain as a discrete mesh of nodes and approximates the concentration of each chemical across this mesh, subject to a user-defined combination of boundary conditions (BCs): Neumann; Dirichlet; or periodic conditions at the edge of the bulk domain. Over the mesh, Chaste utilises a range of ODE solvers, chosen by the user, to determine the ODE solutions at the discrete mesh nodes. Utilising a set of linear basis functions, these nodal ODE solutions are then interpolated onto a finer grid of points, known as Gauss points, where point-based source terms and diffusive terms are added. Chaste's FE method then uses the chemical values at the Gauss points to compute the PDE system solutions at the next time step. This implementation has been limited in Chaste to solving the same given ODE for all nodes in the mesh.

Expanding from this implementation, ChemChaste introduces a `Domain Field` class, which allows us to compute the solution of nodal ODEs generally varying at each mesh node. With the addition of the chemical and reaction classes (see SI, sections S1.3–S1.4), ChemChaste forms a chemical `Domain Field` wherein the concrete reaction systems are mapped to the FE mesh. This expansion allows for: (i) multiple, diffusing bulk chemicals; (ii) reactions among chemicals in the bulk; and (iii) spatially varying diffusion rates for chemicals. With this introduction the simulation domain may now be broken into sub-domains, each containing their own diffusion parameters, ODE systems, and node-based source terms. This allows chemical reaction systems to be confined to sub-domains of the simulation for modelling spatial sub-compartments with their own diffusion parameters, e.g. a biofilm or tissue surrounded by a bulk. The `Domain Field` class uses a 2D matrix to contain the nodal values which acts as a look up reference for spatial aspects of the simulation. While this currently limits the ChemChaste simulation to a 2D domain, an extension to 3D simulations would be straightforward for a C++ proficient user by editing the source code.

Coupling the cell physiology and reaction-diffusion system simulations

The core spatial mesh routines of Chaste also form the basis of simulating dynamic cell populations. ChemChaste uses the 'node-based' or cell-centre modelling approach offered in Chaste [25]. In this approach, a cellular mesh (CM) is defined wherein each mesh node acts as the centre of a cell. Each cell is simulated as a particle, and the CM vertices are used to encode any rules (e.g. physical forces) governing physical cell interactions [24, 26]. The CM is also mutable, allowing simulation of cell motility - by defining forces to shift CM nodes - or cell division and death - by performing vertex additions or deletions on the CM [23]. In ChemChaste, cell motility is provided by the passive shunting when new cells are introduced through cell division. Active motility laws are implemented in the Chaste package and can be used by modifying the ChemChaste source code. However this would bypass the file-based user interface and would not benefit from the

ChemChaste features.

ChemChaste expands upon this node-based cell population simulation to introduce the coupling between cellular and bulk chemicals. As explained above, an interpolated Gauss point is produced during the FE simulations. In ChemChaste, this point may also be the location of a cell in the CM where the 'volume' of the point-like cell matches the FE mesh point volume share of the environment. When this is the case, membrane and transport reactions are performed on the selected cell and their outcomes are coupled to the relevant cellular and bulk chemicals. In this way the cell's 'contribution' to the source term of the related, bulk chemicals' reaction-diffusion PDE is accounted for. At the same time the selected cell's internal chemical concentrations are updated through exchanged chemicals (see SI, section S1.2).

Specifying chemical reactions and chemicals diffusion properties

ChemChaste allows modelling of three different reaction processes based on where they occur; bulk, membrane, and transport reaction. Bulk reactions offer the means to model reactions in the bulk and acting on spatially diffusing chemical species. As explained above, the FE simulations implement on each node of the mesh a reaction rule, which is used to update species' concentrations accordingly. Bulk reactions occur on these mesh nodes and act as a source/sink term for the PDEs defining the reaction-diffusion system. Membrane and transport reactions involve cellular and bulk chemical species and therefore require knowledge of the concentrations of a given chemical both within the cell object and in the bulk. In the case of membrane reactions, reaction rates depend on both bulk and intracellular chemical concentrations, however, there is no chemical species exchange through the membrane. This class of reactions is thus ideal for implementing processes such as membrane bound enzymatic reactions. Transport reactions implement a chemical flux through the membrane and internal species may react or exchange with external species.

The three reaction types are modelled with user-defined kinetic rate laws, such as mass action or enzymatic kinetics. In ChemChaste, both the stoichiometry and kinetic rates of these reactions are defined through a file-based user interface (see next section and SI, section S2.2.2). Furthermore, bulk reactions can be assigned to a specific sub-domain (of the `Domain Field`) of the mesh. To assist with the assignment of kinetic laws to reactions, ChemChaste implements specific classes describing different kinetic laws. In ChemChaste, chemical species may be provided with a set of properties: name, diffusivity, mass, valence, Gibbs formation free energy. These properties can be linked to affect the rate of diffusion or rate of a given reaction within which the species participate. Furthermore, when the `Domain Field` contains sub-domains, the domain varying chemicals' properties may be stored in upstream inheritance classes. This allows simulating changes in diffusivity due to spatial heterogeneities (e.g. bulk media vs. biofilm or tissue). Within the ChemChaste code, these chemical associated parameters can be called by the PDE diffusion functions or reaction systems for the correct sub-domain.

File-based user interface

ChemChaste introduces a file-based interface to enable its use by a wider audience. In particular, ChemChaste has two main user-interface systems, one to provide the `Domain Field` and diffusion properties and one for defining the `Reaction System`, which together characterise a heterogeneous reaction-diffusion model. The `Domain Field` files contain the information required to produce the FE mesh and define the labelled sub-domains. This file also defines any varying BCs and/or diffusion rates for bulk chemicals. The user supplies a comma separated values (CSV) file of labels denoting the sub-domains and a text file of the associated label keys (see SI, section S2.2 for an exemplar `Domain Field` file). Further CSV files of initial species values, boundary conditions, and diffusion rates on a sub-domain basis may also be specified. These files fully characterise the conditions of the simulation space, while the reaction dynamics are detailed in a separate reaction file.

The `Reaction System` file encodes the bulk, cellular, and coupling (i.e. membrane and transport) reactions as described above. For the bulk reactions each sub-domain can have an associated, separate reaction system file. Another file is used to define the cellular reaction system. Within this cell file, coupling reactions are defined with at most one membrane reaction file and one transport reaction file, each containing a set of reactions of the respective type. All reaction files follow a set format; name of reaction kinetics, chemical equation involving the species, then the kinetic parameters used by the rate laws (see SI, section S2.2.2). Further rate laws may be implemented by the user, which will then be utilised in the same way as the supplied rate laws (see SI, sections S4–S6 for details). Overall, the information stored within these files is sufficient to select the desired reaction class, formulate reaction terms and implement concentration changes when solved within the simulation.

Results

ChemChaste presents a hybrid continuum-discrete modelling framework for the simulation of individual cells within a chemically active environment. As shown in Figure 1 and discussed in the Methods section, the framework is composed of an array of different modules building upon each other to fulfil the simulation needs. Here, we verify and demonstrate the functionality of ChemChaste by considering each of these key modules in turn. The accuracy of the PDE solvers was tested through solving the Fisher-Kolmogorov-Petrovsky-Piskunov (Fisher-KPP) equation showing a strong agreement with an analytic series expansion. The simulation of multiple PDEs using the ChemChaste reaction system and file interface system was demonstrated through producing diffusion-driven spatial patterning and temporal oscillations of the Schnakenberg reaction system (Section). Finally, an exemplar coupled cell simulation was implemented involving a cooperator-cheater system based on enzyme excretion (Section).

Spatial simulation accuracy in ChemChaste: Fisher-KPP equation

To verify and demonstrate the PDE solving capabilities in ChemChaste, a single PDE with a known analytical solution was implemented. The chosen system was the Fisher-KPP equation, which has been used to model the propagation of an invasive species through a population [29, 30] and admits travelling wave solutions with an analytically resolved minimum wave velocity [31]. The corresponding reaction-diffusion equation includes a logistic growth source term,

$$\frac{\partial U}{\partial t} - D\nabla^2 U = rU\left(1 - \frac{U}{\kappa}\right), \tag{1}$$

where $U(\mathbf{x}, t) \geq 0$ is the size of the invasive species population at position $\mathbf{x} = (x, y)$ and time t, and the positive parameters D, r and κ denote the diffusion coefficient, linear growth rate and carrying capacity of the invasive species, respectively. For suitable initial conditions, it is known that this system exhibits pulled travelling wave solutions of the form $U(z)$ where $z = x - ct$ and $c \geq 0$ is the wave velocity. It can be shown analytically that the front of these waves travels with a minimum velocity defined by

$$c_{min} = 2\sqrt{rD}, \tag{2}$$

while the observed velocity, $c \geq c_{min}$, is dependent on the initial conditions [30, 31].

We implemented the Fisher-KPP equation in a ChemChaste simulation using equation (1) and setting the parameters to unity $\{D, r, \kappa\} = 1$. We considered a rectangular bounded domain $\Omega \in [0, 10] \times [0, 100]$ and impose zero-flux boundary conditions (BCs) and record a 1-dimensional slice across the domain. The simulations were initialised with a strip of invasive species bordering the left boundary of the domain, $0 < x < 1$:

$$U(x, y, 0) = U_0 \quad \text{for} \quad 0 < x < 1, 0 < y < 100. \tag{3}$$

For equation (1) the minimum wave speed with the selected parameter set is given by $c_{min} = 2$.

The FE methods within ChemChaste were used to solve equation (1) subject to the boundary and initial conditions. A travelling wave solution was identified across the one-dimensional domain slice and compared to the analytical solution of the one-dimensional Fisher-KPP equation [32], given by

$$\begin{aligned} \mathrm{U(x, y, t)} = &\frac{1}{1 + \exp(z/c)} \\ &+ \frac{c^{-2}\exp(z/c)}{(1 + \exp(z/c))^2} \ln\left(\frac{4\exp(z/c)}{(1 + \exp(z/c))^2}\right) \\ &+ O\left(\frac{1}{c^4}\right) \end{aligned} \tag{4}$$

where $z = x - ct$ denotes the travelling wave coordinate.

The results were visualised using ParaView [33]. Two tests were considered: comparing the travelling wave front solution produced by the ChemChaste simulation vs. the analytic form given by equation (4), and comparing the simulations' convergence stability under decreasing temporal and spatial step size. Results for both tests are given in Figure 2, and show a good agreement between the ChemChaste simulation output and expected results determined through analytic solutions. Additionally, the convergence with decreasing temporal and spatial step sizes suggest stable numerics albeit with the waves showing longer accelerating phases than the expected analytic top-hat gradient. Therefore the ChemChaste implementation was able to correctly simulate dynamics (in this case, the travelling wave phenomenon) in simple PDE with stable and accurate numerics.

Modelling multiple, diffusing and reacting chemicals in ChemChaste: Schnakenberg reaction-diffusion system

ChemChaste builds upon Chaste's PDE solvers to enable the simulation of multiple PDEs over the domain. While Chaste is restricted to solving three PDEs, ChemChaste's limiting factor is solely the available computational resources. To test the multi-dimensional PDE simulation, and to verify the file interface system, we implemented the well-studied two species reaction system commonly known as the Schnakenberg system [34, 35] and shown in equations (5)–(7). When these reactions are modelled with mass action kinetics they are shown to display temporal oscillations and diffusion driven spatial patterning for distinct, defined parameter regimes [36, 37]. These phenomena were reproduced here using ChemChaste.

The Schnakenberg reaction system involves two chemical species U, V which are produced, inter-converted, and removed via the reactions

$$\emptyset \underset{k_{-1}}{\overset{k_1}{\rightleftharpoons}} U, \tag{5}$$

$$\emptyset \xrightarrow{k_2} V, \tag{6}$$

$$2U + V \xrightarrow{k_3} 3U, \tag{7}$$

Table 1. Parameters used in the Schnakenberg reaction simulation. The values were selected based on analytical solutions of this system and to demonstrate the possible oscillatory and patterning dynamics.

Case	k_1	k_{-1}	k_2	k_3	D_U	D_V	U_0	V_0
Figure 3a	0.5	2.2	1.5	1.0	0.5	0.5	0.91	1.67
Figure 3b	0.1	1.0	0.9	1.0	1	40	1.0	1.0

where the reaction rate constants are denoted by k_1, k_{-1}, k_2, k_3. Applying mass action kinetics to these reactions yields the reaction ODEs

$$\frac{dU}{dt} = R_U(U, V) = k_1 - k_{-1}U + k_3VU^2, \tag{8}$$

$$\frac{dV}{dt} = R_V(U, V) = k_2 - k_3VU^2, \tag{9}$$

where the reaction rates R_U, R_V describe the change of each species' concentration in a given timestep and also provide the source terms to the reaction-diffusion PDEs. The PDEs are satisfied across the whole two-dimensional domain space, Ω, and are given by

$$\frac{\partial U}{\partial t} - D_U\nabla^2 U = R_U(U, V), \tag{10}$$

$$\frac{\partial V}{\partial t} - D_V\nabla^2 V = R_V(U, V), \tag{11}$$

where D_U, D_V are the spatially homogeneous isotropic diffusion coefficients. Here, we consider a square bounded domain $\Omega \in [0, 100] \times [0, 100]$ which are subject to zero-flux Neumann BCs

$$\mathbf{n} \cdot \nabla U = \mathbf{n} \cdot \nabla V = 0 \quad \text{on} \quad \partial\Omega. \tag{12}$$

Each simulation begins with the randomly perturbed initial conditions defined on each node of the FE mesh,

$$U(x, y, 0) = U_0 + \xi, \tag{13}$$

$$V(x, y, 0) = V_0 + \zeta, \tag{14}$$

where $\xi, \zeta \sim \mathit{Uniform}(-1, 1)$ are uniformly distributed random fields bounded by the interval $[-1, 1]$.

Two parameter sets were considered: one for temporal oscillations; and one for diffusion-driven patterning [36]. Temporal oscillations are present when the homogeneous system, equations (8)–(9), display limit cycle behaviour. Spatial patterning across the domain occurs when the spatially uniform steady-state solution to equations (10)–(11) is linearly stable in the absence of diffusion ($D_U = D_V = 0$), but linearly unstable in the presence of diffusion. The resultant spatial patterning in the 2D concentration maps are referred to as displaying diffusion-driven instabilities (DDI) or Turing instabilities [37, 38, 39, 40]. These dynamical cases were found to occur for specific parameter sets, as listed in Table 1.

These parameters were determined through considering small linear perturbations for conditions which provided the expected phenomena in the two cases, equations (8)–(9) and (10)–(11), and selecting parameter sets which satisfy the algebraic equations [37], (see SI, section S3 for details). The values U_0, V_0 were used as the initial conditions for the two cases.

We have verified, using ChemChaste, that this model exhibits the expected spatio-temporal dynamics for the tested parameter regimes (see Figure 3). These results are as expected for the parameters used, based on analysis of equations (8)–(9) and (10)–(11). Therefore these tests verify that ChemChaste was able to both correctly parse the chemical reaction files and simulate multi-chemical reaction-diffusion systems capable of complex dynamics and patterning.

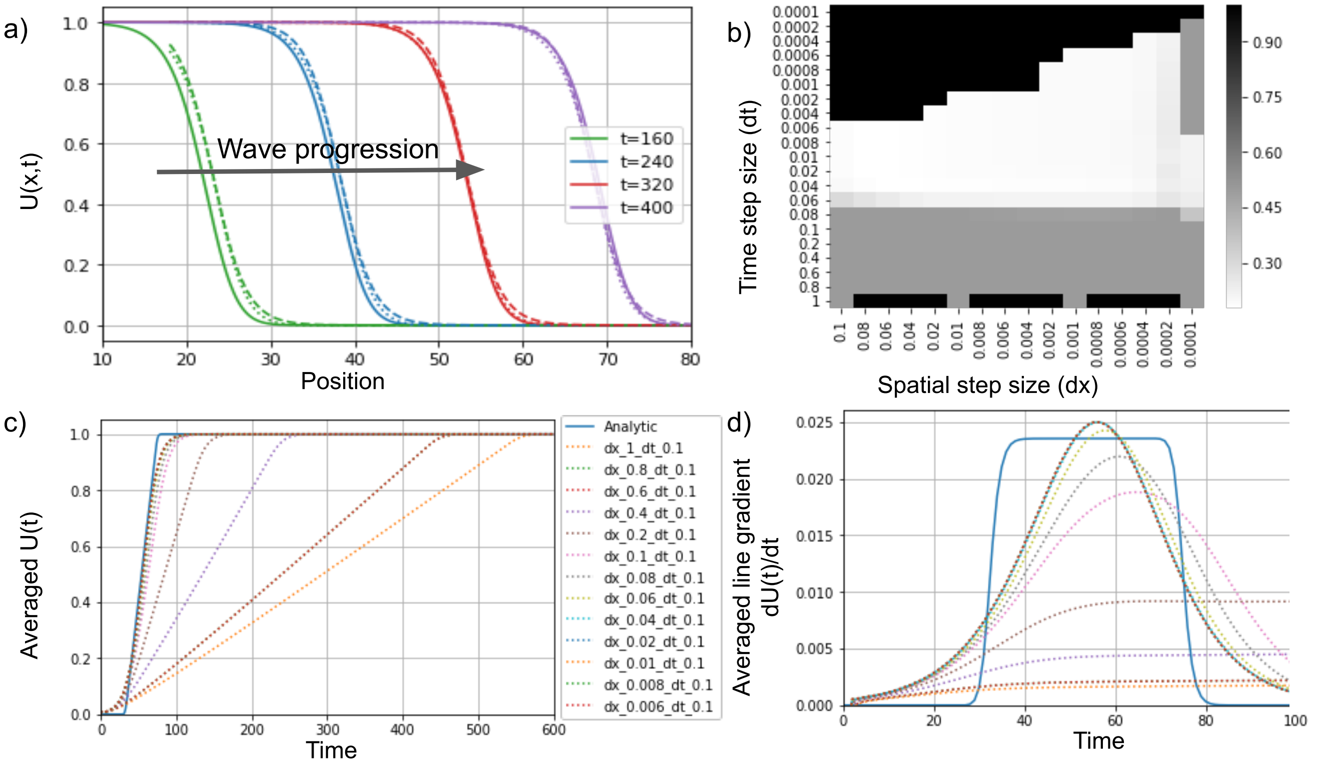

Figure 2. ChemChaste simulations of the Fisher-KPP equation. a) Plot showing the progression of an expanding wavefront through the domain (solid line). The simulation results are accompanied by the analytic solution for the zeroth (dashed line) and first order (dotted line) expansion in terms of $1/c^2$ in equation (4). The wave speed in simulation is initially faster than the analytical minimum wave speed $c_{min} = 2$, calculated with equation (2), but with agreement at later times implying the correct asymptotic wave velocity has been reached. b) Heat-map of L^2 convergence scores for simulations using a range of spatial and temporal step sizes. The simulations for given step sizes are compared to the analytically determined value with the lower scores suggesting closer values. A threshold was utilised reducing higher scores to 0.5 (gray pixels). This includes simulations whose numerics diverged. A second source of ill convergence occurs when the linear algebra routines fail to complete within in-built tolerance ranges. These areas are represented by an elevated score or 1.0 (black pixels). c) Traces for the solutions U(t) averaged across the domain for different spatial and temporal step sizes. The traces converge to the analytical solution with decreasing step size. d) The gradients of the slopes in plot c) sharing the same legend. The gradients are suggestive of the velocity of the wave passing through the domain.

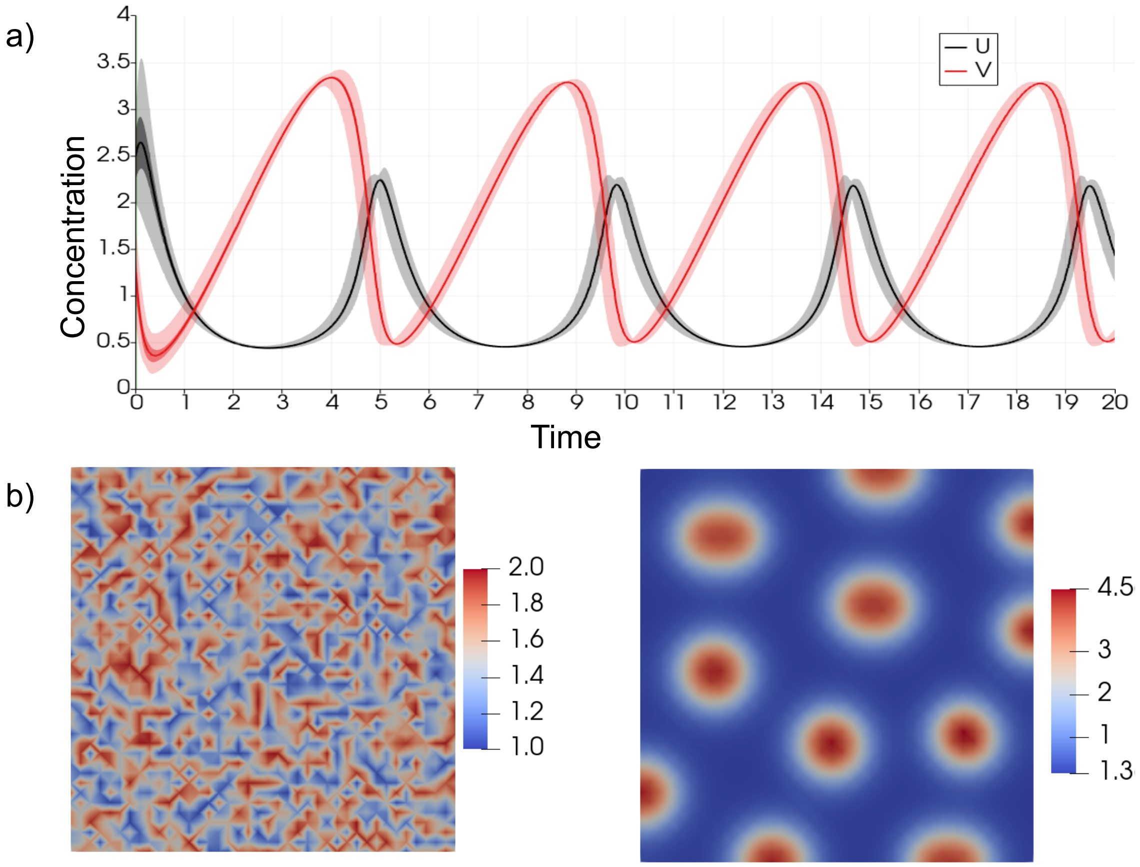

Figure 3. The Schnakenberg reaction system showing the oscillatory and patterning dynamics. a) The two curves show the concentration of U and V averaged over the nodes in the domain for each time step from the simulations run using oscillatory regime parameters. The concentration traces show the range (light), quartiles (darker), and the spatially averaged concentration (dark) for both chemicals. b) Domain maps of the initial and final (i.e. steady state) distribution of U and V in simulations using parameters for the patterning dynamics (see Table 1). The initial distribution is formed by the addition of uniform random noise at each node point (see equations 13 and 14).

Coupled cell-chemical environment simulations in ChemChaste

A main motivation behind developing ChemChaste was to simulate a hybrid continuum-discrete model of cells within a chemically reactive environment, where bulk and cell-secreted chemicals and other entities such as proteins can diffuse as well as react. This is a common biological scenario, as seen for example in the case of microbial utilisation of cellulose or other complex resources, which must be treated by enzymes before a cell can metabolise or uptake them [41]. The core aspects of this scenario, i.e. a cell-secreted enzyme mediating a reaction in the bulk is also found in cases outside of substrate uptake, for example in de-toxification of the environment [42]. In ChemChaste, this scenario is readily modelled through implementation of bulk reactions and coupling of cellular metabolic reactions and environmental PDEs.

Here, we provide a simplistic, toy example for illustrative purposes and for testing ChemChaste implementation of cellular reactions and cell-environment coupling. More detailed and realistic simulations can be readily constructed by users, through developed ChemChaste user interface. For the exemplar test case, we modelled a growing cell population harbouring two cell types, along with a chemical resource (i.e. substrate) that is not readily taken up. One cell type - termed cooperator - excretes an enzyme that can allow the internalisation of the substrate, while the other cell type - termed cheater - does not excrete the enzyme but can also internalise the enzyme-bound substrate (Figure 4a). The cells process the internalised substrate to produce a pseudo chemical species (called ‘biomass’), which is used as a proxy for monitoring cell growth. Once the cellular biomass concentration reaches a threshold value the cell divides into two, the parent and offspring, sharing the internal concentrations equally between both parent and offspring cell. The offspring cell is placed at a random neighbouring location around the parent cell and the population undergoes positional updating to accommodate the new cell.

Previous agent-based simulations of growing cell populations harbouring cheater and cooperator types have found spatial segregation of cell types within the population [43, 44, 45]. This cell sorting is linked to the disparity in growth rates of the two species, which may be due to substrate availability and dependency, and is of interest in game theoretic investigations of mutual interactions in biofilms [46, 47]. The presented simulations are conceptually similar to these previous studies, but differ in their mechanistic implementation of substrate scavenging, as a cooperative trait, as well as the inclusion of both substrate and oxygen diffusion in the bulk.

In the presented model the two types of cells were introduced into the simulation domain which contains two chemicals which diffuse in the bulk; oxygen (O_2) and a substrate, S. Furthermore the cells excrete and take up a scavenging enzyme, E, the enzyme-substrate complex, ES, and O_2, which freely diffuses in the bulk. To capture dynamics of cell growth, a simple metabolic network is implemented in each cell, defined by the following toy reactions that abstract biomass generation and the main respiratory and fermentative metabolic pathways:

$$ES \xrightarrow{k_1} E + S,$$

$$S + \mathrm{NAD}^+ + \mathrm{ADP} \xrightarrow{k_2} \mathrm{Precursor} + \mathrm{NADH} + \mathrm{ATP},$$

$$\mathrm{NADH} + \mathrm{ADP} + \mathrm{O}_2 \underset{k_{-3}}{\overset{k_3}{\rightleftharpoons}} \mathrm{NAD}^+ + \mathrm{H_2O} + \mathrm{ATP},$$

$$\mathrm{Precursor} + \mathrm{ATP} \xrightarrow{k_4} \mathrm{Biomass} + \mathrm{ADP},$$

$$\mathrm{Precursor} + \mathrm{ATP} \xrightarrow{k_5} E + \mathrm{ADP},$$

where NAD^+, NADH, ADP, and ATP are the usual energy and electron carrier molecules internal to the cell. These toy reaction set captures substrate uptake (reaction 1), re-cycling of NAD^+/NADH and ADP/ATP pairs through fermentative and respiratory pathways (reactions 2 and 3), and biomass and scavenging enzyme production through ATP investment (reactions 4 and 5). For the simulations, these reactions are modelled with mass action kinetics with shown reaction rate constants. All reaction rate constants were set to 1 in both cell types, except for k_5, which is set to zero in the cheater cell type. The overall simulation schematic for this cellular system is shown in Figure 4.

In addition to the cellular reaction network, we implemented bulk reactions for the enzyme binding to the substrate in the extracellular media, the enzyme being degraded in the bulk, and the diffusion of the substrate (S), enzyme (E) and the enzyme-substrate (ES) complex.

$$ES \underset{k_{-1}}{\overset{k_1}{\rightleftharpoons}} E + S,$$

$$E \xrightarrow{k_2} \emptyset.$$

The parameters for these reactions were scaled for computational efficiency and are given in SI, section S2.3. We performed simulations through the hybrid continuum-discrete solvers introduced in ChemChaste. A reaction-diffusion PDE was solved over the domain for the diffusing species $\{E, S, ES, O_2\}$ with Neumann BCs at the domain boundary. The Neumann boundary conditions allow continual replenishment of substrate to drive the system. The cells were placed in the centre of this domain with a single cell of each type, and allowed to grow over the simulation course, as shown in Figure 5. The chemical concentrations in each cell and the bulk were recorded over the simulation. Note that initial substrate levels at the beginning of the simulation are low, but will linearly increase due to the implementation of the Neumann boundary conditions. Additional boundary conditions, like Dirichlet type, can be defined per the user files.

We show the dynamics of cellular and bulk chemicals in Figure 4 and 5. While Figure 4 is focused on the cell concentrations, Figure 5 demonstrates the impact that the cells have on local chemical concentrations. In Figure 5a, we see higher enzyme concentrations in the vicinity of cooperator cells. This is as expected, since these are the cells excreting the enzyme. We expect that such higher local concentrations of enzyme will be enhanced with lower enzyme diffusion rates and enzyme degradation rate in the bulk. In Figure 5b, we see the substrate concentration, with higher values at the domain edge (due to influx of substrate) and lower values near the cell population (due to cellular uptake). Evaluating Figures 4 and 5, together, we see a greater uptake of the substrate by the cooperator cells and a greater rate of cell biomass increase, compared to the cheater cells. Thus, the localised pockets of high enzyme concentrations around cooperator cells can lead to their growth rate surpassing that of cheaters and subsequently lead to a spatial segregation of the two cell types. While further simulations with different parameter sets are needed to fully confirm these dynamics, the presented results provide an exemplar implementation of cellular simulations in ChemChaste and confirm expected cooperator-cheater dynamics.

We conclude that the presented toy model and exemplar implementation of a cellular simulation demonstrate ChemChaste’s flexibility and capabilities in developing models featuring cell-environment coupling along with environmental reaction-diffusion.

Conclusion

We have presented ChemChaste, a computational framework for hybrid continuum-discrete modelling of multi-cellular populations coupled to chemical reaction-diffusion systems. In contrast

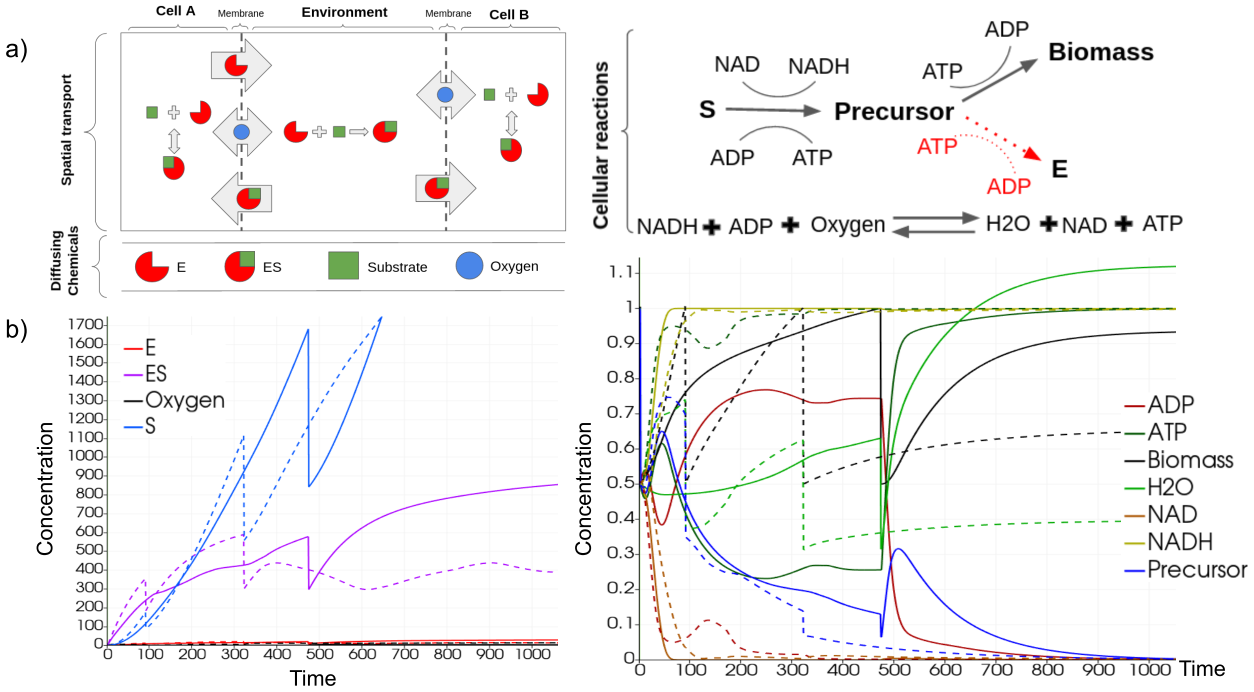

Figure 4. The simulation schematic and results for the exemplar cellular model with cell-environment coupling. a) Cartoon showing the two cell types and cellular reaction system implemented in the simulations. One cell type, the 'cooperator', excretes an enzyme that can bind an environmental substrate, while the other - the 'cheater' - does not produce the enzyme (left). Both cell types can take up the enzyme-substrate complex and process it through a series of internal reactions (right). Note that the enzyme producing pathway is only active in the cooperator cells, which has to invest substrate between this pathway and biomass producing pathway. b) The concentrations for each chemical within the cell are displayed over time for a cell of both types; cooperator (solid) and cheater (dashed) lines. The main plot (left) shows the concentrations of ES and S (chemicals harvested from the environment). The inset (right) shows the concentrations of the cell-internal chemicals. Sharp changes in cellular concentrations are due to cell division and sharing of chemicals between the parent and offspring.

to existing computational frameworks, ChemChaste facilitates chemical couplings between bulk and cellular metabolic processes through an arbitrary number of diffusing chemicals that can undergo chemical reactions in the bulk and that can have spatially heterogeneous diffusion coefficients. ChemChaste simulations are implemented using a simple file-based interface and can be used to implement different biological and chemical scenarios for modelling complex cell-environment chemical coupling and resulting emergent phenomena.

We have presented several exemplar simulations in ChemChaste, which produce the expected dynamical behaviours in given parameter regimes. These exemplars were specifically chosen to demonstrate ChemChaste's functionality and flexibility, instead of presenting an exhaustive list of the possible phenomena that may be investigated using this tool. Applications of immediate interest can include different observed cases involving coupling between cellular physiology, cell excretions, and environmentally diffusing reactions such as metabolic switching of cell types coupled to a reactive environment [15, 48], coupled chemical reactions in the bulk and within cells [38], coupling between cell secreted enzymes, signalling, and motility [49], and cell-chemical systems presenting spatially varying diffusion coefficients (e.g. within and outside of a tissue) [20]. In the current release, cells are represented by point like agents which are effective models for disperse microbial systems where the size of a cell is in the micro- sub-micron range. Therefore juxtacrine transport is not implemented and the change in the concentration gradient over the cell is negligible. Future versions of ChemChaste may look to relaxing the cell size constraint to provide a more appropriate model for larger or filamentous cells seen in mammalian or fungi systems.

Some of these investigations may require further expansion of ChemChaste. In particular, while the underlying Chaste code is already capable of implementing 3D simulations, some modifications to the model input system and parsing routines would be required to enable ChemChaste to be used for such simulations. However, for users proficient in C++ the addition of new classes is straightforward through the addition of new user-defined classes to the ChemChaste C++ class hierarchy utilising the modular structure of the framework. In this way we hope ChemChaste will prove a useful tool for investigating the chemical mechanisms behind a range of phenomena in spatially organised biological systems.

Availability of source code and requirements

Project name: ChemChaste
Project home page: https://github.com/OSS-Lab/ChemChaste [50]
Operating system(s): Platform independent
Programming language: C++, Python
Other requirements: Docker
License: BSD 3-Clause License
Any restrictions to use by non-academics: licence needed
RRID: SCR022208

Availability of supporting data

Snapshots of our code and other data further supporting this work are openly available in the GigaScience repository GigaDB [51].

Acknowledgements

The authors thank Aydar Uatay for useful discussions about Chaste.

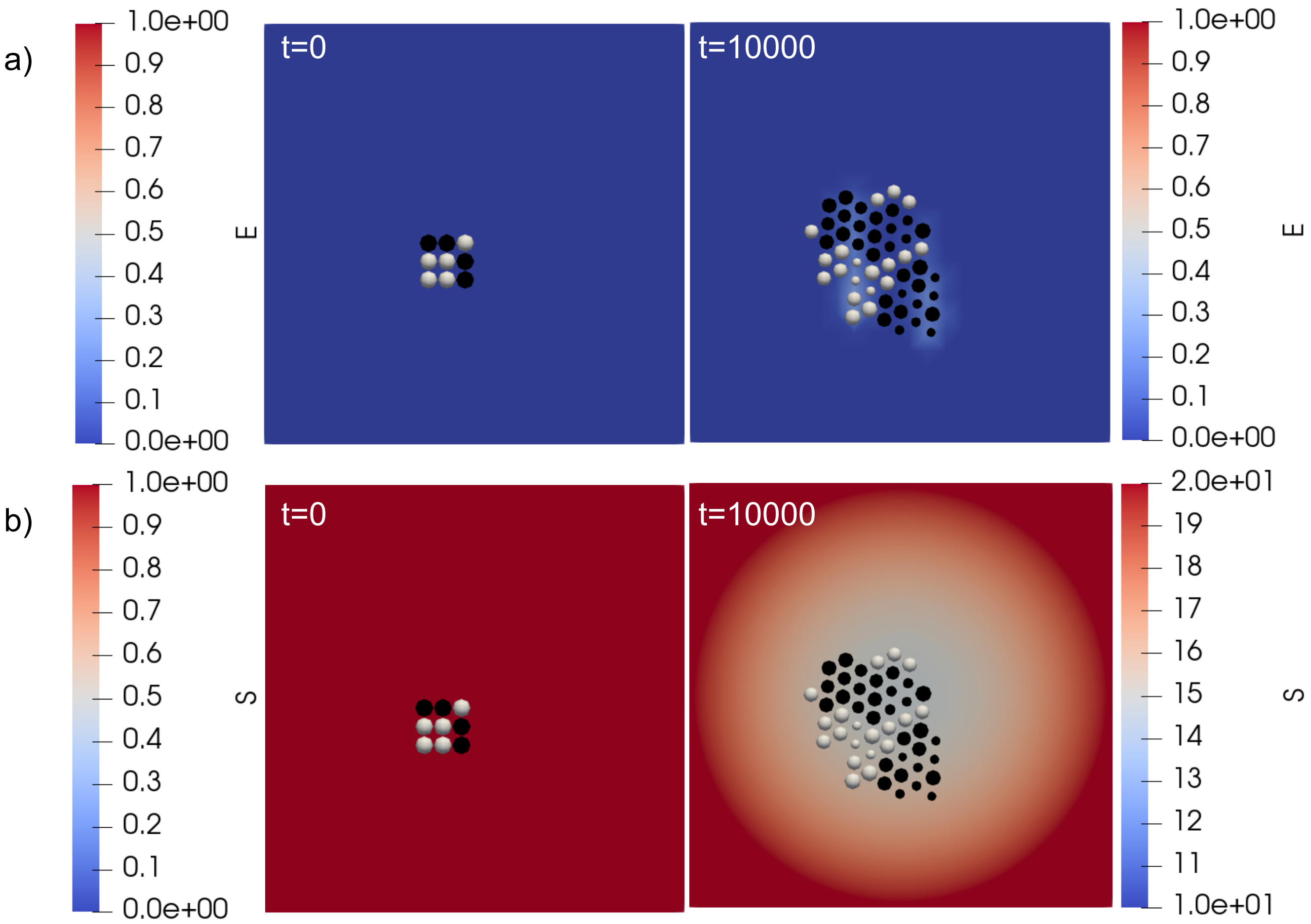

Figure 5. Domain maps showing the cells and the spatial concentration of the enzyme and nutrient species, *E* and *S*, in the bulk at the beginning, $t = 0$, and at the end, $t = 10000$, of the simulation. The size of the cells demonstrates the accumulation of 'Biomass' with larger cells being closer to dividing. The cooperator and cheater cells are coloured white and black, respectively. The upper row (a) shows simulation results with the enzyme (E) concentration plotted across the domain. The lower row (b) shows simulation results for the substrate (S) across the domain with the replenishment of the substrate at the boundary.

Funding

This work was supported by the UK's Biotechnology and Biological Sciences Research Council [BB/T010150/1 to O.S.S., BB/R016925/1 to A.G.F.] and the UK's Engineering and Physical Sciences Research Council and Medical Research Council [EP/L015374/1 to University of Warwick's Mathematics of Real World Systems Centre for Doctoral Training]. OSS acknowledges additional funding from Gordon and Betty Moore Foundation (Grant GBMF9200, https://doi.org/10.37807/GBMF9200).

Conflict of Interest: none declared.

References

1. An G, Fitzpatrick B, Christley S, Federico P, Kanarek A, Neilan RM, et al. Optimization and control of agent-based models in biology: a perspective. Bull Math Biol 2017;79:63–87.
2. Hart SF, Mi H, Green R, Xie L, Pineda JMB, Momeni B, et al. Uncovering and resolving challenges of quantitative modeling in a simplified community of interacting cells. PLoS Biol 2019;17:e3000135.
3. Painter KJ. Mathematical models for chemotaxis and their applications in self-organisation phenomena. J Theor Biol 2019;481:162–182.
4. Fletcher AG, Osborne JM. Seven challenges in the multiscale modeling of multicellular tissues. WIREs Mech Dis 2021;e1527.
5. Lardon LA, Merkey BV, Martins S, Dötsch A, Picioreanu C, Kreft JU, et al. iDynoMiCS: next-generation individual-based modelling of biofilms. Environ Microbiol 2011;13:2416–2434.
6. Kreft JU, Plugge CM, Prats C, Leveau JH, Zhang W, Hellweger FL. From genes to ecosystems in microbiology: modeling approaches and the importance of individuality. Front Microbiol 2017;8:2299.
7. Glazier JA, Graner F. Simulation of the differential adhesion driven rearrangement of biological cells. Phys Rev E 1993;47:2128.
8. Starruß J, de Back W, Brusch L, Deutsch A. Morpheus: a user-friendly modeling environment for multiscale and multicellular systems biology. Bioinformatics 2014;30:1331–1332.
9. Bravo RR, Baratchart E, West J, Schenck RO, Miller AK, Gallaher J, et al. Hybrid Automata Library: A flexible platform for hybrid modeling with real-time visualization. PLoS Comput Biol 2020;16:e1007635.
10. Ghaffarizadeh A, Heiland R, Friedman SH, Mumenthaler SM, Macklin P. PhysiCell: an open source physics-based cell simulator for 3-D multicellular systems. PLoS Comput Biol 2018;14:e1005991.
11. Cooper F, Baker R, Bernabeu M, Bordas R, Bowler L, Bueno-Orovio A, et al. Chaste: cancer, heart and soft tissue environment. J Open Source Softw 2020;5:1848.
12. Xavier JB, Picioreanu C, Van Loosdrecht MC. A framework for multidimensional modelling of activity and structure of multispecies biofilms. Environ Microbiol 2005;7:1085–1103.
13. Plimpton S. Fast parallel algorithms for short-range molecular dynamics. J Comput Phys 1995;117:1–19.
14. Carmona-Fontaine C, Deforet M, Akkari L, Thompson CB, Joyce JA, Xavier JB. Metabolic origins of spatial organization in the tumor microenvironment. Proc Natl Acad Sci USA 2017;114:2934–2939.
15. Ratzke C, Gore J. Modifying and reacting to the environmental pH can drive bacterial interactions. PLoS Biol 2018;16:e2004248.
16. Kondo S, Miura T. Reaction-diffusion model as a framework for understanding biological pattern formation. Science 2010;329:1616–1620.
17. Newman SA. 'Biogeneric' developmental processes: drivers of major transitions in animal evolution. Phil Trans R Soc B 2016;371:20150443.
18. Höfer T, Sherratt JA, Maini PK. Dictyostelium discoideum: cellular self-organization in an excitable biological medium. Proc R Soc B 1995;259:249–257.
19. Glock P, Brauns F, Halatek J, Frey E, Schwille P. Design of biochemical pattern forming systems from minimal motifs. Elife 2019;8:e48646.
20. Liu J, Prindle A, Humphries J, Gabalda-Sagarra Ma, Asally M, Lee DyD, et al. Metabolic co-dependence gives rise to collective oscillations within biofilms. Nature 2015;523:550–554.
21. Bocci F, Suzuki Y, Lu M, Onuchic JN. Role of metabolic spatiotemporal dynamics in regulating biofilm colony expansion. Proc Natl Acad Sci USA 2018;115:4288–4293.
22. Mikami T, Asally M, Kano T, Ishiguro A. One-dimensional reaction-diffusion model for intra- and inter- biofilm oscillatory dynamics. ALIFE 2020: The 2020 Conference on Artificial Life 1992;9:197–213.
23. Mirams GR, Arthurs CJ, Bernabeu MO, Bordas R, Cooper J, Corrias A, et al. Chaste: an open source C++ library for computational physiology and biology. PLoS Comput Biol 2013;9:e1002970.
24. Osborne JM, Fletcher AG, Pitt-Francis JM, Maini PK, Gavaghan DJ. Comparing individual-based approaches to modelling the self-organization of multicellular tissues. PLOS Comput Biol 2017;13:e1005387.
25. Pathmanathan P, Cooper J, Fletcher A, Mirams G, Murray P, Osborne J, et al. A computational study of discrete mechanical tissue models. Phys Biol 2009;6:036001.
26. Fletcher AG, Osborne JM, Maini PK, Gavaghan DJ. Implementing vertex dynamics models of cell populations in biology within a consistent computational framework. Prog Biophys Mol Biol 2013;113:299–326.
27. Dunn SJ, Näthke IS, Osborne JM. Computational models reveal a passive mechanism for cell migration in the crypt. PLoS ONE 2013;8:e80516.
28. Figueredo GP, Joshi TV, Osborne JM, Byrne HM, Owen MR. On-lattice agent-based simulation of populations of cells within the open-source Chaste framework. Interface Focus 2013;3:20120081.
29. Fisher RA. The wave of advance of advantageous genes. Ann Eugen 1937;7:355–369.
30. Murray JD. Mathematical Biology: I. An Introduction. Springer; 2002.
31. El-Hachem M, McCue SW, Jin W, Du Y, Simpson MJ. Revisiting the Fisher–Kolmogorov–Petrovsky–Piskunov equation to interpret the spreading–extinction dichotomy. Proc R Soc A 2019;475:20190378.
32. Loyinmi AC, Akinfe TK. Exact solutions to the family of Fisher's reaction-diffusion equation using Elzaki homotopy transformation perturbation method. Eng Rep 2020;2:e12084.
33. Ahrens J, Geveci B, Law C. ParaView: An end-user tool for large data visualization. The visualization handbook 2005;717.
34. Li B, Wang F, Zhang X. Analysis on a generalized Sel'kov–Schnakenberg reaction–diffusion system. Nonlin Anal Real World Appl 2018;44:537–558.
35. Schnakenberg J. Simple chemical reaction systems with limit cycle behaviour. J Theor Biol 1979;81:389–400.
36. Al Noufaey K. Semi-analytical solutions of the Schnakenberg model of a reaction-diffusion cell with feedback. Results Phys 2018;9:609–614.
37. Murray JD. Mathematical Biology II: Spatial Models and Biomedical Applications. Springer; 2003.
38. Turing AM. The Chemical Basis of Morphogenesis. Philosophical Transactions of the Royal Society of London Series B, Biological Sciences 1952;237:37–72.
39. Page K, Maini PK, Monk NA. Pattern formation in spatially

heterogeneous Turing reaction–diffusion models. Physica D 2003;181:80–101.
40. Maini PK, Benson DL, Sherratt JA. Pattern formation in reaction-diffusion models with spatially inhomogeneous diffusion coefficients. Math Med Biol 1992;9:197–213.
41. Flint HJ, Scott KP, Duncan SH, Louis P, Forano E. Microbial degradation of complex carbohydrates in the gut. Gut Microbes 2012;3:289–306.
42. Zerfass C, Christie-Oleza J, Soyer O. Manganese oxide biomineralization provides protection against nitrite toxicity in a cell-density-dependent manner. Appl Environ Microbiol 2019;85:e02129–18.
43. Nadell CD, Foster KR, Xavier JB. Emergence of spatial structure in cell groups and the evolution of cooperation. PLoS Comput Biol 2010;6:e1000716.
44. Mitri S, Clarke E, Foster KR. Resource limitation drives spatial organization in microbial groups. ISME J 2016;10:1471–1482.
45. Momeni B, et al. Strong inter-population cooperation leads to partner intermixing in microbial communities. Elife 2013a;2:e00230.
46. Tudge SJ, Watson RA, Brede M. Game theoretic treatments for the differentiation of functional roles in the transition to multicellularity. J Theor Biol 2016;395:161–173.
47. Rubin IN, Doebeli M. Rethinking the evolution of specialization: A model for the evolution of phenotypic heterogeneity. J Theor Biol 2017;435:248–264.
48. Varahan S, Walvekar A, Sinha V, Krishna S, Laxman S. Metabolic constraints drive self-organization of specialized cell groups. eLife 2019;8:e46735.
49. Weijer C. Collective cell migration in development. J Cell Sci 2009;122:3215–3223.
50. Johnson CGM, Fletcher AG, Soyer OS. ChemChaste. https://githubcom/OSS-Lab/ChemChaste Accessed on 29/04/2022;.
51. Johnson CGM, Fletcher AG, Soyer OS. Supporting data for "ChemChaste: Simulating spatially inhomogenous biochemical reaction-diffusion systems for modelling cell-environment feedbacks" . GigaScience Database 2022;http://dx.doi.org/10.5524/102218.

Click here to access/download
Supplementary Material
ChemChaste_Supplementary_Information_Revised_Line_Numbers.pdf
