# Supplementary material for: ChemChaste: Simulating spatially inhomogeneous biochemical reaction–diffusion systems for modeling cell–environment feedbacks
Source: Gigascience. 2022 Jun 17;11:giac051. doi: 10.1093/gigascience/giac051 (PMC9205757; doi:10.1093/gigascience/giac051)
Supplement: giac051_Supplemental_File [file giac051_supplemental_file.pdf]

Supporting information for: ChemChaste: Simulating spatially inhomogenous biochemical reaction-diffusion systems for modelling cell-environment feedbacks

Connah G. M. Johnson[1,2], Alexander G. Fletcher[3,4,*], Orkun S. Soyer[2,*]

[1] Mathematics of Real-World Systems Doctoral Training Centre, University of Warwick, Coventry, UK
[2] School of Life Sciences, University of Warwick, Coventry, UK
[3] School of Mathematics & Statistics, University of Sheffield, Sheffield, UK
[4] Bateson Centre, University of Sheffield, Sheffield, UK

* Corresponding author(s): O.Soyer@warwick.ac.uk, a.g.fletcher@sheffield.ac.uk

S1 Brief introduction to the finite element method and the hybrid continuum-discrete model used in ChemChaste

At the heart of ChemChaste lies a suite of finite element (FE) solvers, which are used to numerically solve systems of reaction-diffusion partial differential equations (PDEs). These equations track the spatiotemporal dynamics of a set C of chemical species over a bounded rectangular domain $\Omega \subset \mathbb{R}^2$ whose boundary is denoted $\partial\Omega$. Each chemical species $c \in C$ is associated with a scalar concentration field, with the associated state variable $u_c(\mathbf{x}, t) \in \mathbb{R}$ denoting the concentration of the chemical at position $\mathbf{x} \in \Omega$ at time t.

ChemChaste couples the reaction-diffusion system to an agent-based cell system to model a spatially distributed cell population. We define a set of cells, $p \in P$, within the domain which are modelled as point sources at position $\mathbf{x}_p \in \Omega$. These cells perform reactions independently of the domain reactions and exchange molecules with the domain. These exchanges are described by the transport law $T(\mathbf{u}, t) : {\mathbb{R}_+}^{|C|} \times [0, t) \to \mathbb{R}^{|C|}$ controlling the flux of molecules between the bulk and the cell.

During a simulation the vector of state variables, $\mathbf{u} = \mathbb{R}^{|C|}$, evolves through the parabolic PDE system

$$\frac{\partial \mathbf{u}}{\partial t} - \nabla \cdot [D(\mathbf{x}) \cdot \nabla \mathbf{u}] = R(\mathbf{x}, \mathbf{u}, t) + \sum_{p \in P} T_p(\mathbf{u}, t)\delta(\mathbf{x} - \mathbf{x}_p), \tag{1}$$

where $T_p(\mathbf{u}, t)$ is the source/sink contribution by the cell p located at position $\mathbf{x}_p$, $R(\mathbf{x}, \mathbf{u}, t) : \Omega \times {\mathbb{R}_+}^{|C|} \times [0, t) \to \mathbb{R}^{|C|}$ is the reaction ordinary differential equation (ODE) system defined over the domain, and δ is the Dirac delta distribution. ChemChaste models the spatially discontinuous distribution by a top-hat distribution with maximum value 1 and 2D extent covering the Gauss point associated with location $\mathbf{x}$ (see section S1.2). We solve equation (1) as an initial boundary value problem (IBVP) with given initial conditions (ICs) and boundary conditions (BCs).

For each chemical species, we allow for either fixed-value Dirichlet BCs of the form

$$u_c = a \;\text{ for }\; \mathbf{x} \in \partial\Omega, \tag{2}$$

or fixed-flux Neumann BCs of the form

$$-D_c(\mathbf{x}) \nabla u_c \cdot \mathbf{n} = b_c \;\text{ for }\; \mathbf{x} \in \partial\Omega, \tag{3}$$

where $\mathbf{n}$ is the unit outward normal, $D_c(\mathbf{x})$ is the (*local*) diffusion coefficient of chemical species c, and $a, b \in \mathbb{R}$ are constants. Isolated conditions, $b_c = 0$, are assumed for chemicals without a user defined Neumann BC.

While the BCs are defined on the whole boundary ChemChaste handles chemical initial conditions through labelling regions of the domain. A set of regions, S, are provided using the

file based input (see section S2.2), which link to chemical concentrations. Let $s \in S$ label a region $\Omega_s \subseteq \Omega$ associated with an initial chemical concentration vector $\mathbf{u}_0^s \in \mathbb{R}_+^{|C|}$. For each position $\mathbf{x} \in \Omega$, the initial conditions are implemented with sharp discontinuous boundaries

$$\mathbf{u}_0(\mathbf{x}) = \sum_{s \in S} \mathbf{u}_0^s \, \mathbb{1}_{\Omega_s}(\mathbf{x}) \tag{4}$$

where

$$\mathbb{1}_{\Omega_s}(\mathbf{x}) = \begin{cases} 1 & \text{if } \mathbf{x} \in \Omega_s \\ 0 & \text{if } \mathbf{x} \notin \Omega_s \end{cases}$$

is the indicator function.

We solve the above system numerically using a FE method, which divides the spatial domain Ω into a discrete mesh comprising nodes and elements, and converts the PDE problem into a coupled system of algebraic equations (Pathmanathan, 2012).

S1.1 Finite element method for a reaction-diffusion PDE coupled to a cell mesh

To apply the FE method to the PDE system (1), we first derive the weak formulation of the problem. We start by discretizing time into discrete timesteps $t^m = m\Delta t$ and applying a semi-implicit scheme to the time derivative in (1), obtaining

$$\frac{1}{\Delta t}\mathbf{u}^{m+1} - \frac{1}{\Delta t}\mathbf{u}^m - \nabla \cdot [D(\mathbf{x}) \cdot \nabla \mathbf{u}^{m+1}] = R(\mathbf{x}, \mathbf{u}^m, t^m) + \sum_{p \in P} T_p(\mathbf{u}^m, t^m)\delta(\mathbf{x} - \mathbf{x}_p), \tag{5}$$

where $\mathbf{u}^m$ denotes the approximation to the chemical concentration vector $\mathbf{u}(\mathbf{x}, t^m)$ for $\mathbf{x} \in \Omega$. Next, we multiply by a vector $\mathbf{v}$ of arbitrary 'test functions' with each element $v \in \mathbf{v}$ defined in a function subspace of the Sobolev space $v \in \hat{V} \subset H_0^1(\Omega)$ and integrate over the domain Ω. Rearranging, we obtain

$$\begin{aligned} 0 = \frac{1}{\Delta t}\int_\Omega \mathbf{u}^{m+1} \cdot \mathbf{v} \,\mathrm{d}V - \frac{1}{\Delta t}\int_\Omega \mathbf{u}^{m} \cdot \mathbf{v} \,\mathrm{d}V - \int_\Omega \nabla \cdot [D(\mathbf{x}) \cdot \nabla \mathbf{u}^{m+1}] \cdot \mathbf{v} \,\mathrm{d}V \\ - \int_\Omega [R(\mathbf{x}, \mathbf{u}^m, t^m) + \sum_{p \in P} T_p(\mathbf{u}^m, t^m)\delta(\mathbf{x} - \mathbf{x}_p)] \cdot \mathbf{v} \,\mathrm{d}V. \end{aligned}$$

Writing the diffusion term as $\nabla \cdot [D(\mathbf{x}) \cdot \nabla \mathbf{u}^{m+1}]\mathbf{v} = \nabla \cdot [\mathbf{v} \cdot D(\mathbf{x}) \cdot \nabla \mathbf{u}^{m+1}] - D(\mathbf{x}) \cdot \nabla \mathbf{v} \cdot \nabla \mathbf{u}^{m+1}$, and applying the divergence theorem, we obtain

$$\begin{aligned} 0 = \frac{1}{\Delta t}\int_\Omega \mathbf{u}^{m+1} \cdot \mathbf{v} \,\mathrm{d}V - \frac{1}{\Delta t}\int_\Omega \mathbf{u}^{m} \cdot \mathbf{v} \,\mathrm{d}V + \int_\Omega D(\mathbf{x}) \cdot \nabla \mathbf{v} \cdot \nabla \mathbf{u}^{m+1} \,\mathrm{d}V - \int_{\partial\Omega} [\mathbf{v} \cdot D(\mathbf{x}) \cdot \nabla \mathbf{u}^{m+1}] \cdot \hat{n} \,\mathrm{d}S \\ - \int_\Omega [R(\mathbf{x}, \mathbf{u}^m, m\Delta t) + \sum_{p \in P} T_p(\mathbf{u}^m, m\Delta t)\delta(\mathbf{x} - \mathbf{x}_p)] \cdot \mathbf{v} \,\mathrm{d}V. \end{aligned}$$

We then consider the BCs as $v = 0$, $\forall v \in \mathbf{v}$ by the natural boundary definition for Dirichlet types and $\nabla u_c \cdot \hat{n} = \mathbf{b}$ for the Neumann types to get

$$\begin{aligned} 0 = \frac{1}{\Delta t}\int_\Omega \mathbf{u}^{m+1} \cdot \mathbf{v} \,\mathrm{d}V - \frac{1}{\Delta t}\int_\Omega \mathbf{u}^{m} \cdot \mathbf{v} \,\mathrm{d}V + \int_\Omega D(\mathbf{x}) \cdot \nabla \mathbf{v} \cdot \nabla u^{m+1} \,\mathrm{d}V - \int_{\partial\Omega} \mathbf{b} \circ \mathbf{v} \cdot D(\mathbf{x}) \,\mathrm{d}S \\ - \int_\Omega [R(\mathbf{x}, \mathbf{u}^m, m\Delta t) + \sum_{p \in P} T_p(\mathbf{u}^m, m\Delta t)\delta(\mathbf{x} - \mathbf{x}_p)] \cdot \mathbf{v} \,\mathrm{d}V \end{aligned} \tag{6}$$

which defines the weak formulation of the PDE as a variational problem, with $\circ$ representing the element-wise product. This allow us to determine functions $\mathbf{v}$ in order to find the next

state variable vector $\mathbf{u}^{m+1}$ given the previous $\mathbf{u}^m$ state variable vector such that the remaining Dirichlet BCs for $\mathbf{u}^{m+1}$ are satisfied (Logg *et al.*, 2012). This problem is solved on the nodes of a discretised spatial domain, i.e the FE mesh nodes.

To implement the FE method a mesh is placed over the domain, Ω. This mesh provides a space of discrete nodes and for each node i at position n_i on the domain $\Omega \setminus \partial\Omega$, i.e each node not on the boundary, a set of linear basis functions ϕ_i are defined $\{\phi_1, \phi_2, ..., \Phi_N\}$ where N is the number of nodes in the domain. We solve equation (6) on the discrete node points and interpolate onto a fine rectilinear grid to approximate the domain locations $\mathbf{x}$. We refer to these interpolated locations as Gauss points and discuss the interpolation in Section S1.2. The Gauss point $\mathbf{x}$ is given by the product of the node location $\mathbf{n}_j$ and the linear test basis function ϕ_j;

$$\mathbf{x} = \sum_{j=1}^{N} \mathbf{n}_j \phi_j \tag{7}$$

Using the mesh interpolation the trial function can be approximated by

$$\mathbf{u}^m = \sum_{j=1}^{N} U_j^m \phi_j \tag{8}$$

where $\mathbf{U}_j$ is the nodal value. Additionally, we find the gradient of the trial function is given by

$$\nabla \mathbf{u}^{m+1} = \sum_{j=1}^{N} \mathbf{U}_j^m \nabla \phi_j \tag{9}$$

We are free to restrict the basis functions, ϕ_i, to functions that satisfy the requirements for the test functions over the Ω. Therefore we may substitute $\boldsymbol{\phi}_i$ for $\mathbf{v}$

$$\begin{aligned}
0 = {} & \frac{1}{\Delta t}\int_\Omega \sum_{j=1}^{N} \mathbf{U}_j^{m+1}\phi_j \cdot \boldsymbol{\phi}_i \,\mathrm{d}V - \frac{1}{\Delta t}\int_\Omega \sum_{j=1}^{N} \mathbf{U}_j^{m}\phi_j \cdot \boldsymbol{\phi}_i \,\mathrm{d}V \\
& + \int_\Omega D(\sum_{j=1}^{N}\mathbf{n}_j\phi_j) \cdot \nabla\boldsymbol{\phi}_i \cdot \sum_{j=1}^{N}\mathbf{U}_j^{m+1}\nabla\phi_j \,\mathrm{d}V - \int_{\partial\Omega} \boldsymbol{\phi}_i \cdot D(\sum_{j=1}^{N}\mathbf{n}_j\phi_j) \circ \mathbf{b}\,\mathrm{d}S \\
& - \int_\Omega [R(\sum_{j=1}^{N}\mathbf{n}_j\phi_j, \sum_{j=1}^{N}\mathbf{U}_j^m\phi_j, m\Delta t) + \sum_{p\in P} T_p(\sum_{j=1}^{N}\mathbf{U}_j^m\phi_j, m\Delta t)\delta(\sum_{j=1}^{N}\mathbf{n}_j\phi_j - \mathbf{x}_p)] \cdot \boldsymbol{\phi}_i \,\mathrm{d}V
\end{aligned}$$

which can be converted into the algebraic system

$$\left(\frac{1}{\Delta t}M + K\right)\mathbf{U}^{m+1} = \frac{1}{\Delta t}M\mathbf{U}^m + \mathbf{B} \tag{10}$$

by defining K, M and $\mathbf{B}$, as

$$K_{ij} = \int_\Omega D(\sum_{j=1}^{N}\mathbf{n}_j\phi_j) \cdot \nabla\boldsymbol{\phi}_i \cdot \nabla\phi_j \,\mathrm{d}V$$

$$M_{ij} = \int_\Omega \boldsymbol{\phi}_i\phi_j \,\mathrm{d}V$$

and

$$\begin{aligned}
\mathbf{B}_i = {} & \int_{\partial\Omega} \boldsymbol{\phi}_i \cdot D(\sum_{j=1}^{N}\mathbf{n}_j\phi_j) \circ \mathbf{b}\,\mathrm{d}S \\
& + \int_\Omega [R(\sum_{j=1}^{N}\mathbf{n}_j\phi_j, \sum_{j=1}^{N}\mathbf{U}_j^m\phi_j, m\Delta t) - \sum_{p\in P} T_p(\sum_{j=1}^{N}\mathbf{U}_j^m\phi_j, m\Delta t)\delta(\sum_{j=1}^{N}\mathbf{n}_j\phi_j - \mathbf{x}_p)] \cdot \boldsymbol{\phi}_i \,\mathrm{d}V
\end{aligned}$$

Dirichlet BCs are applied by altering the matrix, K, and vector, B_i, for the contributions of nodes on $\partial\Omega$ while Neumann BCs are contained within the definition of B_i. The coupled cell system is performed by solving a system of ODEs and agent properties for the cells and solving the linear system (Equation (10)).

S1.2 Interpolation of the nodal solution to Gauss points and coupling to cells

As motivated in Section S1.1, the FE method employed discretises the domain space using elements formed by nodes, solves a linear algebraic system on those nodes and then interpolates the solution within each element. While Chaste has been developed to run simulations in 1, 2, or 3 spatial dimensions, the current version of ChemChaste has been restricted to simulate 2-dimensional domains. Chaste utilises linear Lagrange elements for creating the FE mesh (Logg *et al.*, 2012). To construct the FE mesh for a 2-dimensional domain the domain is partitioned into a finite set of triangles, $\mathbf{T}$, which cover the space;

$$\Omega = \cup_{T\in\mathbf{T}} T$$

Each triangle contains a triplet of nodes where a pair of nodes may be shared by adjacent triangles, shown in Figure S1a. The mesh is defined by the set of N nodes, $L = \{l_1, l_2, ..., l_N\}$.

This triangulation process and the resulting elements define a so-called Sobolev space, and permits the choice of a linear basis, ϕ, for the test functions (Shapira, 2012). In Chaste, the linear Lagrange basis is used such that for node triplet at triangle locations $\{\tilde{n}_x, \tilde{n}_y, \tilde{n}_z\} = \{(0,0), (1,0), (0,1)\}$ is spanned by a basis of the form;

$$\begin{aligned}\phi_x(\mathbf{x}) &= 1 - x_1 - x_2\\ \phi_y(\mathbf{x}) &= x_1\\ \phi_z(\mathbf{x}) &= x_2\end{aligned}$$

where the test function is approximated by the linear superposition of the values at the nodes

$$\mathbf{v} = \sum_{i\in\{x,y,z\}} \mathbf{v}(\tilde{n}_i)\phi_i$$

The positions within the triangle are discretised by a fine grid for computational purposes. These grid locations $\mathbf{x} = \sum_{i\in\{x,y,z\}} \tilde{n}_i\phi_i$ are known as the Gauss points. The local points are mapped to global locations as shown in Figure S1a. ChemChaste introduces the cell contribution to equation (10) if a cell is located at the Gauss point, Figure S1b.

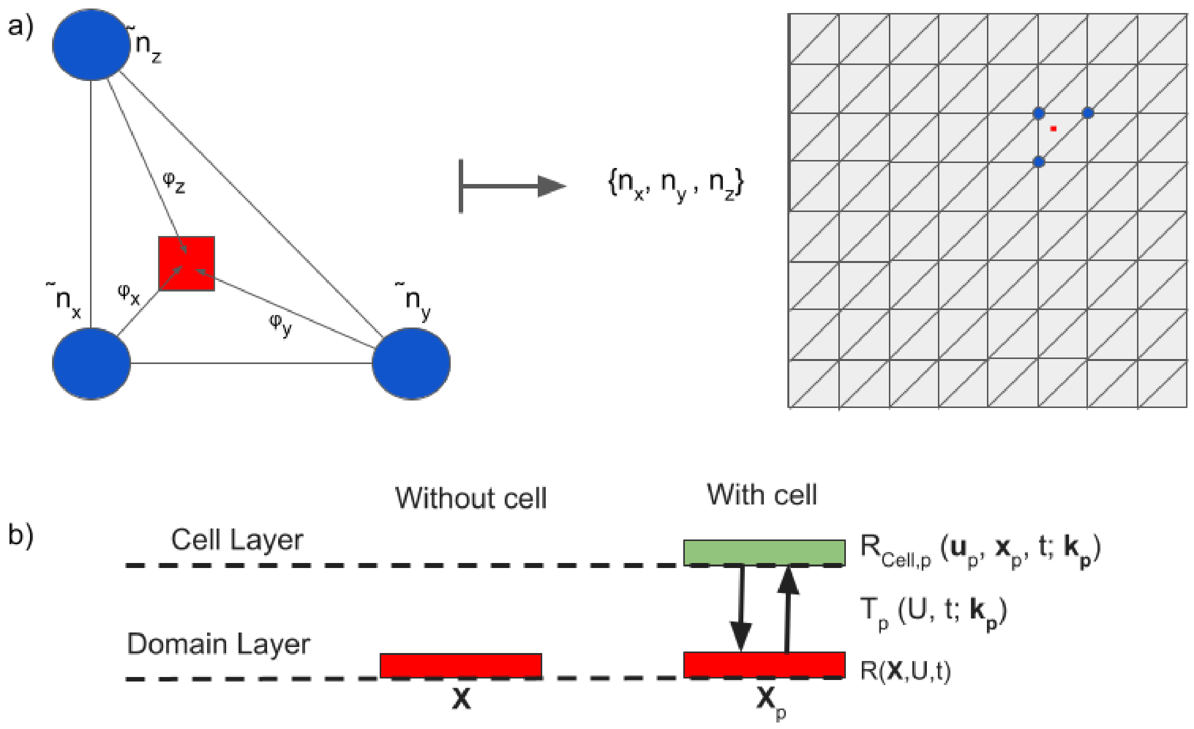

Figure S1: Cartoon showing the interpolation procedure from finite triangle elements to Gauss point and coupling to cell agents. a) The nodes (blue) of the triangle are located at positions $\tilde{n}_i$ and each node contributes to the particular Gauss point (red square) through the node's basis function ϕ_i for node $i \in \{x, y, z\}$. The node locations and other interpolated quantities are mapped from the local reference triangle to the global mesh i.e by applying a mapping function to the node position $M(\tilde{n}_i) \to n_i$, which may, in general, rotate and stretch the reference triangle. b) Cells have a point location which share the location of a Gauss point. Each point in Ω is associated with a spatially dependent reaction system, $R(\mathbf{x}, U, t)$ and may also be associated with a cell. These cells (green) contain their own reaction system, $R_{Cell,p}(\mathbf{u}_p, \mathbf{x}_p, t; \mathbf{k}_p)$, and are coupled to the domain through a transport law $T_p(U, t; \mathbf{k}_p)$.

S1.3 Simulations of chemical reaction PDEs without cells: Chemical reactions $R(\mathbf{u})$

Reaction-diffusion simulations in ChemChaste are created from a set of user-defined chemical reactions as provided in configuration files. Let the set of all chemical species within a system be denoted by C and the concentration of a particular species $c \in C$ be denoted u_c where $u_c \in \mathbb{R}_{\geq 0}$. For a given reaction, let $\alpha_c, \beta_c \in \mathbb{N}$ denote the unsigned stoichiometry of species c. $\beta >= 1$ if the species is a product of the reaction, where β_c is the number of the species produced per one instance of the reaction. Conversely, α_c is the number of the species consumed within the reaction; $\alpha >= 1$ if the species is a substrate species of the reaction. The change in concentration, hence value for u_c, is given by

$$\frac{du_c}{dt} = (\beta_c - \alpha_c)R(\mathbf{u}),$$

where the function $R(\mathbf{u})$ defines the reaction dynamics (Table S1).

For a given reaction, let $S \subseteq C$ denote the set of substrates in the reaction, $P \subseteq C$ the set of products, kf the forward reaction rate constant, kr the reverse reaction rate constant, and let $\tilde{S} \subseteq \{c \in C | \beta = 0, \alpha = 0\}$ be the set of spectator species. The spectator species affect the reaction rate while remaining unchanged themselves. As an examplar, consider a set of chemicals

in the domain $C = \{A, B, C, D, E, F\}$ and a chemical reaction involving two substrate species $S = \{A, B\}$, two products $P = \{C, D\}$ and a single spectator species $\tilde{S} = E$. The chemical reaction may be written as;

$$\alpha_{\mathrm{A}}\mathrm{A} + \alpha_{\mathrm{B}}\mathrm{B} \longrightarrow \beta_{\mathrm{C}}\mathrm{C} + \beta_{\mathrm{D}}\mathrm{D}$$

where the change in concentrations will depend on $R(\mathbf{u})$;

$$\begin{aligned}
\frac{du_A}{dt} &= -\alpha_A R(\mathbf{u}) \\
\frac{du_B}{dt} &= -\alpha_B R(\mathbf{u}) \\
\frac{du_C}{dt} &= \beta_C R(\mathbf{u}) \\
\frac{du_D}{dt} &= \beta_D R(\mathbf{u}) \\
\frac{du_E}{dt} &= 0 \\
\frac{du_F}{dt} &= 0
\end{aligned}$$

E is a spectator species and therefore is not produced or consumed in the reactions but affects the value for $R(\mathbf{u})$ and $\frac{du_E}{dt} = 0$ as E as a steady state for E is assumed within the context of this reaction system. Implicitly, $\frac{du_F}{dt} = 0$ as F as chemical F is present within the domain but is not involved within the reaction system.

Table S1 presents the pre-implemented reaction rate laws for bulk domain reactions. Each reaction law is supplied with a chemical equation and a set of parameters and ChemChaste calculates the reaction rate, $R(\mathbf{u})$.

Reaction name	Reversible	Reaction rate formula $R(\mathbf{u})$
`ZerothOrderReaction`	False	kf
`ZerothOrderReversibleReaction`	True	$kf - kr$
`MassActionReaction`	True	$kf \prod_{c \in P} u_c^{\alpha_c} - kr \prod_{c \in S} u_c^{\beta_c}$
`SpectatorDependentReaction`	False	$kf \prod_{c \in \tilde{S}} u_c$
`MichaelisMentenReaction`	False	$k_{cat} u_E \prod_{c \in S} u_c^{\beta_c} / (K_M + \prod_{c \in S} u_c^{\beta_c})$

Table S1: Reaction rates currently implemented in ChemChaste. The rates include constants k_f, k_r, k_{cat}, K_M and labels for spectator chemicals.For the reversible reactions, the rates may be negative $R(\mathbf{u}) \in \mathbb{R}$ implying the reaction occurs in the reverse direction while irreversible reaction the rate must be positive $R(\mathbf{u}) \in \mathbb{R}_{\geq 0}$ The user may implement their own reactions rates building upon these forms by adding a new reaction file to the inheritance structure of ChemChaste (Section S3).

We use an inheritance strategy to readily build more complex reactions into ChemChaste, Figure S2. Therefore the functionality of the base reaction, `ZerothOrderReaction`, is inherited by all the upstream classes. The reactions currently implemented are considered foundational as they focus on different reaction properties (i.e reversibility, rate dependent on spectator species etc.) and may be easily built upon by a user to combine these properties under different rate laws.

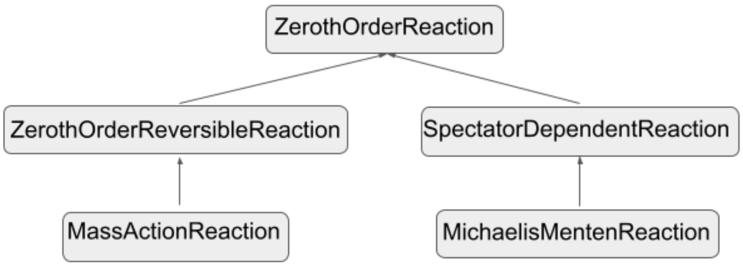

Figure S2: The inheritance structure for the bulk domain reaction files currently implemented in ChemChaste. These reactions are shown in Table S1. The structure builds from the base reactions, `ZerothOrderReaction`, to add more complex reaction rate laws.

S1.4 Simulations of coupled cell-domain systems: transport, $T(\mathbf{u}, \mathbf{u}')$, and membrane reactions, $M(\mathbf{u}, \mathbf{u}')$

Simulations that couple a cell mesh to the reaction-diffusion domain utilise three different reaction types; chemical reactions (Table S1), transport reactions (Table S2), and membrane reactions (Table S3). The necessary file structure to call these reaction systems is given in Figure S9a. The transport reactions model chemical transport across the membrane and directly couple the cells to the domain, see Figure S1. Let the set of transported chemicals be denoted by C and a single chemical by c where $c \in C$. Let C_{domain} denote the set of chemicals in the transport process located in the domain external to the cell membrane and C_{cell} be the set of cellular chemicals. As the point $x \in \Omega$ is associated with both the domain and the cell there are two concentration vectors tied to the point, $\mathbf{u}$ for the chemical concentrations in the domain and $\mathbf{u}'$ for the concentrations within the cell. The lengths of these two vectors need not be the same, as in general $|C_{domain}| \neq |C_{cell}|$.

Let the rate of the transport process connecting the two concentration vectors be denoted by $T(\mathbf{u}, \mathbf{u}')$

$$\frac{du_c^{cell}}{dt} = -(\beta_c - \alpha_c)T(\mathbf{u}, \mathbf{u}')$$
$$\frac{du_c^{domain}}{dt} = (\beta_c - \alpha_c)T(\mathbf{u}, \mathbf{u}')$$

where α_c, $\beta_c \in \mathbb{N}$ are the stoichiometric coefficients for chemical $c \in C$ when considering the transport process as a "reaction". Here α is the set of coefficients for the chemicals on the domain side of the membrane, that is the quantity of each species consumed in the forward sense of the process (i.e cell uptake), and β is the set of coefficients for the chemicals on the cell side of the process, (i.e cell excretion). For example, consider the reaction occurring at a rate $T(\mathbf{u}, \mathbf{u}')$;

$$\alpha \mathrm{A} \rightleftharpoons \beta A'$$

where α denotes the amount of A in the domain that are consumed in the forward reaction to produce β of A' within the cell.

Transport process name	Reversible	Transport rate $T(\mathbf{u},\mathbf{u}')$
`ZerothOrderTransportIntoCell`	False	kf
`ZerothOrderTransportOutOfCell`	False	kr
`ZerothOrderReversibleTransport`	True	$kf - kr$
`MassActionTransportReaction`	True	$kf \prod_{c \in C_{cell}} u_c^{\alpha_c} - kr \prod_{c \in C_{domain}} {u'}_c^{\beta_c}$

Table S2: The foundational transport process types implemented at present, including whether a process is reversible and the functional rate law utilised. The user may implement their own laws by adding a new transport reaction file to the ChemChaste system (Section S4).

The rate constants are defined with appropriate units, such that the units for the transport process are given in 'concentration per unit area per unit time', i.e the amount of substance passing through the membrane in an infinitesimal length of time.

For reactions defined at the membrane, two separate reactions occurring on either side of the cell membrane, i.e. inside and outside, are coupled. Such reactions do not result in any transfer of chemicals across the cell boundary, but they alter the concentrations of species in the domain and inside the cell. The membrane reactions use two sets of stoichiometic coefficients. Let (α, β) be the stoichiometric coefficients for the reaction internal to the cell and (α', β') be the coefficients for the external domain reaction. As before, we denote the membrane reaction rate denoted by $M(\mathbf{u},\mathbf{u}')$;

$$\frac{du_c}{dt} = (\beta_c - \alpha_c)M(\mathbf{u},\mathbf{u}')$$
$$\frac{du'_c}{dt} = (\beta'_c - \alpha'_c)M(\mathbf{u},\mathbf{u}')$$

where variables and parameters take on the meaning defined previously. For two general bi-molecular reaction systems coupled at the membrane, the system takes the form;

$$\alpha_{\mathrm{A}}\mathrm{A} + \alpha_{\mathrm{B}}\mathrm{B} \rightleftharpoons \beta_{\mathrm{C}}\mathrm{C} + \beta_{\mathrm{D}}\mathrm{D}$$
$$\alpha'_E\mathrm{E} + \alpha'_F\mathrm{F} \rightleftharpoons \beta'_G\mathrm{G} + \beta'_H\mathrm{H}$$

where the first and second reaction occur in the domain and inside the cell, respectively. The concentrations are provided by the separate state vectors as in Section S1.3 but with a shared rate, $M(\mathbf{u},\mathbf{u}')$, which depends on both state vectors.

Membrane reaction name	Reversible	Membrane reaction rate $M(\mathbf{u},\mathbf{u}')$
ZerothOrderCoupledMembrane	False	kf
ZerothOrderReversibleMembrane	True	$kf - kr$
MassActionCoupledMembraneReaction	True	$kf \prod_{i \in C_{cell}} {u'}_i^{{\alpha'}_i} \prod_{j \in C_{domain}} u_j^{\alpha_j} - kr \prod_{i \in C_{cell}} {u'}_i^{{\beta'}_i} \prod_{j \in C_{domain}} u_j^{\beta_j}$

Table S3: The membrane reaction types implemented at present, including whether a reaction is reversible and the functional rate law utilised. The user may implement their own membrane reaction laws by adding a new reaction file to the ChemChaste system (Section S5).

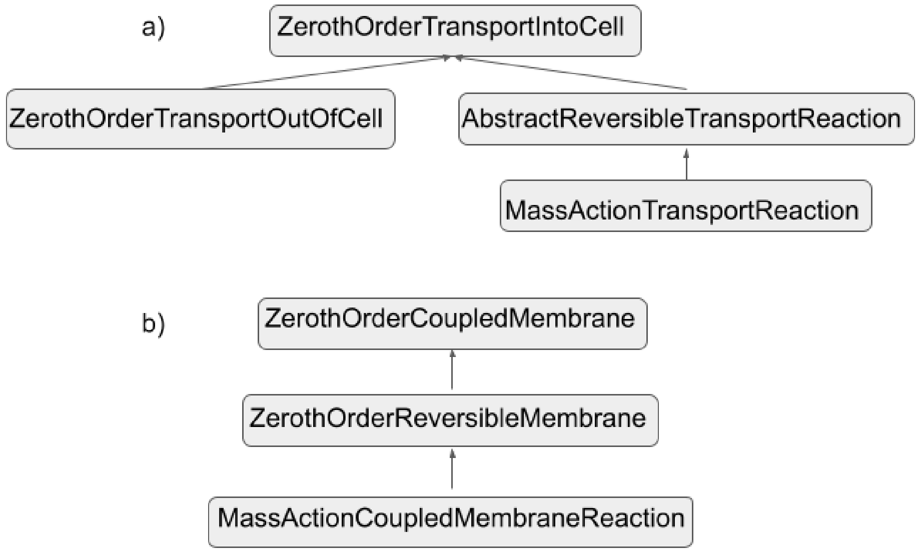

Figure S3: The inheritance structure for the transport a) and membrane b) reaction types currently implemented in ChemChaste. These reactions are shown in Table S2 and Table S3 respectively. a) The transport reactions build upon `ZerothOrderTransportIntoCell` as a base while in b) the membrane reactions build upon `ZerothOrderCoupledMembrane`. These laws may be built upon to add more complex transport and membrane reaction rates. The user may write their own transport processes and membrane rate laws by following the file structure given in Sections S4–S5.

S2 Setting up a ChemChaste simulation with user-defined model properties and parameters

ChemChaste has been developed with the user in mind and simulations are defined in three steps. (i) The overall simulation parameters (Table S4) and file paths are specified in a configuration file; example configuration files are given in Figures S5 and S8. (ii) The reaction-diffusion system in the bulk is defined. The name of the directory is specified within the previous simulation configuration file (see Figure S5b). The reaction system (Table S1) and bulk domain information are stored in TXT and CSV files; exemplars are given in Figures S7 and S6 respectively. (iii) If coupling to a discrete cell-based model, the cell population and individual cell type properties are specified. The cell model information is given a separate directory within the simulation directory, as shown in Figure S9 for a two cell type example. For each cell type given in the TXT files, a cell-based reaction system is defined (Table S1) with cell-specific transport laws (Table S2) and membrane bound reactions (Table S3); example cell reaction files are given in Figure S10. The topology of the cell layer, i.e. the initial location of cells at the start of a simulation, is supplied by a CSV file together with a key file translating the cell ID used in the topology file to the names of the cell types (which are also used in the file directories for cell files).

In the following sections, we explain each of the required configuration files and their contents to run a ChemChaste simulation. These configuration files are parsed and fed into a C++ ChemChaste simulation through the help of a "run-script".

S2.1 ChemChaste run script

To run a ChemChaste simulation, users can make use of a "run-script" file, which is a command line script written in Phyton 3 language (see example in Figure S4). A new "run-script" file should be supplied for each simulation, although simulations with same files but different parameters, e.g. for parameter 'sweeping', can be provided through the same "run-script" file (see example). The run-script sets the ChemChaste executable to run in naive parallel, that is one simulation per processor, and runs the testing and compilation features of Chaste. Within the "run-script" file the user defines the relative directories for the different configuration files, as well as some of the global simulation parameters. The full set of parameters that can be set in the "run-script" file are given S4. Here, the configuration files for the Cross feeding (Section S2.3) and Schnackenberg spatial patterning (Section S2.2) are used as examples. In the example file provided, a total of 10 simulations are executed, 5 of type `complex_cell` and 5 of type `domain_only`, and distributed over 3 cores. Some of these parameters can also be set in other configuration files, as discussed below.

a)

```python
# This script generates and submits jobs for a parameter sweep
import multiprocessing
import subprocess
from ChemChasteDefinitions import *
```

b)

```python
# generate a list of bash commands
command_list = []
# config files for each of the simulations
configCrossfeeding = "/home/chaste/projects/ChemChaste/DataInput/ChemChasteConfigCrossFeedingEnzyme.txt"
configPatterning = "/home/chaste/projects/ChemChaste/DataInput/ChemChasteConfigSchnackenberg.txt"
```

c)

```python
timesteps = [1,0.1,0.01,0.001,0.0001]
simulation_id = 1
```

d)

```python
for dt in timesteps:
    simulationExecutable = str(determineExecutable(configCrossfeeding))
    command = simulationExecutable + str(simulation_id)
    # add config
    command +=  " --config="+configCrossfeeding
    # add simulation type (default "coupled_cell")
    command += " --simulation_type=complex_cell"
    # add additional commands to override config
    command += " --simulation_timestep="+str(dt)
    command += " --sampling_timestep=1e-1"

    # add simulation to the list
    command_list.append(command)
```

e)

```python
for dt in timesteps:
    simulationExecutable = str(determineExecutable(configPatterning))
    command = simulationExecutable + str(simulation_id)
    # add config
    command +=  " --config="+configPatterning
    # add simulation type (default "coupled_cell")
    command += " --simulation_type=domain_only"
    # add additional commands to override config
    command += " --simulation_timestep="+str(dt)
    command += " --sampling_timestep=1e-1"

    # add simulation to the list
    command_list.append(command)
```

f)

```python
# use `count' no of processes
count = 3 # use multiprocessing.cpu_count() for the number of cores on your machine
# generate a pool of workers
pool = multiprocessing.Pool(processes=count)
# ... and pass the list of bash commands to the pool
pool.map(execute_command, command_list)
```

Figure S4: Example "run-script" file for defining features of ChemChaste simulations. The structure of this "run-script" file is such that it is divided into sections, as shown with a letter-based labelling on the figure and as explained next. a) This section defines the import files used for running parallel simulations and controlling the simulation compilation and implementation. b) This section defines the configuration files for each user simulation that are contained within the *DataInput* directory of ChemChaste. c) This section defines the global simulation parameters, in particular those that are used for parameter sweeping. The simulation ID created in this section is shared across a set of simulations to be run in parallel. For these simulations, a different parameter value is to be used - in this example the "time step size" parameter. d) This section defines the command to be used in the command-line initiation of a simulation. The command is created in a series of steps, by appending different aspects of the simulation command together. First the simulation executable, in this case the cross feeding simulation, is created and then appended the simulation type. Then, the desired simulation parameters are amended to the command. In this case, note that the simulation timestep is set by grabbing its value from the provided parameter list and by making use of a for loop structure. Finally the destination for the data file is appended to the command using the parameter `sampling_rate` and a value of $1e^{-1}$. e) This section is a repeat of section d) but using a reaction only simulation example. The Schnackenberg simulation is used with the same parameters as in d) but where `simulation_type` is set to `domain_only`. f) This section defines final aspects of simulations, such as number of processor cores. The parallel simulations are mapped to `count` number of processor cores.

Parameter	Default value	Description
`simulation_type`	`coupled_cell`	String keyword for type of simulation to run
`simulation_timestep`	1/120	Timestep for simulation solvers, Δt_{sim}.
`sampling_timestep`	1/120	Timestep for writing simulation results, $\Delta t_{sampling} \geq \Delta t_{sim}$.
`output_filename`	`ChemChasteExecutable`	Where to write simulation results
`simulation_end_time`	10.0	The maximum timestep value for the simulation.
`number_cells_across`	1	The rectangular cell mesh width, for when a cell domain file is not used.
`number_cells_high`	1	The rectangular cell mesh length, for when a cell domain file is not used.
`number_of_reaction_pdes`	1	Number of RD state variables
`spatial_dimensions`	2	The spatial dimension of the computational domain.
`FE_element_dimensions`	2	The dimensions of the elements used in the finite element (FE) method.
`node_cuttoff_length`	1.5	The cutoff length to label two cells in the cell mesh as interacting, used to set the forces between the cells.
`cell_mesh_origin`	-4.0	The origin location of the cell mesh with respect to the domain FE mesh.
`linear_force_cutoff`	1.5	The force constant for the linear Hookean spring force between cells.

Table S4: ChemChaste simulation parameters that can be set via the "run-script" file. These parameters control the type of simulation to run, the solver properties such as end time and solver time step, and finite element properties such as spatial domain dimensions and element dimensions for the FE implementation. Each parameter is set by name in the simulation configuration file, an example is provided in Figure S5a.

Among the different parameters that can be set in the "run-script" file, and listed in S4, we highlight here some of the key ones. The `simulation_type` parameter, which sets the solver methods used in ChemChaste. This parameter may be specified in either the configuration file or in the run-script (Figures S5 and S4). The parameter options; `domain_only`, `coupled_cell`, `complex_cell`, control how the ChemChaste system builds the simulations. The `domain_only` option is used to solve a reaction-diffusion system in a domain without cells (removing the summation term from equation (1)), while the coupled cell-domain simulations are simulated using either the `coupled_cell` model and the `complex_cell` model. These two models share the same parameter sets and file systems, see Table S4 and Figure S9, but differ in the cell division implementation. The `coupled_cell` simulations model the cells divide into a parent and offspring cell. The concentration contents of the parent cell are duplicated and copied over to the offspring cell. In contrast, for the `complex_cell` model the concentration contents of the parent cell are either divided equally between the parent and offspring or duplicated and a further "speciesDivisionRules.csv" file is needed for each cell type to control the sharing behaviour.

The cell population may be defined using the file system (see Section S2.3) or by providing the population size. The size is provided through the `number_of_cells_high` and `number_of_cells_across` configuration parameters. These parameters are used to construct a honeycomb mesh of length `number_of_cells_high` and width `number_of_cells_across` with the origin of the mesh compared to the PDE domain provided by `cell_mesh_origin`. The `cell_mesh_origin` value is added to the x and y direction to translate the positions of the nodes in the cell mesh. The edges of the mesh denote which cells are nearest neighbours and whether these cells interact depends on the distance between the cells. These neighbours interact if their positions are within the cut off distance, `node_cuttoff_length`, and the strength of the

linear Hookean force for the interaction is provided by `linear_force_cutoff`.

S2.2 Configuration files for Domain only simulation

For reaction-diffusion simulations, the user provides a set of files specifying the domain topology, boundary conditions (BCs), initial conditions, and reactions systems (see examples in Figures S6–S7). These files are provided to ChemChaste by defining their file paths in the configuration file and an associated directory structure (see examples in Figure S5a Figure S5b).

a)

```
# simulation
output_filename = ChemChaste/SchnackenbergCases/CaseA
simulation_end_time = 100.0
number_of_reaction_pdes = 2
spatial_dimensions = 2
FE_element_dimension = 2

# bulk
domain_file_root = /home/chaste/projects/ChemChaste/DataInput/Data/SchnackenbergCases/CaseA/
domain_file = Domain.csv
domain_key_file = DomainKey.csv
ode_file = NodeSelector.csv
ode_key_file = OdeReactionFileKey.csv
diffusion_database = DiffusionDatabaseFile.csv
initial_conditions = InitialConditionFile.csv
boundary_conditions = BoundaryConditionFile.csv
```

b)

```
SchnackenbergCases
  CaseA
    BoundaryConditionFile.csv
    DiffusionDatabaseFile.csv
    Domain.csv
    DomainKey.csv
    InitialConditionFile.csv
    NodeSelector.csv
    OdeReactionFileKey.csv
    SchnackenbergReactionFile.txt
```

Figure S5: The configuration file a) and overall directory structure b) for simulating a reaction-diffusion system. a) The configuration file containing the basic simulation parameters; output directory, simulation end time, number of chemical PDEs to simulate, the domain and FE element dimensions. The configuration file also contains the directory paths and names of the different files used in the simulation. The file structure used during the domain only simulation b). The file paths are defined within the configuration file and follow CSV file types for parameters and defining the domain while TXT files are used for writing the reactions.

S2.2.1 Building the domain with chemicals and setting the properties

Users can define the domain in a series of CSV files.

"Domain Key" and "Domain Information" CSV files: These two files together define the domain topology. The "Domain Key" file introduces numeric id's for different 'types' of sub-domains, which might be associated with different chemical diffusion rates. Here, we use an example to define two sub-domains within the domain as 'bulk' and 'film', e.g. to mimic a bulk and biofilm environment. The "Domain Key" file simply lists names for such sub-domains and associates them with a numeric id. In the example given in Figure S6a-c, we defined two sub-domains labelled as 'Bulk', id 1, and 'Film', id 2, and then distributed them on the domain in such a way that the left section is 'bulk' and right section is 'film'. Note that the "Domain Information" file is organised as a 2D matrix, which is mapped on to the mesh implemented in

the FE simulations. This mapping stretches each matrix entry into 10 elements in the FE mesh. That is, a 10x10 file matrix would map onto a 100x100 FE mesh.

"Boundary Condition" and "Diffusion Database" csv files: Chemicals that diffuse and react within the domain are first defined in a "Boundary Condition" file. This file introduces each chemical in the system and sets the boundary conditions for them. In the example given in (Figure S6, we define two chemicals, U and V, and set the boundary conditions for both as zero-Neumann (zero-flux). Each chemical's diffusion in the different sub-domains (in this example, 'film' and 'bulk') are then defined in a file called, "Diffusion Database". Here, the user can use the defined sub-domain names (see above) to then specify the diffusion rate for each chemical in that sub-domain (see Figure S6d). Occasionally, a model may require the diffusion of a chemical to be completely inhibited in a specific sub-domain, this may be done by setting the diffusion rate value to 0.

"Initial Condition" csv file: The initial concentrations of each chemical in each sub-domain are given in a file called 'Initial Condition'. The file should specify one initial concentration value for each chemical and for each sub-domain (Figure S6e). This initial value is then applied to all mesh nodes associated with that sub-domain (see equation 4. While this approach provides a homogenous condition across all nodes of a given sub-domain, the values at the individual nodes may be perturbed through the addition of random noise value defined on a nodal basis during the setup of the simulation. That is, for states U, V with initial value u_0, v_0 for a given sub-domain, Ω_{sub} the perturbed initial values $U(x, 0), V(x, 0)$ are given by;

$$U(x, 0) = u_0 + X \text{ and } V(x, 0) = v_0 + Y \tag{11}$$

where the node location $x \in \Omega_{sub}$ and $X, Y \sim Uniform(-1, 1)$ are uniformly distributed random noises on the interval $[-1, 1]$. This random perturbation occurs only if the perturbation option in the initial condition file is set to `true` (see Figure S6e).

a) Domain.csv

```
# Domain Label Matrix
1,1,1,1,1,2,2,2,2,2
1,1,1,1,1,2,2,2,2,2
1,1,1,1,1,2,2,2,2,2
1,1,1,1,1,2,2,2,2,2
1,1,1,1,1,2,2,2,2,2
1,1,1,1,1,2,2,2,2,2
1,1,1,1,1,2,2,2,2,2
1,1,1,1,1,2,2,2,2,2
1,1,1,1,1,2,2,2,2,2
1,1,1,1,1,2,2,2,2,2
```

b) BoundaryConditionFile.csv

```
# Boundary conditions on the full domain edge:
# Variable, Type, Value
U,Neumann,0.0
V,Neumann,0.0
```

c) DomainKey.csv

```
# Domain Label Key
1,Bulk
2,Film
```

d) DiffusionDatabaseFile.csv

```
# Heterogeneous Diffusion rates
# Variable, Domain, Value
U,Bulk,1e-4
U,Film,1e-4
V,Bulk,1
V,Film,1
```

e) InitialConditionFile.csv

```
# initial conditions for the states:
# Variable, Domain, Value, Perturb?
U,Bulk,2.5,true
U,Film,2.5,true
V,Bulk,1.0,true
V,Film,1.0,true
```

Figure S6: Files defining the domain topology and providing simulation parameters. a) CSV file defining the domain topology. The nodes on the domain are labelled according to this matrix where sub-domains are specified by using different labels. The size of the matrix specified in this file is directly proportional to the size of the simulation mesh after scaling. Therefore a rectangular domain specified in this file will produce a rectangular domain mesh. b) Boundary conditions are implemented on the outside nodes of the FE mesh. The conditions are specified as "state variable", "BC type", and "BC value". c) Domain key file, which connects the 'simple' labels used in the domain topology file with the domain names. d) Diffusion in the simulations is modelled as isotropic diffusion where the coefficient value is the same in all directions. These values for each state variable are provided for all sub-domains names in c). e) The initial conditions in the domain are defined for each state variable (chemical) on each sub-domain. The value can differ for each sub-domain and the user can specify whether to perturb the value by a uniform random value.

S2.2.2 Chemical reactions and the "Reaction Information" and "ODE Reaction Key" CSV files

To define domain reactions the user provides a set of files, similar to those used for defining sub-domains. The user first creates a file called "ODE Reaction Key", specifying a reaction file name and an associated numeric id for that reaction (see Figure S7b). The reaction id can then be used to specify the nodes that are associated with specific reactions in the domain. This information is provided in a file called "Reaction Information", which should provide a matrix of the equal size to that given in the "Domain Information" file, where the reaction id's on the nodes are listed (Figure S7a). In the provided example, we have defined a reaction with reaction id 0 and attached this reaction to all of the nodes in the domain. Details of each of the implemented reactions are provided in a separate TXT file, which is referred to in the "ODE Reaction Key" file. In this example, we called this file "Schnackenberg Reaction" file. This file describes the reactions among the chemical species (see previous section), as well as the associated reaction parameters (Figure S7c). This description file utilises the provided reaction types and reaction parameters, as detailed in Section S1.3. Note that, as seen in the provided example, the file describing the ODE reactions can feature multiple reactions and all of the reactions defined in the file will be performed on the associated nodes (see Figure S7).

a) NodeSelector.csv

```
# Reaction Label Matrix
0,0,0,0,0,0,0,0,0,0
0,0,0,0,0,0,0,0,0,0
0,0,0,0,0,0,0,0,0,0
0,0,0,0,0,0,0,0,0,0
0,0,0,0,0,0,0,0,0,0
0,0,0,0,0,0,0,0,0,0
0,0,0,0,0,0,0,0,0,0
0,0,0,0,0,0,0,0,0,0
0,0,0,0,0,0,0,0,0,0
0,0,0,0,0,0,0,0,0,0
```

b) OdeReactionFileKey.csv

```
# Reaction File Label Key
0,SchnackenbergReactionFile.txt
```

c) SchnackenbergReactionFle.txt

```
# Reaction Type : Chemical Equation ; Rate Parameters
MassActionReaction : 2U + V -> 3U ; kf = 0.1
MassActionReaction : 0 <-> U ; kf = 0.1 kr = 0.2
MassActionReaction : 0 -> V ; kf = 0.3
```

Figure S7: The implementation of bulk reactions in user defined files. a) A CSV file defining the numeric ID of reaction files, which describe a series of chemical reactions. The reactions defined in a file associated with a node will determine those reactions to be active on that node. This allows for the creation of reaction sub-domains, which are not necessarily the same as the diffusion sub-domains. b) A reaction key file that connects the numeric IDs used in part a) to actual reaction file names. These names refer to TXT files containing the reaction system that are to occur on the associated nodes (creating the reaction sub-domain). c) An example reaction file, `SchnackenbergReactionFile.txt`. Each line denotes a separate reaction. Reactions are defined using a standard form composed of: “rate law", “ : " rate delimiter, reaction equation, “ ; " reaction delimiter, and the rate law parameters.

S2.3 Defining a cell simulation - Cooperator-cheater system

The hybrid continuum-discrete models of cells coupled to a bulk require a modified file structure and additional files. In particular, the cell-coupled simulation defines an additional cell mesh whose nodes denote the cell locations. The cell nodes are associated with a cell type and a cell object is formed with the cell properties and reactions corresponding to that cell type label. The simulation parameters for a cell-coupled simulation are presented in Figure S8 with the directory file structure given in Figure S9. The directory structure is such that the domain files are stored within a `DomainField` sub-directory of the simulation directory (Figure S9a).

```
# simulation
output_filename = ChemChaste/ChemChasteExample
simulation_end_time = 10.0
number_of_reaction_pdes = 4
spatial_dimensions = 2
FE_element_dimension = 2

# bulk
domain_file_root = /home/chaste/projects/ChemChaste/DataInput/Data/ChemChasteExample/DomainField/
domain_file = Domain.csv
domain_key_file = DomainKey.csv
ode_file = NodeSelector.csv
ode_key_file = OdeReactionFileKey.csv
diffusion_database = DiffusionDatabaseFile.csv
initial_conditions = InitialConditionFile.csv
boundary_conditions = BoundaryConditionFile.csv

# cell
cell_file_root = /home/chaste/projects/ChemChaste/DataInput/Data/ChemChasteExample/Cell/
cell_file = CellLayerTopology.csv
cell_key_file = CellLayerKey.csv

# mesh
cell_mesh_origin = -14.0
linear_force_cutoff = 1.5
```

Figure S8: The configuration file for the cell-coupled simulations. This configuration file defines the directory paths to associated files and includes some parameters specific to cell-coupled simulation. The files, accessible via the defined directory paths, define the structure and sub-populations of cells; the information is stored in the files `cell_file_root`, `cell_file`, `cell_key_file`. See example given in Figure S10. Simulation parameters used to couple the cell and domain mesh are also provided. `"cell_mesh_origin"` denotes the origin of the cell population structure with respect to the domain mesh. `"linear_force_cutoff"` is used as an interaction strength parameter for the Hookean linear spring force which connects the cells in the simulation.

S2.3.1 Including cells into the simulation

The `coupled_cell` and `complex_cell` simulation types add cells to the reaction-diffusion domain. Each cell has a type and the properties of each type are stored within a series of files contained in a directory, Figure S9a. The population is given a structure by constructing a mesh and associating a cell object to each node. Example files defining the mesh dimensions and cell-node labelling can be seen in Figure S10a–b. The initial structure of the cell population, at simulation start, is provided as a matrix in the `"CellLayerTopology"` CSV file (Figure S10a). The matrix is required to be smaller than that of the domain (Figure S6). The entries in this matrix refer to the type of a single cell which are mapped onto honeycomb mesh of equal length and width to the input matrix. If an input matrix is not specified and instead the configuration parameters, `number_cells_high` and `number_cells_across`, are provided then these values are used for the length and width of the honeycomb mesh. The cells are labelled by a numeric ID with cell type names provided in the `"CellLayerKey"` file. In the example shown in (Figure S10 two cells are defined with one cell of each type in the domain and are then provided the names 'CellA' and 'CellB'. These names are also used as sub-directory names for the following cell specific files. The initial cell concentrations and the concentration thresholds for the chemicals within the cell are given with a separate file for each cell type (see Figure S9b–c). The concentration thresholds provide a lower and upper bound for each of the chemical concentrations, which are used to implement cellular 'rules'. If the cellular concentration falls below the lower threshold the cell

is marked for death while cells with concentrations above the upper threshold will undergo cell division. The cell reaction systems are provided (Sections S1.3 and S1.4) see Figure S10c–e. The reactions occurring within the cell are located in the "Srn" text file while the transport and membrane reactions are located in the "TransportReactions" and "MembraneReactions" files.

As with the nodal initial conditions for the reaction-diffusion simulation, the initial conditions for the cellular concentrations may be perturbed by adding a random noise value at the beginning of the simulation. For the concentration $u(t)$, with initial concentration u_0 and with the perturbation option set to `true`, the starting cell concentration is given by;

$$u(t = 0) = u_0 + X \tag{12}$$

where $X \sim Uniform(-1, 1)$ is uniformly distributed random noise on the interval $[-1, 1]$.

During the simulation the conditions for cell division and death are determined by the species threshold file, Figure S10c. Each chemical in the cell reactions is provided with a maximum, u_{max}, and a minimum, u_{min}, concentration threshold. If the concentration reaches the threshold u_{max} for at least one chemical the cell division process is triggered. If the simulation type is set to `coupled_cell` then the cell concentrations are duplicated and copied during division. For the `complex_cell` simulation the cell contents of the parent are shared with the offspring. That is for each cell chemical c of parent concentration u_c at division the new concentrations u_c^{parent} and $u_c^{offspring}$ are given by

$$u_c^{parent} = u_c \text{ and } u_c^{offspring} = u_c$$

for the `coupled_cell` simulation type and for the `complex_cell` simulation type

$$u_c^{parent} = pu_c \text{ and } u_c^{offspring} = (1 - p)u_c$$

where p is the splitting ratio ($p = 0.5$ by default). Cell death is implemented by the removal of a cell and its associated cell mesh node. This apoptosis process is triggered when the concentration of a chemical falls below the minimum threshold for that species, $u \leq u_{min}$. To remove this death functionality, users should set $u_{min} = 0.0$. This will prevent the apoptosis process being triggered by that chemical. To remove the cell division functionality, users should set $u_{max} \leq u_{min}$ or $u_{max} = 0.0$.

In the example presented in Figure S9 we define two cell types, $\{CellA, CellB\}$, and the corresponding cell directories. The initial conditions and species thresholds are presented for $CellA$ covering each chemical found in the reactions, Figure S9. The cell division/death processes are dependent on the threshold values for each chemical but this has been set to ignore all of the chemicals, by setting both the upper and lower thresholds to zero, except for `Biomass` which will trigger division at a concentration $u_{max} = 1.5$ and apoptosis at $u_{min} = 0.1$.

a)

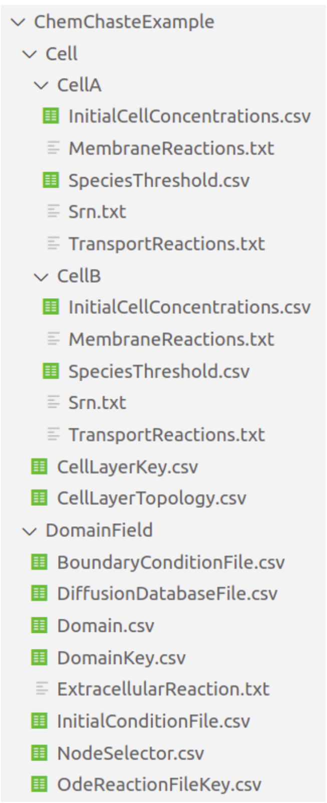

b) CellA/InitialCellConcentrations.csv

```
# Initial cell concentrations
# Chemical, Concentration, Perturb?
E,1.0,false
S,0.0,false
ES,0.0,false
Oxygen,1.0,false
NAD,0.5,false
NADH,0.5,false
ADP,0.5,false
ATP,0.5,false
H2O,1.0,false
Precursor,1.0,false
Biomass,1.0,false
```

c) CellA/SpeciesThreshold.csv

```
# Cell chemical concentration bounds
# Chemical, Maximum, Minimum
E,0.0,0.0
S,0.0,0.0
ES,0.0,0.0
Oxygen,0.0,0.0
NAD,0.0,0.0
NADH,0.0,0.0
ADP,0.0,0.0
ATP,0.0,0.0
H2O,0.0,0.0
Precursor,0.0,0.0
Biomass,1.5,0.1
```

Figure S9: Description of directories and cell properties files for the cell-coupled simulations. a) The directory structure for a cell-coupled simulation. Files relating to the domain structure, as detailed in Figure S6, are contained within `DomainField` directory. The cell files are provided within the `Cells` directory. Each cell type is provided its own sub-directory, with the same name as the label name in the `CellLayerKey.csv`, Figure S10d. For each cell type we define an initial concentrations file b) and a species threshold file c). b) The initial conditions are provided for each cellular state variable (chemical) following the form; name, value, and whether to perturb the initial value on a nodal basis. c) The threshold values for each cellular state variable (chemical) are provided in the order; name, maximum value, minimum value.

a) CellLayerTopology.csv

```
# Cell population
2,2,1
1,1,2
1,1,2
```

b) CellLayerKey.csv

```
# Cell label key
1,CellA
2,CellB
```

c) CellA/Srn.txt

```
Reaction Type : Chemical Equation ; Rate Parameters
MassActionReaction : ES -> E + S ; kf = 1.0
MassActionReaction : S + NAD + ADP -> Precursor + NADH + ATP ; kf = 1.0
MassActionReaction : NADH + ADP + Oxygen <-> NAD + H2O + ATP ; kf = 1.0 kr = 1.0
MassActionReaction : Precursor + ATP -> Biomass + ADP ; kf = 1.0
MassActionReaction : Precursor + ATP -> E + ADP ; kf = 1.0
```

d) CellA/TransportReactions.txt

```
# Transport Type : Bulk Chemical <-> Cell Bound Chemical ; Rate Parameters
MassActionTransportReaction : Oxygen <-> Oxygen ;  kf = 1.0 kr = 1.0
MassActionTransportReaction : ES <-> ES ;  kf = 1.0 kr = 1.0
MassActionTransportReaction : E <-> E ;  kf = 1.0 kr = 1.0
```

e) CellA/MembraneReactions.txt

```
# Membrane Reaction Type : Bulk Chemical Equation | Cell Chemical Equation ; Rate Parameters
MassActionCoupledMembraneReaction : S <-> S | S <-> S ;  kf = 1.0 kr = 1.0
```

Figure S10: The files associated needed to form the cell mesh and populate the cells with chemical reactions. These files are to be placed in the Cell sub-directory of the ChemChaste main directory (see Figure S9a). a) The "`CellLayerTopology.csv`" file provides the information for the initial cell mesh topology. It is written in the same format as the domain layer FigureS6a. In this example, a rectangular mesh of two cells is defined where the first cell is labelled "`1`" and the second labelled "`2`". This mesh is aligned with the domain mesh through translating the origin of the cell mesh as specified by the `cell_mesh_origin` parameter in the configuration file (see Figure S8). b) The "`CellLayerKey.csv`" files contains the key mappings from the numeric ID label used in "`CellLayerTopoogy.csv`" to the cell type used for the directory names. In this example two cell types are used $CellA, CellB$. The cellular reactions for $CellA$ are provided in three TXT files; c) the internal reactions, d) the transport reactions, and e) the membrane reactions. c) The "`Srn.txt`" file contains the cellular reaction system. Each line contains one reaction. The reactions are written in the standard form; reaction kinetic law, “ : " reaction law delimiter, reaction chemical equation, “ ; " parameter delimiter, kinetic law parameters. d) The file "`TransportReactions.txt`" lists the reactions/processes that couple the cells to the external domain. The reactions in this file are written in such a way that the chemicals on the left hand side represent the species in the bulk domain, while those on the right hand side represent the species inside the cell. Otherwise they follow the form in c) but using an appropriate set of reaction rate laws. e) The file "`MembraneReactions.txt`" lists the reactions that are coupled at the cell membrane and with reaction occurring on outside of the cell and one on the inside of the cell. These reactions are separated by the membrane delimiter, " | ", when written and the reaction rate law belongs to the membrane reaction set.

S2.3.2 Relation between ChemChaste and the COMBINE and SBML standards

Simulation standardisation is a key requirement for model reproducibility and the ability to implement the model is a range of simulation enhances this. Two model standards have been developed to support portable simulations; COMBINE archives (Walker and Southgate, 2020) and SBML (Hucka *et al.*, 2003). However, these standards are not currently supported by ChemChaste. There is some SBML support in the Chaste trunk code; in particular, preliminary

work has investigated using libSBML to parse SBML files and create Chaste-compatible C++ classes specifying subcellular reaction networks (Romijn *et al.*, 2020). Similar conversions may be implemented for ChemChaste.

To support SBML in ChemChaste, a user would need to write a reader/writer for the SBML code. This conversion could be easily performed to convert the ChemChaste chemical reaction systems into SBML level 1 specification. Under this conversion a SBML compartment would form each cell type and environment region while the reaction rules would have to draw from a set of compiled ChemChaste reaction classes. For complex SBML rules not native to ChemChaste a new reaction class may need implementing.

For the SBML-spatial specification, the continual contiguous domains are geometrically displayed in the ChemChaste `Domain.csv` file while the equivalent `DomainTypes\verb` are in the ChemChaste `DomainKey.csv`. SBML Diffusion coefficents and boundary conditions are provided in the ChemChaste `DiffusionDatabaseFile.csv` and `BoundaryConditionFile.csv`. The ChemChaste (isotropic) diffusion coefficients are provided on a domain type basis like SBML but the ChemChaste boundary conditions act on the simulation boundary, unlike SBML's domain-domain boundary conditions. Therefore, a conversion ChemChaste to SBML would need to introduce domain-domain boundaries which may be non-trivial. Furthermore, the current version of ChemChaste only supports Neumann or Dirichlet boundary conditions while SBML also supports Robin boundary conditions. ChemChaste (and Chaste) does not support Advection transport therefore the SBML `AdvectionCoefficient` class would be unsupported. Conversely, specifying the spatial arrangement of a population of cells is not supported by SBML but may be implemented in ChemChaste in the `CellLayerTopology.csv`. Overall, some new additions and considerations are needed for full implementation of SBML modelling using the ChemChaste system. Thus, this can be provided in future releases.

Simulation settings, metadata, and file names (corresponding to the manifest of COMBINE archives) are provided in the individual simulation configuration files. However, with the necessity of including an SBML file within the archive to describe the model the full use of COMBINE is not readily implemented.

The production of SBML files is often performed using other software systems that allow for rapid construction and investigation of system dynamics, one such software is Antimony (Smith *et al.*, 2009). Antimony provides an interface (graphical or text-file) to produce SBML models from a set of chemical reactions and reaction compartments. In Antimony, reactions are described by assigning a name, chemical equation, and mathematically describing a rate law with reaction parameters and initial conditions are provided as separate variables. While ChemChaste implements a similar text-based reaction input, and cell and domain reactions systems take the place of the "compartments" of Antimony and SBML, a conversion code would need to implement a rate law. This is because a Mass action kinetic law in ChemChaste is an instance of the `MassActionReaction` and the associated chemical equation provides product and reactants to determine the rate law, in Antimony the mathematical form is predetermined. Conversely, to translate an Antimony reaction into a ChemChaste model a series of reaction classes may need writing as the current version of ChemChaste does not support the direct input of an arbitrary reaction rate rule until the user writes the necessary classes. The method deployed by ChemChaste has been designed to facilitate a wider user audience hence the decision to use named laws rather than having a user write mathematical expressions. Using predefined laws also reduces potential implementation errors as the behaviours of these laws can be unit tested separately.

Overall, the conversion of a ChemChaste model into the Antimony/SBML/COMBINE hierarchy is possible through case-by-case writing of the reaction systems. As ChemChaste provides more complex user models and ABM properties but no advection or SBML Level 3 support there will be a loss of function when rewriting in either direction. However, merging ChemChaste into the standard may be possible using simple python scripts and may be

implemented in future ChemChaste releases.

S3 Adding a new reaction rate law

Chemical reactions occurring in the cell or in the domain follow a set of reaction rate laws defined in Table S1. However, new chemical reaction types may be added by the user and then freely implemented within the reaction system files. To introduce a new reaction rate, a new reaction header, which inherits from a previous reaction class, needs to be created and placed into the inheritance hierarchy (Figure S2). The user would then populate the class, update the inherited virtual functions, and then update the `ReactionTablet` function within the `ReactionTypeDatabase` file. From there on, the reaction rate type may be called by name, in the same manner as with the in-built reaction rates; e.g `MassActionReaction`.

The reaction header file needs to include the falling classes as “includes"; `AbstractChemical`, `AbstractChemistry`, and `AbstractReaction`. This will allow the class definitions to inherit publicly from the core reaction types. For example, `AbstractReaction` for a general irreversible reaction or `AbstractReversibleReaction` for a general reversible reaction. This inclusion is needed (useful), since these inherited reaction types provide a set of virtual functions that are useful to construct the reaction mechanism. In the following, we briefly describe these virtual functions, which the user will have to consider changing when creating a new rate law.

1. `React()`
    - Function description: Virtual function that implements the core dynamics of a chemical reaction. This function takes in the current system concentrations and outputs a vector describing the change in concentrations for the next timestep. The function calls `UpdateReactionRate()` function and then applies the reaction rate to the reaction stoichiometry, so to calculate the change in the species concentration.
    - Function input variables:
        - |systemChemistry (AbstractChemistry*)|
        - |currentChemistryConc (const std::vector<double>&)|
        - |changeChemistryConc (std::vector<double>&)|
    - Note: It might not be necessary to change this virtual function when creating a new rate law. This is because calculating the change in the chemical concentrations as the product of the reaction rate and stoichiometry vector is a standard method for implementing chemical reactions in a dynamical simulation. However, this function is explained here so that users are aware of it and can have the flexibility to implement other modelling approaches to chemical reactions by changing it.
2. `UpdateReactionRate()`
    - Function description: This function calculates the reaction rate scalar value for a given reaction. If the reaction being operated on is an irreversible reaction inheriting from `AbstractReaction`, then this function calculates the forward reaction rate, `forward_rate`, and calls the function `SetReactionRate(forward_rate)`. If the reaction being operated on inherits from `AbstractReversibleReaction` and has a calculated reverse reaction rate, `reverse_rate`, then this function calls both of the functions `SetForwardReactionRate(forward_rate)` and `SetReverseReactionRate(reverse_rate)`.
    - Function input variables:
        - |systemChemistry (AbstractChemistry*)|
        - |currentChemistryConc (const std::vector<double>&)|

- Note: This function is the main function to modify when creating a new reaction rate type. It essentially determines how reaction rates are calculated from current system concentrations and stoichiometries. The `MassActionReaction` class, which is of the reversible reaction type, utilises the current system concentrations to calculate reaction flux values in both reaction directions. The reaction rate values are calculated for use in the `React()` method (see Table S1.

3. `GetReactionType()`

   - Function description: This function returns a string type which provides the name of the reaction type; for example returning `MassActionReaction` or `SpectatorDependentReaction`. This function may also be used for reaction tracking purposes, but in the current implementation, it is used in the `ReactionTablet` function. This name needs to be the name in which the reaction files label the reaction type in order for the correct class calls to be made.

4. `UpdateReaction()`

   - Function description: This is a void function with no inputs. It is provided for the case that a reaction's behaviour needs to be altered outside of the `React()` function. Possible utilities include a switching of behaviour in reaction style based on concentrations, time, or system properties.

5. `ParseReactionInformation()`

   - Function description: This function parses the parameters and variables needed to process a reaction. In terms of the written file reaction, these data values occur in the string after the ; delimiter. This string is parsed into the data values provided by the user using a string delimiter. The user needs to identify the delimiter of this string as a member value in the reaction class. The values parsed are to be also stored as member values and may be utilised in the `UpdateReactionRate()` function where necessary.
   - Function input variables:
     - `reaction_information (string)`
     - `IsReversible (bool)`

S4 Adding a new transport process law

The cells and the reaction-diffusion domain are coupled through the transport processes transferring chemical species to either side of the cell membrane, see Section S1.4. As these processes require the chemical concentrations of both the cellular species and the corresponding external species at that domain location to be known, transport rules have a different construction to the reaction rate laws described in the previous section. ChemChaste has a set of transport rates already defined, Table S2, but new transport process rates may be added by the user and integrated with ChemChaste.

Transport processes in ChemChaste follow the general form of a reaction where the `Substrates` are chemical species in the domain and the `Products` are species in the cell. For the introduction of a new transport process, users would need to create a new transport reaction header, which inherits from a previous transport reaction class (see Figure S3a for the inheritance structure for transport reactions).

Within the new transport reaction the user would populate the class with updated inherited virtual functions, then update the `TransportTablet` function within the `ReactionTypeDatabase`

file. From there the reaction type may be called by name in the same manner as the in-built reactions; i.e `MassActionReaction`.

The reaction header file needs as 'includes'; `AbstractChemical`, `AbstractChemistry`, and the appropriate abstract transport reaction base. The available abstract bases are; `AbstractTransportReaction` for single direction domain to cell transport, `AbstractTransportOutReaction` for single direction cell to domain transport, or `AbstractReversibleTransportReaction` for reversible domain-cell transport. Depending on the abstract base selection, the class definitions needs to inherit publicly from the base reaction types. These inherited base reaction types provide a set of virtual functions which control the reaction mechanism. In the following, we briefly describe these virtual functions, which the user will have to consider changing when creating a new transport rate.

1. `React()`
   - Function description: Virtual function that performs the transport reaction. This function takes in the current bulk domain and cell concentrations and computes a vector describing the change in both concentration sets over the next timestep, i.e. the reaction rate. The function calls `UpdateReactionRate()` function, which multiples the reaction rate with the reaction stoichiometry to update the species concentrations.
   - Function input variables:
     - `bulkChemistry (AbstractChemistry*)`
     - `cellChemistry (AbstractChemistry*)`
     - `currentBulkConcentration (const std::vector<double>&)`
     - `currentCellConcentration (const std::vector<double>&)`
     - `changeBulkConc (std::vector<double>&)`
     - `changeCellConc (std::vector<double>&)`
   - Note: It might not be necessary to change this virtual function when creating a new transport rate. The input variables `bulkChemistry` and `cellChemistry` refer to the chemical species outside the cell in the domain and inside the cell respectively. The `bulkChemistry` species refer to the `Substrates` of the transport reaction and the `cellChemistry` refers to the `Products` of the reaction.
2. `UpdateReactionRate()`
   - Function description: This function calculates the reaction rate scalar value for the given reaction. If the reaction being operated on is an irreversible reaction inheriting from `AbstractTransportReaction` or `AbstractTransportOutReaction`, then this function calculates the forward reaction rate, `forward_rate`, and calls the function `SetReactionRate(forward_rate)`. If the reaction being operated on inherits from `AbstractReversibleTransportReaction` and has a calculated reverse reaction rate, `reverse_rate`, then this function calls both of the functions `SetForwardReactionRate(forward_rate)` and `SetReverseReactionRate(reverse_rate)`.
   - Function input variables:
     - `bulkChemistry (AbstractChemistry*)`
     - `cellChemistry (AbstractChemistry*)`
     - `currentBulkConc (const std::vector<double>&)`
     - `currentCellConc (const std::vector<double>&)`
   - Note: This function is the main area to modify for new reaction types.
3. `GetReactionType()`

- Function description: This function returns a string type which provides the name of the reaction type; for example returning `MassActionTransportReaction`. This function may also be used for reaction tracking purposes, but is currently used in the `TransportTablet` function. This name needs to be the same name in which the reaction files label the reaction type in order for the correct class calls to be made.

4. `UpdateReaction()`

   - Function description: Void function with no inputs provided for the case that a reaction's behaviour needs to alter outside of the `React()` function. Possible utilities include a switching of behaviour in transport style based on concentrations, time, or system properties.

5. `ParseReactionInformation()`

   - Function description: This function parses the parameters and variables needed to process the transport process. In terms of the written file reaction, these data values occur in the space after the ; delimiter. This string is to be parsed into the data values by the user using a string delimiter. The user needs to identify the delimiter of this string as a member value in this reaction class. The values parsed are to be also stored as member values and may be utilised in the `UpdateReactionRate()` function where necessary.
   - Function input variables:
     - `reaction_information (string)`
     - `IsReversible (bool)`

S5 Adding a new membrane reaction rate law

Besides reactions outside and inside the cell, chemical reactions may also be modelled as if occuring at the cell membrane in a way that couples cell interior and external chemicals. These reactions essentially couple two reactions, one occuring in the domain and another in the cell. These membrane reactions are described in Section S1.4, with the currently implemented membrane reaction rates in Table S3.

New membrane reaction rates may be added by the user by creating a new reaction class inheriting from an existing membrane reaction class (Figure S3b), overriding the inherited virtual functions. To integrate with the ChemChaste system the `MembraneTablet` function within the `ReactionTypeDatabase` file is updated with the new membrane reaction. From there the reaction type may be used by name in the same manner as the supplied membrane reactions; i.e `MassActionCoupledMembraneReaction`. As the membrane reaction couples two separate reaction systems, two reactions are provided per instance. These separate chemical reactions within the membrane reaction are separated by the | delimiter, with the external domain reaction before the delimiter and the internal cell reaction after.

The membrane reaction header file needs as “includes"; `AbstractChemical`, `AbstractChemistry`, and either of the base membrane reaction types `AbstractMembraneReaction` for irreversible reactions or `AbstractReversibleMembraneReaction` for reversible reactions. Coupling of a reversible and irreversible reaction is not currently implemented. The class definitions then inherit publicly from the appropriate base membrane reaction type. These inherited types provide a set of virtual function which control the reaction mechanism. In the following, we briefly describe these virtual functions, which the user will have to consider changing when creating a new membrane-bound reaction rate.

1. `React()`

   - Function description: Virtual function that performs the membrane reaction. This function takes in the current bulk domain and cell concentrations, and computes a vector for the change in both concentration sets over the next timestep. The function then calls the `UpdateReactionRate()` function, which applies the reaction rate to the stoichiometry to calculate the change in concentrations for both the domain and cell chemicals.
   - Function input variables:
     - `bulkChemistry (AbstractChemistry*)`
     - `cellChemistry (AbstractChemistry*)`
     - `currentBulkConcentration (const std::vector<double>&)`
     - `currentCellConcentration (const std::vector<double>&)`
     - `changeBulkConc (std::vector<double>&)`
     - `changeCellConc (std::vector<double>&)`
   - Note: Calling and modifying this function directly may not be necessary to implement a new membrane reaction rate. The input variables `bulkChemistry` and `cellChemistry` refer to the chemical species outside the cell in the domain and inside the cell respectively. These separate chemistries are formed from both the substrates and products of the separate chemical reactions.

2. `UpdateReactionRate()`

   - Function description: This function calculates the combined reaction rate scalar value for both the reactions at the membrane. After calculating the reaction rate, `forward_rate`, the function calls the function `SetReactionRate(forward_rate)` for an irreversible reaction inheriting from `AbstractMembraneReaction`. If the reaction inherits from `AbstractReversibleMembraneReaction` and has a calculated reverse reaction rate, `reverse_rate`, then both the functions, `SetForwardReactionRate(forward_rate)` and `SetReverseReactionRate(reverse_rate)`, are to be called.
   - Function input variables:
     - `bulkChemistry (AbstractChemistry*)`
     - `cellChemistry (AbstractChemistry*)`
     - `currentBulkConc (const std::vector<double>&)`
     - `currentCellConc (const std::vector<double>&)`

3. `GetReactionType()`

   - Function description: This function returns a string type which provides the name of the reaction type; for example returning `MassActionCoupledMembraneReaction`. This function may also be used for reaction tracking purposes, but it is currently used in the `MembraneTablet` function to set the name of the membrane rate type. This name needs to be the name in which the reaction files label the reaction type in order for the correct class calls to be made.

4. `UpdateReaction()`

   - Function description: This is a void function with no inputs provided for the case that a reaction's behaviour needs to alter outside of the `React()` function. Possible utilities include a switching of behaviour in transport style based on concentrations, time, or system properties.

5. `ParseReactionInformation()`

- Function description: This function parses the parameters and variables needed to process the transport process. In terms of the written file reaction, these data values occur in the space after the ; delimiter. This string is to be parsed into the data values by the user using a string delimiter. The user needs to identify the delimiter of this string as a member value in this membrane reaction class. The values parsed are to be also stored as member values and may be utilised in the `UpdateReactionRate()` function where necessary.
- Input variables:
  - `reaction_information (string)`
  - `IsReversible (bool)`

S6 Derivation of the Schnakenberg parameter sets

The Schnakenberg reaction system involves chemical species U and V that are produced, inter-converted, and removed via the reactions

$$0 \underset{k_{-1}}{\overset{k_1}{\rightleftharpoons}} \mathrm{U}, \tag{13}$$

$$0 \xrightarrow{k_2} \mathrm{V}, \tag{14}$$

$$2\mathrm{U} + \mathrm{V} \xrightarrow{k_3} 3\mathrm{U}, \tag{15}$$

where k_1, k_{-1}, k_2, k_3 denote reaction rate constants. Applying mass action kinetics to these reactions yields the ODE system

$$\frac{dU}{dt} = k_1 - k_{-1}U + k_3 V U^2 \equiv R_U(U,V), \tag{16}$$

$$\frac{dV}{dt} = k_2 - k_3 V U^2 \equiv R_V(U,V), \tag{17}$$

for the concentrations of U and V. The Schnakenberg reaction-diffusion system extends this model to include diffusion of U and V with constant diffusion coefficients D_U and D_V, respectively, leading to the set of coupled PDEs

$$\frac{\partial U}{\partial t} - D_U \nabla^2 U = R_U(U,V), \tag{18}$$

$$\frac{\partial V}{\partial t} - D_V \nabla^2 V = R_V(U,V). \tag{19}$$

The Schnakenberg model is a special case of the Gierer-Meinhardt activator-inhibitor model (Gierer and Meinhardt, 1972). Following previous mathematical analyses (Murray, 2003; Korvasová *et al.*, 2015; Guin *et al.*, 2012; Gambino *et al.*, 2013), we present necessary conditions for pattern formation via diffusion-driven instability (DDI) in this model. For a DDI to occur, we require the spatially uniform solution to (18)–(19) to be linearly stable in the absence of diffusion, but unstable in the presence of diffusion. In the absence of diffusion ($D_U = D_V = 0$), equations (18)–(19) have the unique steady-state solution

$$(U_0, V_0) = \left(\frac{k_1 + k_2}{k_{-1}}, \frac{k_2 k_{-1}^2}{k_3 (k_1 + k_2)^2} \right). \tag{20}$$

By requiring both eigenvalues of the Jacobian to have negative real part, we find that (20) is linearly stable in the absence of diffusion if

$$\frac{\partial R_U}{\partial U} + \frac{\partial R_V}{\partial V} < 0 \tag{21}$$

and

$$\frac{\partial R_U}{\partial U}\frac{\partial R_V}{\partial V} - \frac{\partial R_U}{\partial V}\frac{\partial R_V}{\partial U} > 0, \tag{22}$$

where the partial derivatives of R_U and R_V are evaluated at (20). By requiring at least one eigenvalue to have negative real part, we find that (20) becomes linearly unstable in the presence of diffusion if

$$D_V\frac{\partial R_U}{\partial U} + D_U\frac{\partial R_V}{\partial V} > 0 \tag{23}$$

and

$$\left(D_u\frac{\partial R_V}{\partial V} + D_V\frac{\partial R_U}{\partial U}\right)^2 - 4D_UD_V\left(\frac{\partial R_U}{\partial U}\frac{\partial R_V}{\partial V} - \frac{\partial R_U}{\partial V}\frac{\partial R_V}{\partial U}\right) > 0. \tag{24}$$

After some algebra, we find that the above conditions correspond to the following inequalities on the reaction rate constants k_1, k_1, k_2, k_3 and diffusion coefficients D_U, D_V:

$$\frac{D_V}{D_U}(2k_2 - 1)k_{-1}^3 > k_3(k_1 + k_2)^2 > 0. \tag{25}$$

The Schnakenberg reaction system admits both spatial patterning and oscillatory dynamics as shown in figure 3 of the main text. The onset of these two regimes may be seen by plotting a bifurcation diagram (see figure S11).

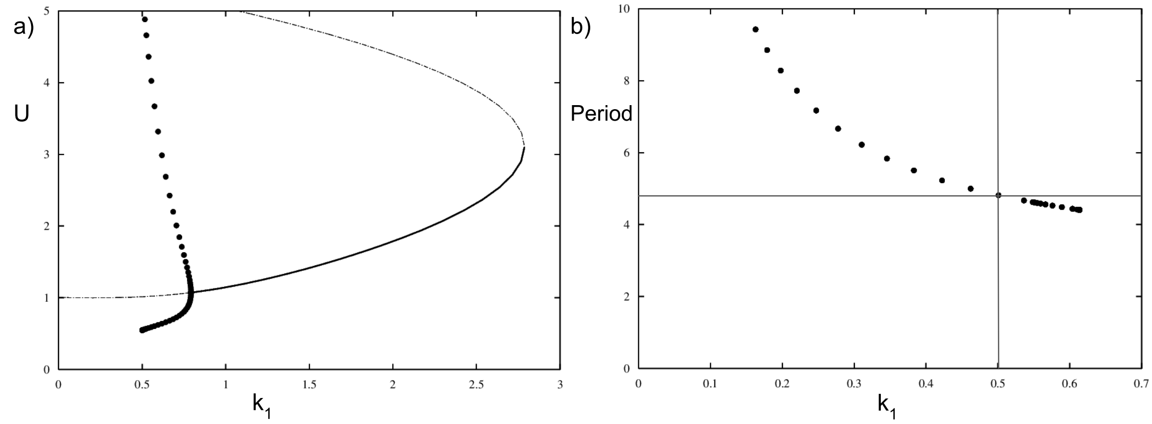

Figure S11: a) The bifurcation diagram for the Schnakenberg reaction system where reaction rate k_1 is used as the bifurcation parameter and displaying the fixed points for the variable U. The diagram displays a stable steady state where spatial patterning may be found ending in a Hopf bifurcation point ($k_1 = 0.61, U = 0.96$) with oscillations beyond this. b) The period of the oscillations for varying k_1 starting at the Hopf bifurcation point. The marked point indicates the k_1 value used to produce the oscillations in figure 3a of the main manuscript. The subsequent period of oscillations corroborate the ChemChaste output.

References

Gambino, G. *et al.* (2013). Pattern formation driven by cross-diffusion in a 2D domain. *Nonlinear Anal. Real World Appl.*, **14**, 1755–1779.

Gierer, A. and Meinhardt, H. (1972). A theory of biological pattern formation. *Kybernetik*, **12**(1), 30–39.

Guin, L. N. *et al.* (2012). The spatial patterns through diffusion-driven instability in a predator–prey model. *Appl. Math. Model.*, **36**, 1825–1841.

Hucka, M. *et al.* (2003). The systems biology markup language (SBML): a medium for representation and exchange of biochemical network models. *Bioinformatics*, **4**, 524–531.

Korvasová, K. *et al.* (2015). Investigating the Turing conditions for diffusion-driven instability in the presence of a binding immobile substrate. *J. Theor. Biol.*, **367**, 286–295.

Logg, A. *et al.* (2012). Automated Solution of Differential Equations by the Finite Element Method. *Springer*.

Murray, J. D. (2003). *Mathematical Biology II: Spatial Models and Biomedical Applications*. Springer.

Pathmanathan, P. (2012). Chaste: Finite Element Implementations. *https://chaste.cs.ox.ac.uk/trac/wiki/UsefulNotes*.

Romijn, L. B. *et al.* (2020). Modelling the effect of subcellular mutations on the migration of cells in the colorectal crypt. *BMC Bioinformatics*, **21**.

Shapira, Y. (2012). Solving PDEs in C++: Numerical Methods in a Unified Object-Oriented Approach. *SIAM*, **2nd ed.**

Smith, L. P. *et al.* (2009). Antimony: A modular model definition language. *Bioinformatics*, **25**, 2452–2454.

Walker, D. C. and Southgate, J. (2020). The first 10 years of the international coordination network for standards in systems and synthetic biology (COMBINE). *J Integr Bioinform*, **17**.
