# Supplementary material for: ChemChaste: Simulating spatially inhomogeneous biochemical reaction–diffusion systems for modeling cell–environment feedbacks
Source: Gigascience. 2022 Jun 17;11:giac051. doi: 10.1093/gigascience/giac051 (PMC9205757; doi:10.1093/gigascience/giac051)
Supplement: giac051_Response_to_Reviewer_Comments_Original_Submission [file giac051_response_to_reviewer_comments_original_submission.pdf]

Author's Response To Reviewer Comments

Close

See attached reply letter.

Close
