# Supplementary material for: ChemChaste: Simulating spatially inhomogeneous biochemical reaction–diffusion systems for modeling cell–environment feedbacks
Source: Gigascience. 2022 Jun 17;11:giac051. doi: 10.1093/gigascience/giac051 (PMC9205757; doi:10.1093/gigascience/giac051)
Supplement: giac051_Response_to_Reviewer_Comments_Revision_1 [file giac051_response_to_reviewer_comments_revision_1.pdf]

Author's Response To Reviewer Comments

Close

Dear Editorial office. We have now made the final edits requested on the manuscript. We hope that it is now ready for publication.

Close
