# Supplementary material for: ChemChaste: Simulating spatially inhomogeneous biochemical reaction–diffusion systems for modeling cell–environment feedbacks
Source: Gigascience. 2022 Jun 17;11:giac051. doi: 10.1093/gigascience/giac051 (PMC9205757; doi:10.1093/gigascience/giac051)
Supplement: giac051_Reviewer_2_Report_Original_Submission — Lutz Brusch -- 1/20/2022 Reviewed [file giac051_reviewer_2_report_original_submission.pdf]

Reviewer Report

Title: ChemChaste: Simulating spatially inhomogenous biochemical reaction-diffusion systems for modelling cell-environment feedbacks

Version: Original Submission **Date:** 1/20/2022

Reviewer name: Lutz Brusch

Reviewer Comments to Author:

The manuscript no. GIGA-D-21-00383, entitled "ChemChaste: Simulating spatially inhomogenous biochemical reaction-diffusion systems for modelling cell-environment feedbacks" addresses the important technical challenge of hybrid discrete-continuous models. The presented extension of the widely used Chaste software library, termed ChemChaste, now supports simulations of reaction-diffusion dynamics in a 2-dimensional environment bi-directionally coupled to motile and chemically active but point-like cells.
Specifically, ChemChaste supports arbitrarily many spatial domains within the system, each with individual uniform diffusion coefficients. It supports arbitrarily many coupled reaction-diffusion equations and coupling via membrane reactions and transport reactions between bulk molecular species and intracellular species. Cells are coarsely represented as points on a cell-mesh that is distinct from the FE-mesh for solving the reaction-diffusion dynamics. The user interface is established through a tree of many small text and csv files that are human-readable. All these extensions to Chaste are valuable and their presentation is important for the large user base and beyond. The manuscript is clearly structured and well written. The source code is openly available under the permissive BSD 3-clause license at the provided GitHub link (https://github.com/OSS-Lab/ChemChaste) and includes all models, parameters and data as used in the present manuscript.
As the motivation and title focus on "...modelling cell-environment feedbacks", then also the implications and limitations of the coarse cell representation in ChemChaste must be clearly stated, see comments below.
Major comments:
----
1. Coarse spatial cell representation:
Cells are represented by their node position in the cell-mesh and interact with the environment through a single node at the same position in the FE-mesh. Can this formalism properly account for transport reaction fluxes in strongly heterogeneous environments where the FE-mesh needs many nodes with differing field values in a spatial area equivalent to the size of a single cell (with the cell node inside this area)? For example, how does this formalism evaluate the uptake from an exponential concentration gradient (as is common for diffusion and degradation around a localized source). For such a field, the local concentration value at any single position is always smaller than the average over any symmetric interval around it. Hence a transport reaction flux calculated with the single concentration value at the cell center will systematically underestimate the flux that would result from averaging over the area equivalent to the size of the cell. Moreover, such systematic errors also occur for linear concentration

gradients and can get amplified when transport or membrane reactions are nonlinear with for instance high Hill coefficient. For comparison, with a spatially more explicit cell representation with many paired cell-nodes and field-nodes, one could directly sum the flux contributions from these paired field-nodes. But with the single cell-node here, usability seems limited to weak gradients at the scale of cell size. Alternatively, can a spatial kernel or stencil function be used to average or sum over field values in the spatial area equivalent to the size of a cell?

2. Conservation of mass for transport:

In biology, the number of molecules per time taken from the environment in a transport reaction has to equal the number of molecules per time added to the cell, and vice versa. So mass needs to be conserved and not concentration whereas ChemChaste seems to add and subtract the concentration flux in the different spatial compartments (cf. page 7 of SI.S1.4). For example, if the FE-mesh needs to use multiple nodes in a spatial area equivalent to the size of a single cell (hence Ve<Vc) but the transport reaction only relates the concentration value at one of these nodes to the cell-node, then mass is not conserved and results will be wrong. One option may be to attach volume attributes to nodes in both meshes. A node i in the cell-mesh would store the current cell volume Vc_i and a node j in the FE-mesh would store that node's share of the volume in the environment Ve_j (doubling the number of nodes in the FE-mesh would on average halve each node's volume Ve_j). Then secretion of molecules with intracellular concentration u at rate k would reduce the intracellular concentration by a flux of molecule number per per time and per volume, i.e. k*u*Vc/Vc=k*u, and increase the concentration at the environment node with flux k*u*Vc/Ve which in general is and must be different from the intracellular concentration flux k*u. Likewise, if the FE-mesh is coarse (hence Ve>Vc) then the transport flux must get diluted like k*u*Vc/Ve < k*u. The factor Vc/Ve does not appear to be implemented and the equations on page 7 of SI.S1.4 omit this factor, limiting the usability to the special case Vc=Ve. This implies that the construction of the FE-mesh has to match the cell-mesh wherever cells are positioned and in their neighborhood. This limitation and the required construction of the FE-mesh must be described.

3. Scaling of fluxes with cell surface area:

In biology, membrane reactions and transport reactions occur at the molecular scale and yield a characteristic flux density per membrane area. The total flux per cell is then the integral of the flux density over the cell surface. Hence cells with larger surface area must be able to exchange more molecules with the environment. Since differently shaped cells will have different surface to volume ratios, it appears necessary to attach not only a cell volume Vc_i to each node i of the cell-mesh but also a surface area value Ac_i. The transport reaction fluxes from item 2. above then become k'*Ac*u*Vc/Vc=k'*Ac*u and k'*Ac*u*Vc/Ve, respectively, with a new rate constant k' with units [1/(area*time)]. The same argument applies to membrane reactions. Only if all cells have the same and constant surface area then Ac does not need to be attached to nodes and k may be used instead of k'*Ac.

4. User interface and model format:

To improve Interoperability according to FAIR,

- please explore and comment how the files that are required for model definition in ChemChaste can or cannot be packaged in a COMBINE archive [Bergmann et al. (2014). COMBINE archive and OMEX format: one file to share all information to reproduce a modeling project. BMC Systems Biology 15:369. https://doi.org/10.1186/s12859-014-0369-z].

- please compare ChemChaste's declaration of the reaction-diffusion model in the environment to that of the SBML Level 3 Spatial Processes Package (SBML-spatial) [https://synonym.caltech.edu/documents/specifications/level-3/version-1/spatial/].
- please compare ChemChaste's declaration of the reactions to that of the Antimony model format as used in the Tellurium framework [Smith et al. (2009). Antimony: a modular model definition language. Bioinformatics 25:2452. https://doi.org/10.1093/bioinformatics/btp401].
- please discuss the necessary steps to convert model files available in SBML-spatial or Antimony to ChemChaste and vice versa.

5. Numerical accuracy of the 3-fold operator splitting scheme for cell-environment coupling:
As shown in Fig.1b, the three operators 1 (Cell dynamics), 2 (Environment dynamics), 3 (Cellular fluxes) are applied sequentially for a coupled cell-environment model. How is the numerical error controlled for this 3-fold operator splitting scheme? How are time steps chosen or adapted internally?

6. Model equations for test case with cell-environment coupling:
In SI, Figure S10.c (and file CellA/Srn.txt in the code repository) apparently all 5 reactions are defined as reversible with "<->" and each has a nonzero kr=1.0 but only two of these reactions are reversible in the reaction scheme in main Fig.4a. Probably the file in the repo and SI is wrong (as the reverse generation of Precursor directly from Biomass and Enzyme is not physiological) and possibly the simulation results in Fig.4b may change after correction of the file CellA/Srn.txt.

7. Findability of repository:
To improve Findability of ChemChaste according to FAIR, the code repo should be integrated with or referenced from the core project at https://github.com/Chaste/ . This integration should also facilitate future code maintenance and usability in a sustainable manner.

Minor comments:

\----------------------

8. Further tests may be easily implemented for the Schnakenberg model which was qualitatively simulated but not quantitatively compared to an analytical prediction (main text, lines 368-375). One (rough) quantitative comparison could be achieved for the dominant mode of the Fourier-transformed simulated pattern (Fig.3b; or some other measure of the spatial period of the pattern) versus the critical mode of the diffusion-driven instability ($|k_{cr}|^2 = 1/(2*D_U) * dR_U/dU + 1/(2*D_V) * dR_V/dV$). In addition, the instability threshold from eq. (25) in SI.S6 (page 27) can be tested in simulations along a one-parameter scan across the instability and the temporal oscillation period in Fig.3a can be (roughly) compared to the predicted period from the imaginary part of the eigenvalues of the steady state or computed by means of numerical continuation in AUTO (http://indy.cs.concordia.ca/auto).

9. Main text, lines 460-463: "Thus...lead to a spatial segregation of the two cell types." This behavior may be subject to the slow or lacking active motility of the cells. Now, cell division alone seems to generate compact clones of the same cell type instead of emergent spatial segregation. Maybe comment if/how ChemChaste handles random walks of cells or even chemotaxis of cells towards ES. Then the interesting question of emergent spatial segregation can be studied with ChemChaste.

10. Please clarify if/how ChemChaste allows to incorporate transport reactions directly between neighboring cells (like auxin or calcium transport in tissues)?

11. Where are the membrane reactions involving a cell and the environment included in Fig.1b: in steps 1./2. or in step 3.? That is interesting for the numerical operator splitting scheme and may be added to

the caption.
12. In addition to item 7. above (which should ensure future usability), the reproducibility of the current model results as presented in this manuscript should be ensured by archiving the current software version from the ChemChaste code repo at Zenodo or a similar service and the DOI of that archive should be given in the manuscript. In addition, that archived code shall be given a version number on GitHub and that version number shall also be given in the manuscript.
Figure improvements:

- Figure 2.b may have axes flipped or may have an unfortunate color scale with too little contrast for convergence scores between 0.4 and 0.5 to show the gradual change of score at the horizontal row with dt=0.1 (which is apparently used in Fig. 2.c and shows a change of accuracy there). Please check and improve the correspondence between panels b) and c) such that the data from panel c) helps to get a feeling for the L2 score changes in panel b).
- Figure 2.b: How can we understand the loss of convergence if the time step is reduced (say from 0.006 to 0.0002) at any fixed dx? From other solvers, one is used to that finer dt improve convergence while this plot shows dark (high L2 score) areas on both sides of the light (low L2 score) areas at intermediate values of dt.
- Figure 2.c: The color code is not suited for so many curves. Either include line style or reduce the number of curves (preferred). It must become clear which curve belongs to which dx. The green curve with dx=0.8 seems to be hidden?
- Figure 3.a: The figure caption should explain the source of variation between nodes (e.g. by pointing to the noise terms in eqs. 13,14) and the color code for the two bands (dark and light) around each curve (1-sigma and 2-sigma or 1-sigma and min/max ?).
- Figure 4b: These two panels could be given more space. Suggestion: re-arrange part a) horizontally and then put both diagrams of b) at the bottom, left and right.
- Figure 5: The caption wrongly announces "and t=100" which is not shown. Also the words "towards the" in the first line seem to be linked to t=100.
Text corrections:

- main text, line 61. The sentence "...centred on the role chemical coupling." seems to miss the preposition "of".
- main text, line 71. The phrase "cellular network reaction size" appears misleading, when it shall refer to "the size of the cellular reaction network".
- main text, lines 280, 284, 286: Since the subsections of the Results section are not numbered here, then the text pointers "(Section)" can be omitted.
- main text, one line below eq.(7): "reaction rate constants parameters" can drop the word "parameters"
- main text, lines 450 and 451: "a...concentrations" should be either singular or plural
- SI.S1, page 1, line 5 above eq. (1): text "exchange chemical concentrations" should read "exchange molecules" and, correspondingly, "controlling the chemical
concentrations passing between the bulk and the cell" should read "controlling the flux of molecules between the bulk and the cell".

- SI.S1, page 2, line 2: "asssociated" has an "s" too much
- SI.S1, page 5, at the end of Fig.S1's caption: $k-p$ should be k_p
- SI.S2.2.1, page 14, eq. (11) has capital U_0 and V_0 as initial values while the sentence above has small u_0, v_0. These should be the same symbols.
- SI.S6, page 26, 1 line below eq. (19): "is a spatial case" should be "is a special case"

Methods

Are the methods appropriate to the aims of the study, are they well described, and are necessary controls included? Choose an item.

Conclusions

Are the conclusions adequately supported by the data shown? Choose an item.

Reporting Standards

Does the manuscript adhere to the journal's guidelines on minimum standards of reporting? Choose an item.

Choose an item.

Statistics

Are you able to assess all statistics in the manuscript, including the appropriateness of statistical tests used? Choose an item.

Quality of Written English

Please indicate the quality of language in the manuscript: Choose an item.

Declaration of Competing Interests

Please complete a declaration of competing interests, considering the following questions:

- Have you in the past five years received reimbursements, fees, funding, or salary from an organisation that may in any way gain or lose financially from the publication of this manuscript, either now or in the future?
- Do you hold any stocks or shares in an organisation that may in any way gain or lose financially from the publication of this manuscript, either now or in the future?
- Do you hold or are you currently applying for any patents relating to the content of the manuscript?
- Have you received reimbursements, fees, funding, or salary from an organization that holds or has applied for patents relating to the content of the manuscript?

- Do you have any other financial competing interests?
- Do you have any non-financial competing interests in relation to this paper?

If you can answer no to all of the above, write 'I declare that I have no competing interests' below. If your reply is yes to any, please give details below.

I declare that I have no competing interests.

I agree to the open peer review policy of the journal. I understand that my name will be included on my report to the authors and, if the manuscript is accepted for publication, my named report including any attachments I upload will be posted on the website along with the authors' responses. I agree for my report to be made available under an Open Access Creative Commons CC-BY license (http://creativecommons.org/licenses/by/4.0/). I understand that any comments which I do not wish to be included in my named report can be included as confidential comments to the editors, which will not be published.

Choose an item.

To further support our reviewers, we have joined with Publons, where you can gain additional credit to further highlight your hard work (see: https://publons.com/journal/530/gigascience). On publication of this paper, your review will be automatically added to Publons, you can then choose whether or not to claim your Publons credit. I understand this statement.

Yes Choose an item.
